# A Multi-Compartment, Single and Multiple Dose Pharmacokinetic Study of the Vaginal Candidate Microbicide 1% Tenofovir Gel

**DOI:** 10.1371/journal.pone.0025974

**Published:** 2011-10-19

**Authors:** Jill L. Schwartz, Wes Rountree, Angela D. M. Kashuba, Vivian Brache, Mitchell D. Creinin, Alfred Poindexter, Brian P. Kearney

**Affiliations:** 1 CONRAD, Eastern Virginia Medical School (EVMS), Arlington, Virginia, United States of America; 2 Family Health International (FHI), Research Triangle Park, North Carolina, United States of America; 3 University of North Carolina Eshelman School of Pharmacy and University of North Carolina CFAR Clinical Pharmacology and Analytical Chemistry Core Laboratory, University of North Carolina, Chapel Hill, North Carolina, United States of America; 4 PROFAMILIA, Santo Domingo, Dominican Republic; 5 Center for Family Planning Research, University of Pittsburgh and Magee-Womens Research Institute, Pittsburgh, Pennsylvania, United States of America; 6 Advances in Health, Inc., Houston, Texas, United States of America; 7 Gilead Sciences, Foster City, California, United States of America; Institute for Genome Sciences, University of Maryland School of Medicine, United States of America

## Abstract

**Background:**

Tenofovir (TFV) gel is being evaluated as a microbicide with pericoital and daily regimens. To inhibit viral replication locally, an adequate concentration in the genital tract is critical.

**Methods and Findings:**

Forty-nine participants entered a two-phase study: single-dose (SD) and multi-dose (MD), were randomized to collection of genital tract samples (endocervical cells [ECC], cervicovaginal aspirate and vaginal biopsies) at one of seven time points [0.5, 1, 2, 4, 6, 8, or 24 hr(s)] post-dose following SD exposure of 4 mL 1% TFV gel and received a single dose. Forty-seven were randomized to once (QD) or twice daily (BID) dosing for 2 weeks and to collection of genital tract samples at 4, 8 or 24 hrs after the final dose, but two discontinued prior to gel application. Blood was collected during both phases at the seven times post-dose. TFV exposure was low in blood plasma for SD and MD; median C_max_ was 4.0 and 3.4 ng/mL, respectively (C≤29 ng/mL). TFV concentrations were high in aspirates and tissue after SD and MD, ranging from 1.2×10^4^ to 9.9×10^6^ ng/mL and 2.1×10^2^ to 1.4×10^6^ ng/mL, respectively, and did not noticeably differ between proximal and distal tissue. TFV diphosphate (TFV-DP), the intracellular active metabolite, was high in ECC, ranging from 7.1×10^3^ to 8.8×10^6^ ng/mL. TFV-DP was detectable in approximately 40% of the tissue samples, ranging from 1.8×10^2^ to 3.5×10^4^ ng/mL. AUC for tissue TFV-DP was two logs higher after MD compared to SD, with no noticeable differences when comparing QD and BID.

**Conclusions:**

Single-dose and multiple-dose TFV gel exposure resulted in high genital tract concentrations for at least 24 hours post-dose with minimal systemic absorption. These results support further study of TFV gel for HIV prevention.

**Trial registration:**

ClinicalTrials.gov NCT00561496

## Introduction

An estimated 25 million people have died from complications from HIV since AIDS was recognized in 1981. Approximately 33 million people were living with HIV at the end of 2009 [1]. To date, the only available strategies for female users for prevention of sexual transmission are condoms and abstinence. New prevention methods such as user-controlled topical microbicides that can be applied to the vagina (or rectum) are urgently needed.

Tenofovir (TFV) is a nucleotide reverse transcriptase inhibitor (NtRTI) that prevents transcription of HIV RNA to DNA when converted to its active form, TFV diphosphate (TFV-DP) [2]. TFV disoproxil fumarate (TDF, or Viread®), an orally bioavailable form of TFV, received marketing approval for the treatment of HIV-1 infection in the United States in 2001. The cumulative worldwide patient exposure to TDF is estimated to be 3.5 million patient-years of treatment. In December 2006, Gilead granted co-exclusive rights to CONRAD and the International Partnership for Microbicides (IPM) to develop, manufacture and distribute TFV gel as a topical microbicide in resource-limited countries.

Multiple nonclinical studies have demonstrated the *in vitro* efficacy of TFV for preventing HIV transmission [3]. TFV gel has also been proven effective in preventing vaginal SHIV transmission in macaques [4]. TFV 1% gel has been found to be safe and well tolerated in women and men [5]–[7]. Subsequent to this current study, a randomized, double-blind, placebo-controlled trial demonstrated that vaginal TFV 1% gel reduced male-to-female HIV transmission by 39% [8]. In that study, women followed a regimen in which dosing could take place up to 12 hours before and up to 12 hours after intercourse, with no more than two doses administered in a 24-hour period. The HIV incidence rates in the TFV gel and placebo arms were 5.6 (confidence interval [CI]: 4.0, 7.7) and 9.1 (CI: 6.9, 11.7) per 100 women-years, respectively (incidence rate ratio = 0.61; *P* = 0.017). Several other safety and effectiveness studies of 1% TFV gel as an HIV prevention strategy are ongoing or in development, including a daily dosing strategy (VOICE: clinicaltrials.gov NCT00705679).

The objectives of the pharmacokinetic (PK) study described here were to assess the local and systemic exposure of extracellular TFV and intracellular TFV-DP after a single dose and after 14 days of once- or twice-daily dosing. To our knowledge, this study was the first to measure TFV and intracellular TFV-DP in human genital tract cells and tissues.

## Methods

The protocol for this trial and supporting CONSORT checklist are available as supporting information; see Checklist S1 and Protocol S1.

### Ethics Statement

This study conformed to the principles of the Declaration of Helsinki. The participants took part voluntarily and signed informed consent forms. The study was reviewed and approved by the PROFAMILA Ethics Committee, Santo Domingo, Dominican Republic; the University of Pittsburgh Institutional Review Board (IRB), Pittsburgh, PA; and the Western IRB, Olympia, WA.

This pharmacokinetic study was performed at three centers: PROFAMILIA in Santo Domingo, Dominican Republic; Magee-Womens Hospital in Pittsburgh, PA; and Advances in Health in Houston, TX. A total of 49 women at these three sites were enrolled to receive a single vaginal dose (4 mL) of 1% TFV gel followed by a 1-month wash-out period and then 2 weeks of either once- or twice-daily dosing. A subset of participants in Pittsburgh who completed the first two phases of the study was asked to participate in a third phase with sample collection at 12 hours after an additional single dose following a wash-out period.

Gels were packaged in labeled single use tubes of 1% TFV gel and were inserted with a single-use applicator (HTI Plastics, Lincoln, NE) administering 4 mL. Two different multiple-dose kits were provided for once-daily (14 tubes and applicators) and twice-daily (27 tubes and applicators) dosing over 14 days.

Healthy female volunteers 18–45 years old at low risk for sexually transmitted infections were considered for enrollment. Eligible volunteers had no clinically significant systemic diseases, had regular menstrual cycles, were not at risk for pregnancy (using Paragard® IUD, an effective barrier method, female sterilization or abstinence, or had a vasectomized partner), agreed to be sexually abstinent for 72 hours prior to, and for the entirety of, the study phases, and provided signed informed consent.

Volunteers were ineligible if they were breastfeeding or within 2 months of their last pregnancy outcome, used any hormonal contraceptives within 30 days of enrollment, used Depo-Provera within 120 days of enrollment, had a history of an abnormal Pap smear that had not been evaluated and treated as indicated, had a recent history of drug or alcohol abuse, or used antiretroviral therapies or drugs on a daily basis that could reduce renal or liver function. Volunteers who tested positive for HIV, HBsAg, *Trichomonas vaginalis, Neisseria gonorrhoeae* or *Chlamydia trachomatis*, or had genital findings suspicious for a sexually transmitted infection or a current vaginal or urinary tract infection were also ineligible along with volunteers who had abnormal laboratory values or had participated in another research study within 30 days of screening. Participants were compensated for expenses incurred and time and effort consistent with the region. This was reviewed and approved by the local IRB based on their assessment that payment would not influence the volunteer's decision to participate in the study.

### Study Procedures

**Figure 1:**
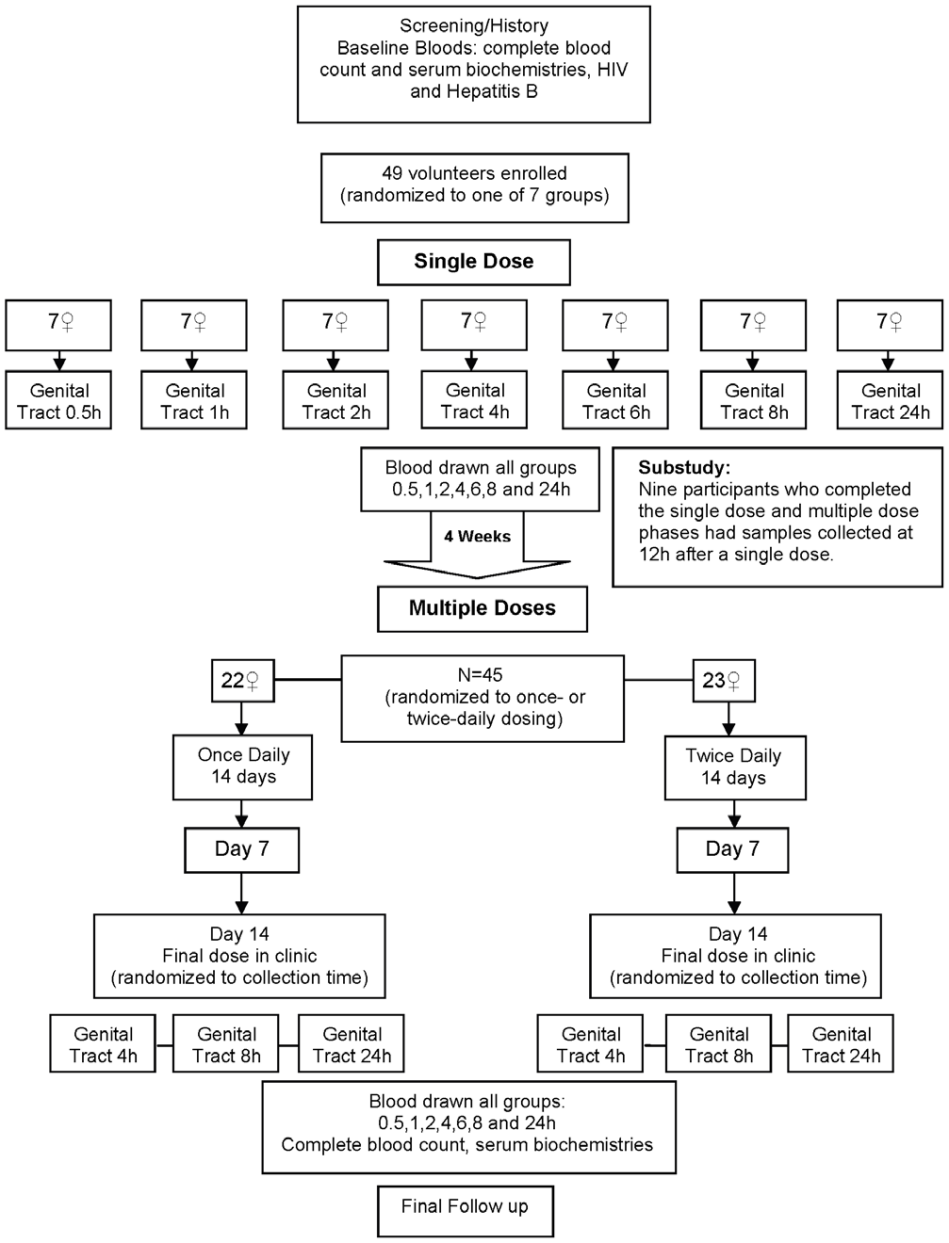
Study procedures.

Each female volunteer participated in six visits over three menstrual cycles, received gynecological examinations and was tested for pregnancy at every visit. Figure 1 details the study procedures. At screening, volunteers were given information about the study and provided written informed consent. Baseline serum biochemistries and complete blood counts were collected, and testing for genital infections was performed. The enrollment visit was scheduled on menstrual cycle day 19–24. Eligible volunteers were enrolled and randomized to collection of endocervical cells (ECC), cervicovaginal aspirate and vaginal biopsies at one of seven time points [0.5, 1, 2, 4, 6, 8, and 24 hour(s)] post-dose for measurement of TFV concentration. A single dose of intravaginal TFV gel was applied at the study site. Blood samples were collected at all seven time points to assess systemic TFV exposure.

Participants returned for a 2-week course of intravaginal TFV gel after at least a 1-month wash-out and after menstrual flow ceased. Participants were randomly assigned to either once-daily (in the morning) or twice-daily (at 12-hour intervals) gel application and received study supplies to use at home. Participants returned for an interim visit after 1 week and a final treatment visit on the 14^th^ day of product use at which time blood samples were obtained for TFV concentrations. Prior to the application of the final study dose at the study site, participants were randomized to collection of genital tract specimens at 4, 8 or 24 hours post-final dose on menstrual cycle day 19–24. As with the single dose, blood samples were collected at all seven time points [0.5, 1, 2, 4, 6, 8, and 24 hour(s)]. A post-treatment safety visit occurred 1–2 weeks later, at which time the participant was discontinued from the study if the examination was normal.

Because of the logistical difficulty of collecting samples at the 12-hour time point, a substudy was performed at the University of Pittsburgh site to enroll up to 10 women who had participated in the main study to have biopsies and blood samples collected 12 hours after an additional single dose.

Twenty-one vaginal tissue samples from the single dose phase were used for an interim analysis that was presented elsewhere and is not reported here.

### Sample Collection

The cervicovaginal fluid pooling in the posterior fornix was aspirated with a 1.5 mL vaginal syringe, expelled into a cryogenic vial, and frozen at −80°C. ECC specimens were collected with two 360° turns of a cytobrush in the endocervix. The brush was placed in a tube containing phosphate-buffered saline (PBS) and agitated to remove cells. The brush was removed, and the tube was spun at 4°C at 400 relative centrifugal force (RCF)×15 minutes. The pellet was resuspended in 1 mL PBS and a 10 µl sample was mixed with 0.4% Trypan Blue and counted for mononuclear cells. The remainder of the sample was spun at 4°C at 400 RCF×15 minutes and the cell pellet was mixed with 1.0 mL of ice cold 70% methanol, vortexed lightly to lyse the cells and stored at −80°C.

The areas of the planned biopsies were gently wiped with a small cotton swab moistened with warm saline followed by a small cotton swab with betadine. Topical benzocaine gel was applied to the areas of the planned biopsies for anesthesia. A medium-Tischler biopsy forceps was used to obtain two 5×3 mm vaginal biopsies; one from the upper one-third (proximal) and one from the lower one-third (distal) of the vagina. Each biopsy was placed in a pre-weighed individual vial and weighed, snap frozen in liquid nitrogen, and stored at −80°C until transport. If adequate hemostasis at a biopsy site was not obtained with pressure, Monsel's solution or silver nitrate was applied.

### Drug Concentration Measurements

All methods were validated as mandated by the industry guidance set by the US Department of Health and Human Services (US DHHS), Food and Drug Administration (FDA), Center for Drug Evaluation and Research (CDER).

#### Blood Plasma TFV Concentrations

Analysis of TFV was performed by extracting drug species and internal standard from 200 µL standards, quality control samples, and participant samples using protein precipitation followed by a solid phase extraction (SPE) procedure. A calibration curve for TFV ranged from 0.5–200 ng/mL and utilized adefovir as the internal standard. Across three quality control concentrations, intra-day accuracy and precision was >99% and <12%, respectively. Inter-day accuracy and precision was >90% and <13%, respectively. The lower limit of quantification (LLOQ) for TFV was 0.5 ng/mL. Detectable values below the limit of quantification (BLQ) were imputed to be one-half of the LLOQ (0.25 ng/mL) and values below the level of detection (BLD) were set to 0 ng/mL.

#### Cervical Vaginal Fluid (CVF) Concentrations

Analysis of TFV in CVF was performed by extracting drug species and internal standard from 50 µL standards, quality control samples, and participant samples using protein precipitation with acetonitrile containing ^13^C-labelled TFV internal standard. A calibration curve for TFV ranged from 2–1,000 ng/mL. Intra-day accuracy and precision was >93% and <8%. Inter-day accuracy and precision was >97% and <5%.

#### TFV and TFV-DP Measurement in Vaginal Tissue

A reverse-phase high performance liquid chromatography (RP-HPLC) method employing tandem mass spectrometry detection (LC-MS/MS) for the quantification of TFV and TFV-DP in human vaginal tissue was performed by extracting drug species from standards, quality control samples and participant samples using tissue homogenization and a protein precipitation procedure. An internal standard working solution of ^13^C-labelled TFV and lamivudine triphosphate was used for the analysis of TFV and TFV-DP, respectively. A calibration curve with a range of 2–2,000 ng/mL for TFV, and 10–10,000 ng/mL for TFV-DP was prepared from separate stock solutions. The LLOQ was 2 and 10 ng/mL for TFV and TFV-DP, respectively. The minimum tissue sample weight required for this assay was 20 mg. Intra-day accuracy and precision for TFV was >90% and <10%, respectively; for TFV-DP it was >94% and <9%, respectively. Inter-day accuracy and precision for TFV was >93% and <5%, respectively: for TFV-DP it was >98% and <6% respectively. No values at BLQ were imputed for the tissue data. Concentrations by tissue weight (ng/g) were converted to volume (ng/mL) using a vaginal tissue density of 1.01 g/mL. TFV-DP was also calculated as fmol/0.2 µL based on the calculation that 0.2 µL is the approximate volume equivalent of 10^6^ mononuclear cells [9].

#### Endocervical Cells TFV-DP

Measurements of intracellular TFV-DP concentrations in endocervical cells obtained by cytobrush were performed as previously described in peripheral blood mononuclear cells (PBMCs) [10], [11].

### Randomization

A random permuted blocks method was used to generate three random allocation sequences by FHI. The first sequence was used to randomize women to one of seven biopsy time points following initial dosing of the gel, stratified by site. The second sequence was used to randomize women to either once- or twice-daily dosing in the 14-day follow-up period, stratified by site. The third sequence was used to randomize women to one of the three concluding biopsy time points, stratified by site and dosing arm within site. In order to conceal the allocation procedure, random assignments were contained within sequentially numbered, sealed opaque envelopes which were maintained in a secure location at the sites and opened by study staff at the time of randomization. The random sequences were created using SAS (SAS Institute, Cary, NC).

### Statistical Analysis

Study objectives were evaluated among participants enrolled and randomized and who provided follow-up data. The primary outcomes were local and systemic drug exposure of TFV after a single dose and after 2 weeks of once- or twice-daily dosing. The chosen sample size (49 women) was not based on formal power considerations, but was deemed sufficient to characterize relevant pharmacokinetic profiles, assuming groups of at least seven at each time point and similar standard deviations as observed in previous plasma data [5].

Blood plasma C_max_, T_max_ and area under curve (AUC)_0–24 h_ (calculated using the linear trapezoidal method) were estimated by noncompartmental analysis using WinNonlin Version 5.2 (Pharsight Corporation, Sunnyvale, CA). Because participants only provided vaginal tissue specimens at a single time point in each phase of the study, tissue TFV and TFV-DP C_max_ and T_max_ values were estimated based on the median response at each time point using SAS Version 9.1. For these sparse tissue data, the median value at each time point was estimated and used to calculate C_max_ and T_max_. The composite of these median values was used to calculate AUC_0–24 h_. Cervicovaginal fluid and cytobrush specimen concentrations were summarized similarly.

## Results

**Figure 2:**
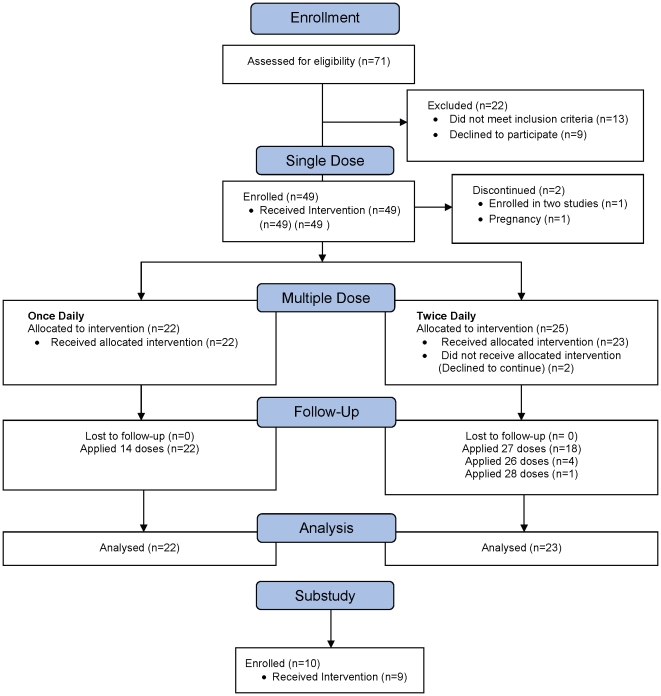
Participant flow diagram.

Recruitment for this study occurred from March 27, 2007 to January 9, 2008. The last study visit was on April 11, 2008. A total of 49 women met eligibility criteria and were enrolled (Figure 2). All 49 women received a single dose of TFV gel in the clinic, but two discontinued, one before and one after sample collection. Therefore, 47 participants proceeded to the MD phase and were randomized to once (QD) or twice daily (BID) dosing. Two of these 47 participants discontinued prior to gel application. A total of 975 doses were applied. Nine participants completed the 12-hour substudy and used a total of nine doses.

Demographic characteristics for all enrolled participants are shown in Table 1. Participants from the Dominican Republic differed from the other two sites in that they more frequently identified themselves as Hispanic and were less educated, and all had a previous pregnancy and used female sterilization as their birth control method (data not shown). Participants in Pittsburgh had the highest mean Body Mass Index (34), those in Houston the second highest (30), and those in the Dominican Republic the lowest (28) (data not shown).

**Table 1:** Participant characteristics.

	Female (N = 49)	
	N	%
**Age (years)**		
Mean ± SD	33.8 (6.5)	
Range	20–45	
**Ethnicity**		
Hispanic	26	53
Not Hispanic	23	47
**Race**		
Black or African American	17	35
White	10	20
More than one race	22	45
**Education (years)**		
Mean ± SD	11.0 (3.9)	
**Body Mass Index**		
Mean ± SD	30.3 (6.9)	
Range	18–50	
**Ever pregnant**		
Yes	41	84

No deaths or Serious Adverse Events were reported. Of 192 Adverse Events (AEs) reported from 42 of 48 participants, 190 were reported during MD. The most frequently reported AEs occurred within the following System Organ Class (SOC): Reproductive System and Breast Disorder (27/48 participants, 56.3%), Nervous System Disorders (18/48 participants, 37.5%), Gastrointestinal Disorders (18/48 participants, 37.5%), and Investigations (13/48 participants, 27.1%).

Of the 192 reported AEs, 59 of these were designated as possibly (n = 24), probably (n = 11) or definitely (n = 24) related to the study gel or applicator and were experienced in 17 participants. Of the 59 designated as at least possibly related, 53 occurred in the Reproductive System and Breast Disorder SOC, three occurred in the Gastrointestinal Disorder SOC and three occurred in the Subcutaneous Tissue SOC.

A total of 8 severe AEs were reported by 6 women including headaches (n = 4), vaginal hemorrhage (n = 2), subcutaneous abscess (n = 1) and dysuria (n = 1), which were judged unrelated to study product by site investigators.

A total of 26 moderate AEs were reported by 16 women. These moderate AEs included dysmenorrhea (n = 4), headache (n = 4), menorrhagia (n = 1), genital discomfort/pain (n = 2), back pain (n = 2), vaginal odor (n = 1), hemoglobin decreased (n = 1), pruritus (n = 1), nasopharyngitis (n = 1), myalgia (n = 1), tonsillitis (n = 1), toothache (n = 1), abdominal pain (n = 1), muscle spasm (n = 1), arthralgia (n = 1), abdominal distention (n = 1), dysuria (n = 1) and nausea (n = 1). Only three of these were judged to be related to study product: vaginal odor, pruritus and vulvovaginal discomfort.

Table 2 shows the Reproductive System AEs reported in this study. A total of 27 participants reported a total of 88 Reproductive System AEs. Of these, 5 participants reported 8 AEs of moderate severity [dysmenorrhea (4), menorrhagia (1), vaginal odor (1), pruritus (1), vulvovaginal discomfort (1)], of which the latter 3 were judged to be at least possibly related. Two participants experienced bleeding from a biopsy site that required immediate evaluation and treatment, which were judged as severe but unrelated to study product. Of the remaining 78 Reproductive System mild AEs, 50 were judged to be at least possibly product related.

**Table 2:** Reproductive system adverse events.

	Tenofovir Gel (N = 48)	
	# of events	# of women (%)
Metrorrhagia/abnormal vaginal bleeding	16	12 (25)
Pruritus	28	9 (19)
Discharge	8	7 (15)
Vulvovaginal Discomfort	8	7 (15)
Dysmenorrhea	8	4 (8)
Vaginal Burning	4	4 (8)
Vaginal Odor	6	3 (6)
Genital Fissure	3	2 (4)
Vaginal Candidiasis	2	2 (4)
Bacterial Vaginosis	2	2 (4)
Menorrhagia	1	1 (2)
Vaginal Pain	2	1 (2)
Total	88	27 (56.3%)

One participant became pregnant 3 weeks following exposure to the single dose and was discontinued prior to the second phase. The pregnancy ended in miscarriage, which was judged to be unrelated to the study drug as it was remote from exposure. There were no clinically significant changes in complete blood counts and serum biochemistries between screening and the final visit (data not shown).

### Pharmacokinetic Parameters

#### TFV Plasma Measurement Data

The concentration of TFV measured in all blood plasma samples ranged from below the level of detection to 29.1 ng/mL. The median (interquartile range [IQR]) estimate for C_max_ in the single-dose phase was 4.0 ng/mL (1.5–9.1) (Table 3). The median (IQR) estimate for C_max_ at the end of the 2-week phase was 3.4 ng/mL (2.4–6.1) when pooled across dose groups (Table 4). The estimated C_max_ was lower for the once-daily group than for the twice-daily group: 2.5 ng/mL (1.8–3.4) compared to 5.3 ng/mL (3.2–10.6) (Table S1). The median AUC_0–24 h_ was similar for the single- and multiple-dose regimens: 36.4 ng * hr/mL (14.0–73.3) (Table 3) and 37.2 ng * hr/mL (24.6–62.6) (Table 4), respectively. There were no noticeable differences in exposure between SD and MD.

**Table 3:** Median single-dose (SD) PK parameters (all data points collected during the study).

Analyte, Matrix	N	N(>BLQ )	Samples(>BLQ/Total #)	C_max_ (ng/mL)	T_max_ (hr)	AUC_0–24 h_ (hr*ng/mL)	C_24 h_ (ng/mL)
				SD	SD	SD	SD
TFV BP (IQR)	48	48	313/392 (80%)	4.0 (1.5–9.1)	4 (2–6)	36.4 (14.0–73.3)	0.3 (0.3–0.5)
TFV CVF	45	45	52/52 (100%)	1.9×10^6^	4	20.7×10^6^	0.1×10^6^
TFV Vaginal Tissue	33	33^3^	72/72 (100%)	2.2×10^5^	2	64.0×10^4^	0.7×10^4^
TFV-DP ECC	48	39	42/57 (74%)	7.5×10^5^	4	47.4×10^5^	0.8×10^5^
TFV-DP Vaginal Tissue	33	15^4^	36/72 (50%)	3.8×10^3^	1	12.4×10^3^	n/a
TFV-DP ECC^1^	48	39	42/57 (74%)	33.6×10^4^	4	212.2×10^4^	3.5×10^4^
TFV-DP Vaginal Tissue^2^	33	15	36/72 (50%)	1.7×10^3^	1	5.6×10^3^	n/a

1 C_max_ and C_24 h_ in fmol/10^6^ cells; AUC_0–24 h_ in hr * fmol/10^6^ cells.

2 C_max_ and C_24 h_ in fmol/0.2 µL; AUC _0–24 h_ in hr * fmol/0.2 µL.

3 Thirty-six data points contributed to the TFV tissue analysis.

4 Fifteen data points out of a potential 36 contributed to the TFV-DP tissue analysis.

**Table 4:** Median multi-dose (MD) PK parameters (all data points collected during the study).

Analyte, Matrix	N	N(>BLQ )	Samples (>BLQ/Total #)	C_max_ (ng/mL)	T_max_ (hr)	AUC_0–24 h_ (hr*ng/mL)	C_24 h_ (ng/mL)
				MD	MD	MD	MD
TFV BP (IQR)	45	45	345/360 (96%)	3.4 (2.4–6.1)	4 (2–6)	37.2 (24.6–62.6)	0.3 (0.3–0.6)
TFV CVF	45	36	36/36 (100%)	1.4×10^6^	4	18.2×10^6^	0.6×10^6^
TFV Vaginal Tissue	45	45	89/89 (100%)	2.7×10^4^	4	34.5×10^4^	0.6×10^4^
TFV-DP ECC	45	35	35/45 (78%)	3.6×10^5^	8	44.5×10^5^	0.5×10^5^
TFV-DP Vaginal Tissue	45	16	24/89 (27%)	3.1×10^3^	8	43.4×10^3^	1.3×10^3^
TFV-DP ECC^1^	45	35	35/45 (78%)	16.2×10^4^	8	205.1×10^4^	3.1×10^4^
TFV-DP Vaginal Tissue^2^	45	16	24/89 (27%)	1.3×10^3^	8	19.4×10^3^	0.6×10^3^

1 C_max_ and C_24 h_ in fmol/10^6^ cells; AUC_0–24 h_ in hr * fmol/10^6^ cells.

2 C_max_ and C_24 h_ in fmol/0.2 µL; AUC_0–24 h_ in hr * fmol/0.2 µL.

#### TFV Cervicovaginal Fluid Concentration

Pooling all samples from 1 to 24 hours, the concentration of TFV in direct aspiration of the cervicovaginal fluid ranged from 1.2×10^4^ to 9.9×10^6^ ng/mL.

The median C_max_ after a single dose was 1.9×10^6^ ng/mL (Table 3). The range was 1.2×10^4^−9.9×10^6^.

The median C_max_ at the end of the 2-week phase was 1.4×10^6^ ng/mL (Table 4). The range was 8.4×10^4^−5.8×10^6^. The median C_max_ was similar for the once (QD) and twice daily (BID) groups: 1.8×10^6^ ng/mL compared to 1.4×10^6^ ng/mL, respectively (Table S1) and ranges of 8.4×10^4^−2.4×10^6^ and 1.5×10^5^−5.8×10^6^, respectively. The estimated AUC for the MD QD and BID groups was 26.2×10^6^ and 18.4×10^6^, respectively (Table S1).

#### TFV Vaginal Tissue

The data are presented in Table 3 which shows all data from SD, Table 4 which shows all data from MD and Table S1 which compares SD and MD tissue concentrations at equivalent time points for genital data (4, 8 and 24 hours).

#### TFV Vaginal Tissue [ng/mL]

There were 24 women who contributed one time point [0.5, 1, 2, 4, 6, 8, or 24 hr(s)], 6 women who contributed only to the 12 h time point and 3 who contributed two time points ([0.5, 1, 2, 4, 6, 8, or 24 hr(s)] and 12 h) in the SD phase and 44 from the MD phase who contributed tissue samples for TFV concentrations; all had detectable values. One participant in the MD phase had a missing tissue sample, so did not contribute to the analysis. To determine whether systematic differences in TFV exposure could be detected, the ratio of distal to proximal TFV concentration was calculated. The geometric mean of this ratio did not differ significantly from 1 (0.91; 90% CI 0.78, 1.06). The values from the proximal and distal vaginal specimens were averaged together for subsequent tissue analysis, if both were available. Therefore, 36 data points contributed to the SD analysis and 44 contributed to the MD analysis.

Pooling all samples from 1 to 24 hours, the concentration of TFV in the vaginal biopsies ranged from 2.1×10^2^ to 1.4×10^6^ ng/mL.

The median C_max_ was 2.2×10^5^ ng/mL after a single dose when comparing all data points (Table 3) and 1.2×10^4^ estimated from 4, 8 and 24 hour samples (Table S1). The composite median AUC for SD was 64.0×10^4^ and the C_24 h_ was 0.7×10^4^ (Table 3).

The C_max_ at the end of the 2-week phase was somewhat lower for the once-daily than for the twice-daily group (2.9×10^4^ ng/mL and 5.2×10^4^ ng/mL, respectively) (Table S1).

#### TFV-DP Vaginal Tissue [ng/mL]

Quantifiable TFV-DP levels in vaginal tissue were detected in 15 out of 36 (42%) data points that contributed to the SD phase analysis and 16 out of 44 (36%) in the MD phase analysis. Detectable concentrations in at least one sample occurred in five out of 21 participants from the Dominican Republic, 11 out of 14 from Houston and 8 out of 14 from Pittsburgh.

**Figure 3:**
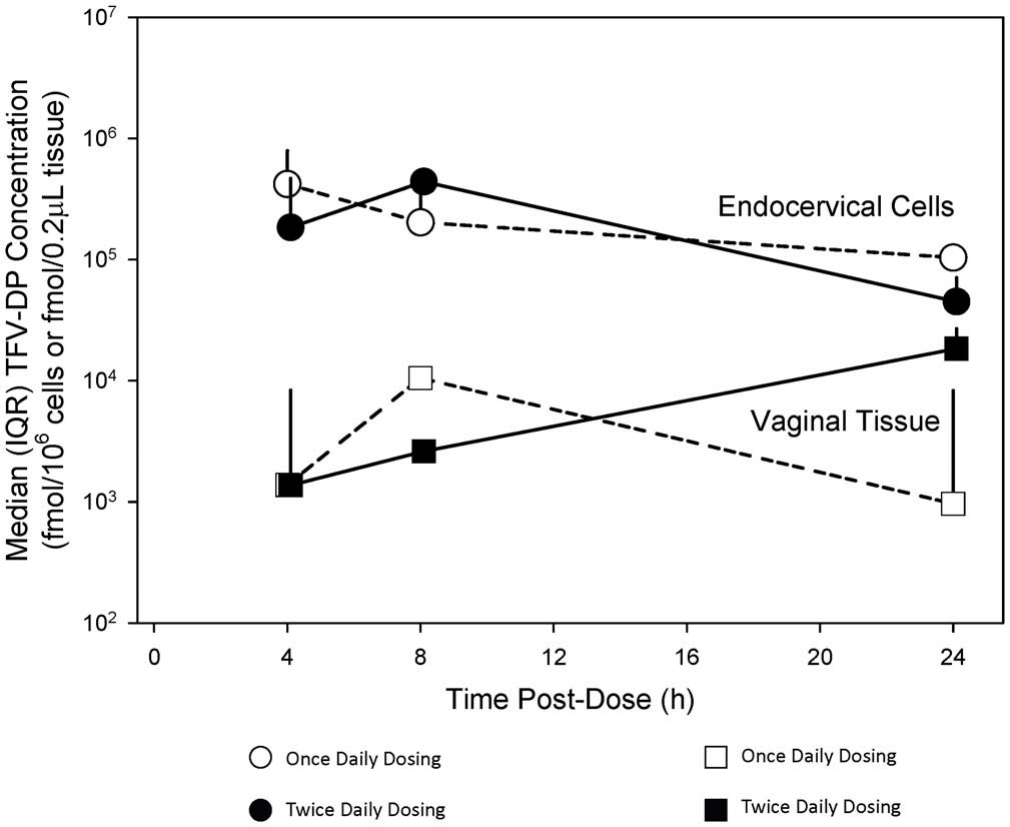
Tenofovir-diphosphate (TFV-DP) median concentrations in endocervical cells (ECC) and vaginal tissue after once- or twice-daily dosing.

In those with detectable values, the vaginal biopsy concentrations of TFV-DP ranged from 1.8×10^2^ to 3.5×10^4^ ng/mL. The median C_max_ after a single dose was 3.8×10^3^ ng/mL (1.7×10^3^ fmol/0.2 µL) (Table 3), and at the end of the 2-week phase was 3.1×10^3^ ng/mL (1.3×10^3^ fmol/0.2 µL) (Table 4) in those with detectable values. These latter C_max_ median values were slightly different for the once-daily and the twice-daily groups (1.1×10^4^ ng/mL [4.7×10^3^ fmol/0.2 µL] compared to 0.3×10^4^ ng/mL [1.2×10^3^ fmol/0.2 µL], respectively) (Table S1) with once-daily concentrations higher at 8 hours and lower at 24 hours (Figure 3).

#### Intracellular TFV-DP in Endocervical Cells Collected by Cytobrush

Quantifiable TFV-DP concentrations were detectable in endocervical samples. After a single dose, the ECC concentration of TFV-DP ranged from 7.1×10^3^ to 8.8×10^6^ ng/mL and the median C_max_ was 7.5×10^5^ ng/mL (3.4×10^5^ fmol/10^6^ cells) (Table 3). The estimated C_max_ at the end of the 2-week phase was similar for the once-daily and the twice-daily groups (4.2×10^5^ ng/mL compared to 4.4×10^5^ ng/mL, respectively) (Table S1, Figure 3).

There was no evidence that the act of taking biopsy samples influenced systemic blood TFV concentrations at later time points, since concentrations were similar whether biopsies were taken prior to or after the blood draw (data not shown).

**Figure 4:**
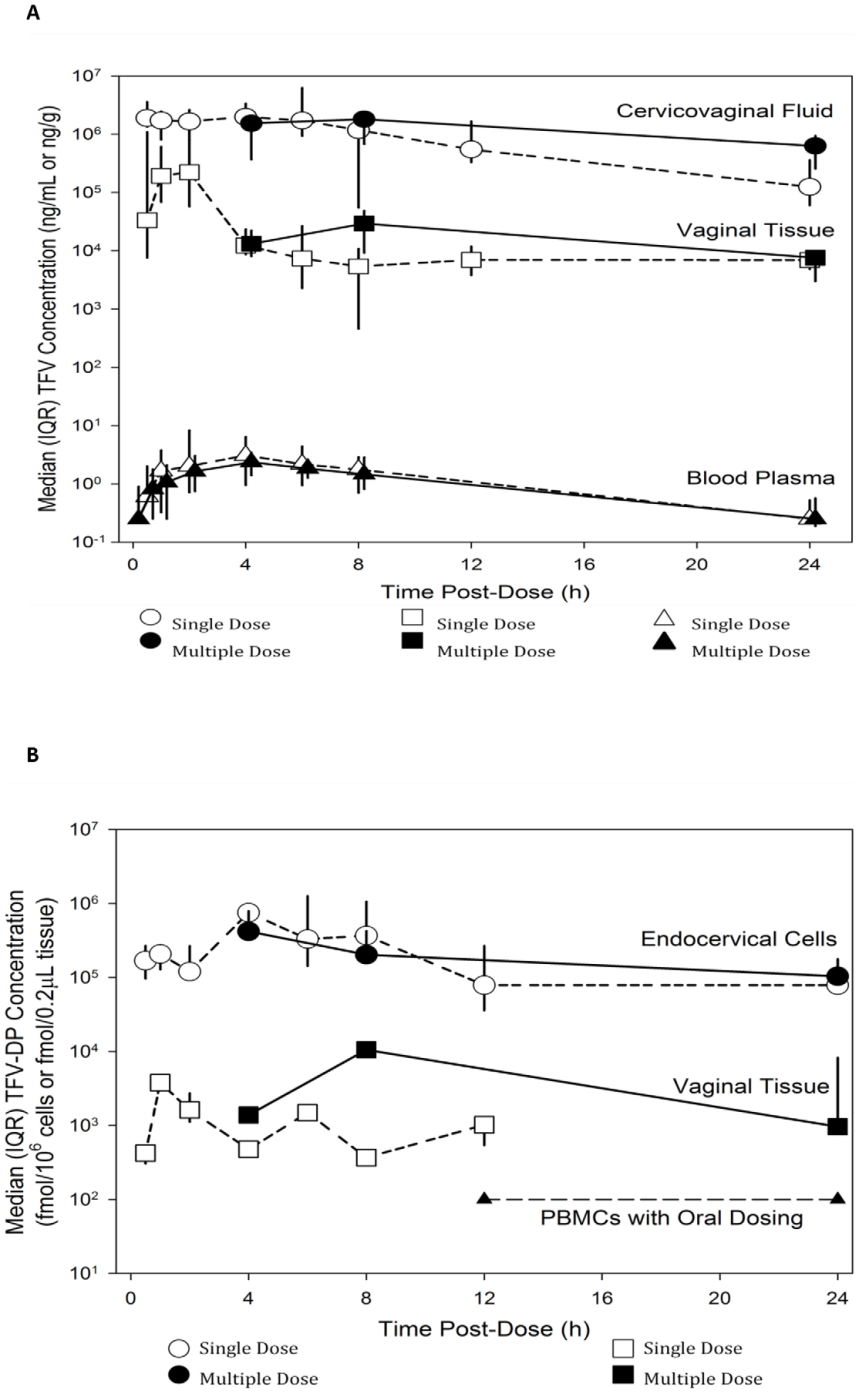
Tenofovir (TFV) and tenofovir-diphosphate (TFV-DP) median concentrations in blood, cervicovaginal fluid and vaginal tissue following single and multiple doses. For vaginal tissue measurements, 15/36 (42%) single dose data points and 16/45 (38%) multiple dose data points had TFV-DP concentrations above the limit of detection (4.5 fmol/0.2 µL). Figure 4B includes only data from subjects with detectable concentrations. PBMC data from Hawkins et al. [10].

Table S1 displays the median PK parameter estimates in the various anatomical compartments comparing the 4, 8 and 24 hour time points for single-dose and multiple-dose users. The AUC for vaginal tissue TFV-DP was two logs higher in multiple-dose compared to single-dose users but there were no noticeable differences between QD and BID. In women with detectable blood plasma concentrations, the median was close to zero at 24 hours. Vaginal tissue and endocervical cell TFV and TFV-DP concentrations remained high at 24 hours. The changes in TFV and TFV-DP concentrations in multiple compartments over the 24 hours following dose administration are shown in Figure 4. Figure 4B represents only those values with measurable concentrations of TFV-DP.

## Discussion

TFV gel is under development as a microbicide and is being evaluated in clinical trials with pericoital and daily regimens. Due to its long intracellular half-life, TFV disoproxil fumarate can be taken once daily for oral treatment with tolerable side effects. Extracellular and intracellular measurements of this compound can be utilized to better assess required drug concentrations for safety, efficacy, and the prevention of resistance through pharmacokinetic studies. Measurements of extracellular TFV have been performed in a number of matrices after oral exposure and in blood after vaginal exposure [5]. Measurements of intracellular concentrations of the active metabolite TFV-DP have been performed in PBMCs [10]. We evaluated TFV concentrations in blood plasma, vaginal fluid and vaginal tissue with single-dose administration as well as use over 14 consecutive days. However, this is the first study to measure female genital tissue and cell concentrations of TFV-DP, the active metabolite of TFV gel. With topical microbicides, since the gel is administered vaginally, it is important to measure intracellular concentrations in addition to extracellular concentrations to be certain that TFV has been absorbed.

With comparable sampling times (4, 8 and 24 hours), there was a trend toward higher concentrations of both TFV and TFV-DP in the vaginal tissue after multiple doses compared to a single dose, and the AUC for vaginal tissue TFV-DP was two logs higher in multiple-dose compared to single-dose users. Conversely, lower concentrations of TFV-DP were observed in the endocervical cells after multiple doses compared to a single dose. These and other inconsistent patterns might be a result of variability within this somewhat small sample and sparse data, making it hard to come to definitive conclusions. However, the formation of more TFV-DP after multiple doses is logical and would indicate that increased exposure time and exposure to more TFV allows for greater phosphorylation to occur within tissue mononuclear cells. Of importance for either daily or pericoital dosing is the fact that TFV tissue concentrations after both single and multiple doses were above the range of *in vitro* anti-HIV-1 EC_50_s (0.4–8.5 µM) and remained so for at least 24 hours post-dose [12], [13]. Tenofovir tissue concentrations were also above those required to inhibit K65R-bearing viruses, a mutation that confers resistance to the drug (EC_50_ = 25.3 µM) [14]. It is noteworthy, however, that complete inhibition of HIV-1 replication in cervicovaginal tissues may require tenofovir concentrations close to the median levels achieved in mucosal tissues after gel administration. Given the range of concentrations obtained after single and multiple doses of tenofovir gel, it is conceivable that some women might not be fully protected. Furthermore, environmental factors such as vaginal infections or inflammation, which facilitate HIV infection, as well as intercourse itself, may modify the required effective dose [15].

With multiple dosing, plasma TFV concentrations were about twice as high after twice-daily compared to once-daily dosing. There were no noticeable differences in TFV concentrations between once- and twice-daily dosing in the cervicovaginal aspirate. While the median concentration of TFV in vaginal tissue was somewhat lower for the once-daily compared to the twice-daily group, there were no noticeable increases in the concentrations of the active metabolite (TFV-DP) in vaginal tissue or endocervical cells when comparing once-daily to twice-daily use.

As expected with intravaginal dosing, the systemic exposure after a single application was lower compared to that of oral dosing. The AUC in plasma was 36.4 compared to approximately 10^3^ ng * h/mL after an oral dose of TFV disoproxil fumarate as reported by Hoetelmans, et al. [16]. In comparison, the concentrations of TFV-DP in the genital tract were high at exposures similar to, or up to 2 logs greater than what is seen in PBMCs after oral administration [10], as shown in Figure 4B. Ideally, a topical microbicide should have exactly these properties of low systemic exposure and high local concentration in order to minimize side effects and the development of resistance. The low tenofovir plasma concentrations (1–30 nM) attained after intravaginal gel administration are below its average *in vitro* EC_50_ (∼1 µM), making the possibility of inducing resistant virus unlikely. This assumption is further supported by the high genetic barrier to resistance demonstrated by tenofovir, both *in vitro* and *in vivo* [13], [17].

Although high concentrations of intracellular TFV-DP were detected in a subset of participants for at least 24 hours post-dose, the window of protection afforded by vaginal application and the target concentration of protection in tissue remains unknown. However, concentrations in the cervicovaginal fluid were greater than 10^4^ ng/ml which has been suggested to be the required threshold to prevent HIV infection based on Phase IIb data [18]. Without further study there is no way to know the ideal dosing interval for pericoital application, as shorter or longer intervals may be clinically effective.

Although our study is critical to the understanding of how TFV gel is absorbed and metabolized in genital tissue, several limitations must be acknowledged. Each participant only contributed to one time point for the vaginal biopsy due to feasibility issues and the concern that conducting multiple biopsies in individuals might affect plasma PK results. Because it did not appear that biopsies led to increased systemic absorption, future studies may consider conducting multiple biopsies in individuals to obtain more complete PK profiles. Another limitation is that TFV-DP was detectable in about 40% of the vaginal tissue biopsy samples, and was detected less frequently at the Dominican Republic site. The inability to detect TFV-DP in specimens was likely due to the instability of the metabolite in tissue samples. This issue was identified during the development of the analytical method for TFV-DP whereby manipulation of the tissue quickly degraded the TFV-DP to TFV. This phenomenon was verified by two other laboratories, and has been subsequently overcome by adding solvents such as methanol to inactivate the enzymes responsible for this degradation. Finally, there is some evidence that epithelial cells are able to phosphorylate TFV [19] and so the tissue concentrations may be a reflection of phosphorylation by both immune and epithelial cell populations. If so, the concentrations of TFV-DP reported in the cytobrush samples might be artificially high because they were calculated from counts of mononuclear cells in the sample, when a proportion of epithelial cells were clearly present and potentially contributing to the concentration measured. Similarly, the concentrations of TFV-DP in the vaginal tissue might be falsely low, reflecting a dilution of the sparse mononuclear cell population (∼50,000 CD4+ cells/biopsy) with epithelial cell concentrations. Epithelial cells may produce TFV-DP at a different rate than immune cells, confounding the interpretation of pharmacokinetic profiles. Nonetheless, the concentrations of TFV-DP in genital tissue after vaginal dosing appear to be higher than intracellular concentrations obtained in PBMCs [10] and extracellular concentrations in vaginal fluid [20] after oral dosing.

Our results show that single-dose and multiple-dose TFV gel exposure leads to high genital tract concentrations for at least 24 hours post-dose and support further study of pericoital and once-daily dosing of TFV gel as potential strategies for the prevention of HIV transmission. The accurate determination of the phosphorylated form of TFV in genital tissues adds to our understanding of transmission pharmacokinetic-pharmacodynamic relationships. These data also offer an understanding of the genital tissue concentrations after single and multiple dosing that may correlate with the reduction of HIV transmission that was demonstrated in the recent Phase IIb study [8].

## Supporting Information

Table S1 The median PK parameters for single and multiple dosing by once- or twice-daily dosing (equivalent sampling time points only for genital compartments).
(DOCX)

Protocol S1 Trial Protocol
(PDF)

Checklist S1 CONSORT Checklist
(DOC)
