# Supplementary material for: A Multi-Compartment, Single and Multiple Dose Pharmacokinetic Study of the Vaginal Candidate Microbicide 1% Tenofovir Gel
Source: PLoS One. 2011 Oct 19;6(10):e25974. doi: 10.1371/journal.pone.0025974 (PMC3198383; doi:10.1371/journal.pone.0025974)
Supplement: Table S1 — The median PK parameters for single and multiple dosing by once- or twice-daily dosing (equivalent sampling time points only for genital compartments). (DOCX) [file pone.0025974.s001.docx]

**Table S1. Median PK parameters for single and multiple dosing by once- or twice-daily dosing (equivalent sampling time points only for genital compartments).**

| **Analyte, Matrix** | **C_max_ (ng/mL)** | | | **T_max_ (hr)** | | | **AUC_0-24h_ (hr*ng/mL)** | | | **C_24h_ (ng/mL)** | | |
| --- | --- | --- | --- | --- | --- | --- | --- | --- | --- | --- | --- | --- |
|  | **SD** | **MD** | | **SD** | **MD** | | **SD** | **MD** | | **SD** | **MD** | |
|  |  | **QD** | **BID** |  | **QD** | **BID** |  | **QD** | **BID** |  | **QD** | **BID** |
| TFV BP (IQR) | 4.0 (1.5-9.1) | 2.5 (1.8-3.4) | 5.3(3.2-10.6) | 4 (2-6) | 4 (2-4) | 4 (1-6) | 36.4 (13.5-69.6) | 26.3 (17.3-38.4) | 51.0(34.3-106.9) | 0.3 (0.3-0.5) | 0.3 (0.3-0.3) | 0.5 (0.3-0.9) |
| TFV CVF^1^ | 1.9 x 10^6^ | 1.8 x 10^6^ | 1.4 x 10^6^ | 4 | 8 | 4 | 18.6 x 10^6^ | 26.2 x 10^6^ | 18.4 x 10^6^ | 0.1 x 10^6^ | 0.6 x 10^6^ | 0.7 x 10^6^ |
| TFV Vaginal Tissue^1^ | 1.2 x 10^4^ | 2.9 x 10^4^ | 5.2 x 10^4^ | 4 | 8 | 4 | 13.3 x 10^4^ | 38.0 x 10^4^ | 34.2 x 10^4^ | 0.7 x 10^4^ | 0.8 x 10^4^ | 0.5 x 10^4^ |
| TFV-DP ECC^1^ | 7.5 x 10^5^ | 4.2 x 10^5^ | 4.4 x 10^5^ | 4 | 4 | 8 | 58.1 x 10^5^ | 37.0 x 10^5^ | 51.3 x 10^5^ | 0.8 x 10^5^ | 1.0 x 10^5^ | 0.5 x 10^5^ |
| TFV-DP Vaginal Tissue^1^ | 0.5 x 10^3^ | 1.1 x 10^4^ | 0.3 x 10^4^ | 4 | 8 | 8 | 1.7 x 10^3^ | 11.5 x 10^4^ | 17.6 x 10^4^ | n/a | 0.1 x 10^4^ | 1.8 x 10^3^ |
| TFV-DP ECC^1,2^ | 33.6 x 10^4^ | 18.8 x 10^4^ | 19.7 x 10^4^ | 4 | 4 | 8 | 259.9 x 10^4^ | 165.4 x 10^4^ | 229.4 x 10^4^ | 3.5 x 10^4^ | 4.6 x 10^4^ | 2.0 x 10^4^ |
| TFV-DP Vaginal Tissue^1,3^ | 0.2 x 10^3^ | 4.7 x 10^3^ | 1.2 x 10^3^ | 4 | 8 | 8 | 0.7 x 10^3^ | 51.8 x 10^3^ | 78.7 x 10^3^ | n/a | 0.4 x 10^3^ | 8.2 x 10^3^ |
| ^1^Estimated from 4, 8 & 24 hour samples | | | | | | | | | | | | |
| ^2^C_max_ and C_24h_ in fmol/10^6^ cells; AUC_0-24h_ in hr * fmol/10^6^ cells | | | | | | | | | | | | |
| ^3^C_max_ and C_24h_ in fmol/0.2 µL; AUC_0-24h_ in hr * fmol/0.2 µL | | | | | | | | | | | | |
