# Supplementary material for: A Multi-Compartment, Single and Multiple Dose Pharmacokinetic Study of the Vaginal Candidate Microbicide 1% Tenofovir Gel
Source: PLoS One. 2011 Oct 19;6(10):e25974. doi: 10.1371/journal.pone.0025974 (PMC3198383; doi:10.1371/journal.pone.0025974)
Supplement: Protocol S1 — Trial Protocol (PDF) [file pone.0025974.s002.pdf]

CONRAD

Single Dose and 14-Day Once or Twice-daily Pharmacokinetic Study of the Vaginal Microbicide Agent 1% Tenofovir Gel

FINAL PROTOCOL # A04-095
Version 4.0

June 18, 2007

This document may contain confidential information. It is understood that persons to whom this information is disclosed will not disclose it further without permission from CONRAD, unless such information is published or otherwise becomes public knowledge.

I, the Principal Investigator, agree to conduct this study in full accordance with the provisions of this protocol and will comply with all requirements regarding the obligations of clinical investigators as fully outlined in 21 CFR Part 56 and in the Statement of Investigator (1572), which I have also signed. I agree to maintain all study documentation until CONRAD consents to disposal of files in writing. I also agree to publish or present data only upon review and approval by CONRAD.

I have read and understand the information in the Investigator's Brochure, including the potential risks and side effects of the product under investigation, and will ensure that all associates, colleagues, and employees assisting in the conduct of the study are informed about the obligations incurred by their contribution to the study.

______________________________ ________________________
Principal Investigator Date

Prepared by:

CONRAD
1611 North Kent Street, Suite 806
Arlington, VA 22209 USA

Tel: 703-524-4744
Fax: 703-524-4770

Single Dose and 14-Day Once or Twice-daily Pharmacokinetic Study of the Vaginal Microbicide Agent 1% Tenofovir Gel

Protocol Number: A04-095
Study Summary

Purpose:
To assess local and systemic absorption and concentration of 1% tenofovir vaginal gel after a single-dose and after two weeks of once or twice-daily dosing in healthy sexually abstinent women.

Objectives:
- To assess the local absorption and concentration of tenofovir after a single dose and after two weeks of once or twice-daily dosing.
- To assess the systemic absorption of tenofovir after a single dose and after two weeks of once or twice-daily dosing.

Design:
This is a multi-center, pharmacokinetic study involving a single-dose phase, a wash out phase and a two-week once or twice-daily dosing phase for each volunteer.

Study Population:
Forty-nine healthy reproductive age women with regular menstrual cycles.

Treatment Regimen:
In the single-dose phase, each volunteer will apply the single dose in the clinic and ambulate for approximately 15 minutes to distribute the gel, after which she may ambulate as desired. Participants will be randomized to have cervicovaginal samples and biopsies collected at one of seven time-points [0.5, 1, 2, 4, 6, 8, and 24 hour(s)] after the single-dose. Blood samples will be drawn at 0.5, 1, 2, 4, 6, 8, and 24 hour(s) after the single-dose, as clinically feasible.

An interim analysis of approximately 21 participants (ideally 7 at each site) measuring tenofovir in plasma, vaginal biopsies, and cervicovaginal fluid following the initial dose will be performed.

In the two-week phase, the study supplies will be distributed and the participants will be randomized to apply each dose either once or twice-daily for two weeks. At the one week follow–up visit a blood sample will be drawn prior to the morning dose to obtain a trough value and cervicovaginal samples will be collected four hours after the morning dose. At the two week follow-up visit blood samples will be drawn prior to the morning dose to obtain a trough value and then at 0.5, 1, 2, 4, 6, 8, and 24 hour(s) from the final morning dose. Participants will be randomized to have cervicovaginal samples and biopsies collected at either 4, 8 or 24 hours after the final morning dose.

Up to 10 participants at the University of Pittsburgh site, who have completed the first two phases of the study, will be asked to participate in a third phase to have cervicovaginal samples, biopsies and blood samples collected 12 hours after a single-dose.

Study Duration:
The clinical portion of the study will last about 12 months. Data analysis will take 3 months and the Final Report an additional month.

Study Team

1. **Sponsor: CONRAD**
   Medical Officer: Jill Schwartz, MD
   CONRAD Signatory/Clinical Director: Marianne Callahan

2. **Tenofovir Gel IND Holder: Family Health International (FHI)**
   IND # 73,382
   FHI Regulatory Affairs Director: Janet Robinson, MPH

3. **Data Management and Analysis: FHI**
   FHI Director of Data Management: Patrick Murphy
   FHI Director of Biostatistics: Doug Taylor, PhD

4. **Study Sites and Principal Investigators:**

   PROFAMILIA, Santo Domingo, Dominican Republic
   Principal Investigator: Vivian Brache, Lic.

   University of Pittsburgh/Magee-Womens Hospital, Pittsburgh, PA
   Principal Investigator: Mitch Creinin, MD

   Advances in Health, Inc., Houston, TX
   Principal Investigator: Alfred Poindexter III, MD

5. **Drug Manufacturer: Gilead, Inc.**

TABLE OF CONTENTS

I. INTRODUCTION 1

II. STUDY OBJECTIVES 4

III. ENDPOINTS 4

IV. TEST ARTICLES 4

V. STUDY DESIGN 4

VI. SELECTION OF PARTICIPANTS 7

- A. INCLUSION CRITERIA 7
- B. EXCLUSION CRITERIA 7
- C. POST-ADMISSION WITHDRAWAL CRITERIA 8

VII. STUDY PROCEDURES 9

- A. VISIT 1: SCREENING VISIT 9
- B. VISIT 2: ENROLLMENT VISIT 10
- C. VISIT 3: (TWO-WEEK PHASE OF ONCE OR TWICE-DAILY USE) 11
- D. VISIT 4: (ONE WEEK FOLLOW-UP) 12
- E. VISIT 5: (TWO WEEK FOLLOW-UP) 12
- F. VISIT 6: FINAL FOLLOW-UP 13
- G. SUBSTUDY: 12 HOUR TIME-POINT 14
- H. ADVERSE EVENT FOLLOW-UP/UNSCHEDULED VISIT 15
- I. EARLY DISCONTINUATION 16
- J. LABORATORY PROCEDURES 16

VIII. CRITERIA FOR DISCONTINUATION OF STUDY 16

IX. STUDY MATERIALS 17

X. STATISTICAL ANALYSIS AND SAMPLE SIZE CALCULATION 19

- A. SAMPLE SIZE JUSTIFICATION 19
- B. ANALYSIS PLAN 20

XI. MANAGEMENT OF INTERCURRENT EVENTS 22

- A. LOSS TO FOLLOW-UP 22
- B. PROTOCOL ADHERENCE 22
- C. ADVERSE EVENTS 23
- D. MANAGEMENT/REPLACEMENT OF PARTICIPANTS WHO DISCONTINUE 24
- E. PREGNANCY 25
- F. PROTOCOL VIOLATIONS 25
- G. MODIFICATION OF PROTOCOL 25

XII. REPORTING REQUIREMENTS 25

- A. STUDY INITIATION 25
- B. STUDY CONDUCT 26

XIII. MONITORING 26

XIV. USE OF INFORMATION AND PUBLISHING 26

XV. ANTICIPATED LENGTH OF STUDY **27**

XVI. CASE REPORT FORMS (CRFS) **27**

XVII. RECORDS RETENTION **27**

XVIII. PROCEDURE FOR OBTAINING INFORMED CONSENT **28**

A. INSTITUTIONAL REVIEW BOARD (IRB) REVIEW AND APPROVAL 28
B. INFORMED CONSENT 28
C. SUBJECT CONFIDENTIALITY 29

XIX. ADDITIONAL INVESTIGATOR RESPONSIBILITIES **29**

XX. REFERENCES **31**

APPENDIX I: STUDY FLOW CHART **32**

APPENDIX II: PARTICIPANT GUIDEBOOK: MAIN STUDY **34**

APPENDIX III: PARTICIPANT GUIDEBOOK: SUBSTUDY **47**

APPENDIX IV: BLOOD PARAMETERS* AND NORMAL VALUE RANGES **56**

APPENDIX V: LABORATORY PROCEDURES FOR PK ANALYSIS **58**

APPENDIX VI: DAIDS TABLE FOR GRADING THE SEVERITY OF ADVERSE EVENTS **62**

APPENDIX VII: MODEL CONSENT FORM **83**

APPENDIX VIII: DIARY CARD **99**

APPENDIX IX: DIARY CARD (FOR SUBSTUDY) **101**

I. INTRODUCTION

Tenofovir (the generic name for the active compound, interchangeable with its chemical name 9-[(R)-2-(phosphonomethoxy)propyl] adenine or PMPA) is currently being evaluated as a potential vaginal microbicide. A prodrug of tenofovir is currently approved for antiretroviral therapy (Viread® or tenofovir disoproxil fumarate [DF]). The prodrug, tenofovir DF, has substantially greater oral bioavailability than tenofovir. Tenofovir is a nucleotide analog that blocks viral replication by inhibiting reverse transcriptase; therefore, it can block local viral replication once infection has occurred. The nucleotide class of drugs bypasses the initial phosphorylation step necessary for activation of the nucleoside class of drugs (such as Retrovir®). Therefore, nucleotide analogs may possess greater activity in a broader range of activated or resting cell types and their lack of requirement for virus specific activation may decrease the propensity for the development of antiviral resistance. Tenofovir has shown activity in target immune cells: lymphocytes (T cells), monocyte/macrophages and dendritic (Langerhans) cells. Therefore, tenofovir gel is a vaginal microbicide candidate designed to prevent the sexual transmission of HIV.

Tenofovir 1% gel was well tolerated in rabbit vaginal toxicity tests. Six separate studies of vaginal transmission inhibition performed in non-human primates provided evidence for some degree of efficacy of tenofovir gel [1]. An intravaginal pharmacokinetic study using 1% tenofovir (14C labeled) gel in macaques showed penetration into the vaginal epithelium with peak tissue concentration at 8 hours (20.64 mg-eq/g, 6.6-fold greater than 15 minute tissue concentration) after exposure. No significant radioactivity was detected in whole blood or plasma. Tenofovir has been shown to block HIV-1 transmission across the mucosa in a human cervical tissue-derived organ culture model [2].

Rabbit vaginal tissue biopsies were evaluated for total tenofovir content 8 hours following intravaginal gel and intravenous single dose and 4 hours following intravaginal dosing (single dose, twice daily for one week and twice daily for two weeks). Intraveneous and intravaginally administrated tenofovir was detected in vaginal rinse, tissue biopsies as well as blood samples. Surprisingly, mean vaginal tissue concentration of tenofovir collected 8 hours post dose were similar following single intravenous and intravaginal administration (950 ng/mL and 940 ng/mL, respectively). The mean vaginal tissue concentrations of tenofovir collected 4 hours post intravaginal dose (single dose, twice daily for one week and twice daily for two weeks) were higher than those at 8 hours (2,817 ng/mL, 3,146 ng/mL and 11,409 ng/mL, respectively). Systemic absorption following single intravaginal administration of 1% tenofovir gel was barely detectable and only so within the first 30 minutes. Multiple intravaginal dosing (twice daily for 1 week and twice daily for 2 weeks) resulted in low systemic levels of tenofovir (Cmax =239 ng/mL and 71 ng/mL, respectively, vs. 10,211 ng/mL following single intravenous dose). Tenofovir $AUC_{0\text{-}4hr}$ following a single IV dose was ten times greater than following the last daily dose for either intravaginal multiple dosing regimen. No vaginal irritation was observed following intravenous tenofovir. Minimal irritation was reported following a single intravaginal dosing or twice daily for a week. Mild vaginal irritation was reported following twice daily intravaginal dosing for two weeks.

Tenofovir (0.3% and 1%) gel has recently been tested in a Phase I study conducted by the HIV Prevention Trials Network (HPTN) assessing the safety and acceptability of tenofovir gel used vaginally either once or twice daily for 14 days among sexually abstinent and sexually active HIV-uninfected, and HIV-infected women. Twenty-five of the women in the study had PK testing with the limit of quantitation: 3.0 ng/mL.

Fourteen of 25 women (56%) had low, but detectable, serum tenofovir levels at some point in the 12 hours after dosing on either Day 0 (following the first dose) or on Day 13 (after daily dosing); three of the 14 had detectable levels on both days (Figure 1) [3]. The maximum tenofovir concentrations (Cmax) ranged from 3.1 to 25.8 ng/mL, with no clear dose-concentration relationship identified. For the participant with the 25.8 ng/mL level, this peak level occurred 2 hours following the dose; the level rapidly declined to 10.8 ng/mL at 4 hours and was undetectable at 12 hours following the dose. Besides this outlier with the highest tenofovir level, the next highest Cmax was 7.1 ng/mL. Considering all women in the PK cohort, the median tenofovir Cmax was 3.4 ng/mL (interquartile range: below limit of quantitation [3.0 ng/mL] to 4.7 ng/mL) which corresponds to approximately 1% of the maximum and 7% of the minimum blood concentrations following multiple oral doses of tenofovir DF 300 mg [3]. The serum levels fell below the detection limit in approximately 87% of all samples taken between 0.5 and 24 hours.

Figure 1 demonstrates tenofovir blood concentration following vaginal administration of 1% tenofovir gel. All levels for all women with measurable tenofovir levels in the blood are shown. (14 of 25; lower limit of quantitation (LLOQ) approximately 3.0 ng/mL [dotted line]). The legend indicates "cohort" – "ID" – "study day". For reference the tenofovir level associated with the median 24 hour post-dose blood concentration following an oral 300 mg tenofovir dose is indicated with dashed line [4].

FIGURE 1 - TENOFOVIR BLOOD CONCENTRATIONS VS. TIME AFTER VAGINAL ADMINISTRATION

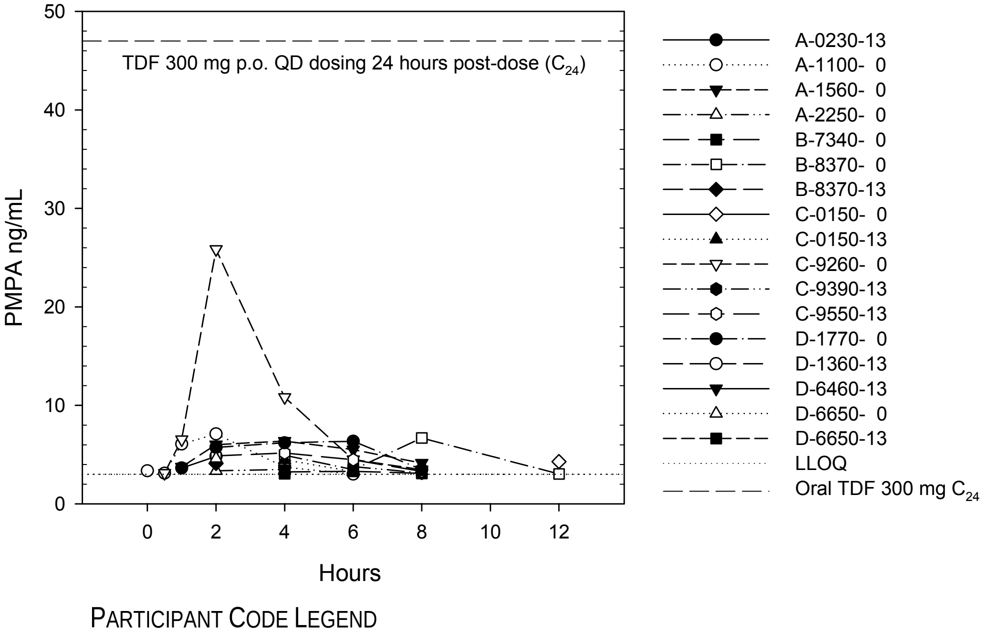

PARTICIPANT CODE LEGEND
First letter = Cohort
Cohort A - HIV -uninfected/sexually abstinent
Cohort B - HIV -uninfected/sexually active
Cohort C - HIV-infected/sexually abstinent
Cohort D - HIV-infected/sexually active
4 digits = last 4 digits of participant identifier
Last digit = day of study

No clinically significant systemic toxicity was detected in this study. Therefore, it can be concluded that tenofovir 1% vaginal gel used twice daily was well tolerated in abstinent and sexually active HIV uninfected and HIV infected women, with limited systemic absorption. Extended safety and effectiveness studies are warranted based on these initial data.

A phase II study to determine the safety of tenofovir 1% intravaginal gel used for 24 weeks, and to gain additional information regarding the product's acceptability is ongoing: it is a four arm, randomized controlled trial, comparing two frequencies of use (daily and coitally dependent), and corresponding placebo gel arms.

One of the concerns related to the use of tenofovir as a microbicide in the gel form is the uncertainty regarding the timeframe during which it will be converted to the active antiviral metabolite and be expected to potentially provide protection during coitus. Currently we do not know how long it will take the immune cells in the vaginal mucosa to convert tenofovir to its di-phosphorylated anti-retroviral metabolite. What we do know is that the activation of tenofovir is dependent upon anabolic phosphorylation by intracellular nucleoside kinases, whose activity and availability are dependent upon the activation state of the cell. Thus it is possible that the absolute rate of tenofovir activation will be dependent upon a wide range of cervico-vaginal factors, including inflammatory state and hormonal influences, as well as the physiochemical properties of tenofovir gel in the vagina.

A number of *in vitro* studies have indicated that the uptake and metabolism of tenofovir to its active metabolite by immune cells is a rapid process, giving rise to potentially antiretroviral levels of tenofovir diphosphate with intracellular half-lives in the range of 10 to 50 hours [5]. Radiolabeled (3H) tenofovir is rapidly taken up by resting and activated lymphocytes, suggesting that cellular uptake is via endocytosis. Subsequent metabolism of tenofovir appears to proceed quickly. The mono- and di-phosphate metabolites accumulate rapidly, reaching approximately 0.1 micromolar and approximately 0.3 micromolar, respectively, at 6 hours after exposure, whereas the original drug is not detectable (within 0.5 to 24 hours). This suggests that tenofovir is rapidly processed upon entry to the cell to the mono- and then to the di-phosphate metabolite. The metabolic properties of tenofovir may facilitate the formation and maintenance of a barrier to virus replication that could be effective for coital-dissociated microbicide applications.

Although information about the uptake and metabolism of tenofovir in the local cervicovaginal tissues of humans after vaginal application is needed, the long intracellular half-life of tenofovir supports a daily or twice-daily clinical dosing regimen. If uptake and metabolism to its active form in human cervicovaginal tissue is rapid, then coitally-associated application may also be feasible. The optimal anti-retroviral vaginal microbicide would work locally at the cellular level to prevent HIV transmission, would not accumulate in the cervicovaginal tissues, and would not be absorbed systemically through the blood or lymphatic system.

Since the hypothesized key mechanism of action of intravaginal tenofovir is inhibition of viral replication locally, adequate tissue concentration in the genital tract is crucial. The current study will assess the local and systemic absorption and concentration of tenofovir in 49 women in a single-dose phase followed by a once or twice-daily two week phase to determine genital tract concentration and to develop an evidence based strategy to select a gel dosing strategy.

II. STUDY OBJECTIVES

- To assess the local absorption and concentration of tenofovir after a single dose and after 14 days of once or twice-daily dosing.

- To assess the systemic absorption of tenofovir after a single dose and after 14 days of once or twice-daily dosing.

III. ENDPOINTS

- Concentration of tenofovir after single tenofovir gel vaginal dose in local genital tract compartments (intraluminal, mononuclear cells and vaginal tissue levels).

- Concentration (Cmax) and time of maximum concentration (Tmax) of tenofovir after single tenofovir gel vaginal dose in the systemic compartment.

- Concentration of tenofovir after two weeks of once and twice-daily vaginal administration of tenofovir gel in local genital tract compartments (intraluminal, mononuclear cells and vaginal tissue levels).

- Concentration (Cmax), time of maximum concentration (Tmax), and time to one half concentration (t½) of tenofovir after two weeks of once and twice-daily vaginal administration of tenofovir gel in the systemic compartment.

IV. TEST ARTICLES

Tenofovir gel is a clear, transparent, viscous gel packaged in epoxy inner-lined aluminum tubes with a white polyethylene screw cap equipped with a puncture tip. Each tube contains 7 grams of 1% tenofovir gel (weight/weight) formulated in purified water with edetate disodium, citric acid, glycerin, methylparaben, propylparaben, hydroxyethylcellulose, and pH adjusted to four to five. The gel is applied with a polyethylene applicator capable of administering a 4mL (equal to four gram) dose of gel. All study products will be stored at room temperature (15º-30º C).

V. STUDY DESIGN

This is a multi-center, pharmacokinetic study involving a single-dose phase, a wash out phase and a two week once or twice-daily dosing phase for each of 49 participants. University of Pittsburgh and Advances in Health will each enroll 14 participants and PROFAMILIA will enroll 21 participants. To construct a PK curve after a single vaginal administration of tenofovir gel, repeated biopsies over time are necessary; however, since repeat biopsies over time in the same individual is not feasible, participants will be randomly assigned to one of the seven time-points and there will be seven volunteers for each time-point.

In the single-dose phase, each volunteer will undergo a screening exam and if she is determined to be eligible and has given informed consent, she will apply the single dose in the clinic and will ambulate for approximately 15 minutes to distribute the gel after which she may ambulate as desired. Cervicovaginal samples and biopsies will be collected at one of seven time-points [0.5, 1, 2, 4, 6, 8, and 24 hour(s)] after the single dose. Blood samples will be drawn at 0.5, 1, 2, 4, 6, 8, and 24 hour(s)

after the single dose as clinically feasible. An interim analysis of approximately 21 participants (ideally 7 at each site) measuring tenofovir in plasma, vaginal biopsies, and cervicovaginal fluid following the initial dose will be performed.

There will be at least a one month wash out period between the one week phase and the two week phase.

In the two week phase, the study supplies will be distributed and the participants will be randomized to vaginally administer each dose either once or twice-daily for two weeks. At the one week follow–up visit, a blood sample will be drawn prior to the morning dose to obtain a trough value and cervicovaginal samples will be collected four hours after the morning dose. At the two week follow-up visit, blood samples will be drawn prior to the morning dose to obtain a trough value and at 0.5, 1, 2, 4, 6, 8, and 24 hour(s) after the final morning dose. Cervicovaginal samples and biopsies will be collected at 4, 8 or 24 hours after the final morning dose. It is expected that after two weeks of dosing, a steady state should be reached.

Up to 10 participants at the University of Pittsburgh site, who have completed the first two phases of the study, will be asked to participate in a third phase to have cervicovaginal samples, biopsies and blood samples collected 12 hours after a single-dose. This substudy is necessary because it would be logistically difficult to collect samples at 12 hour time-points in the main study (they would have to be collected during the night).

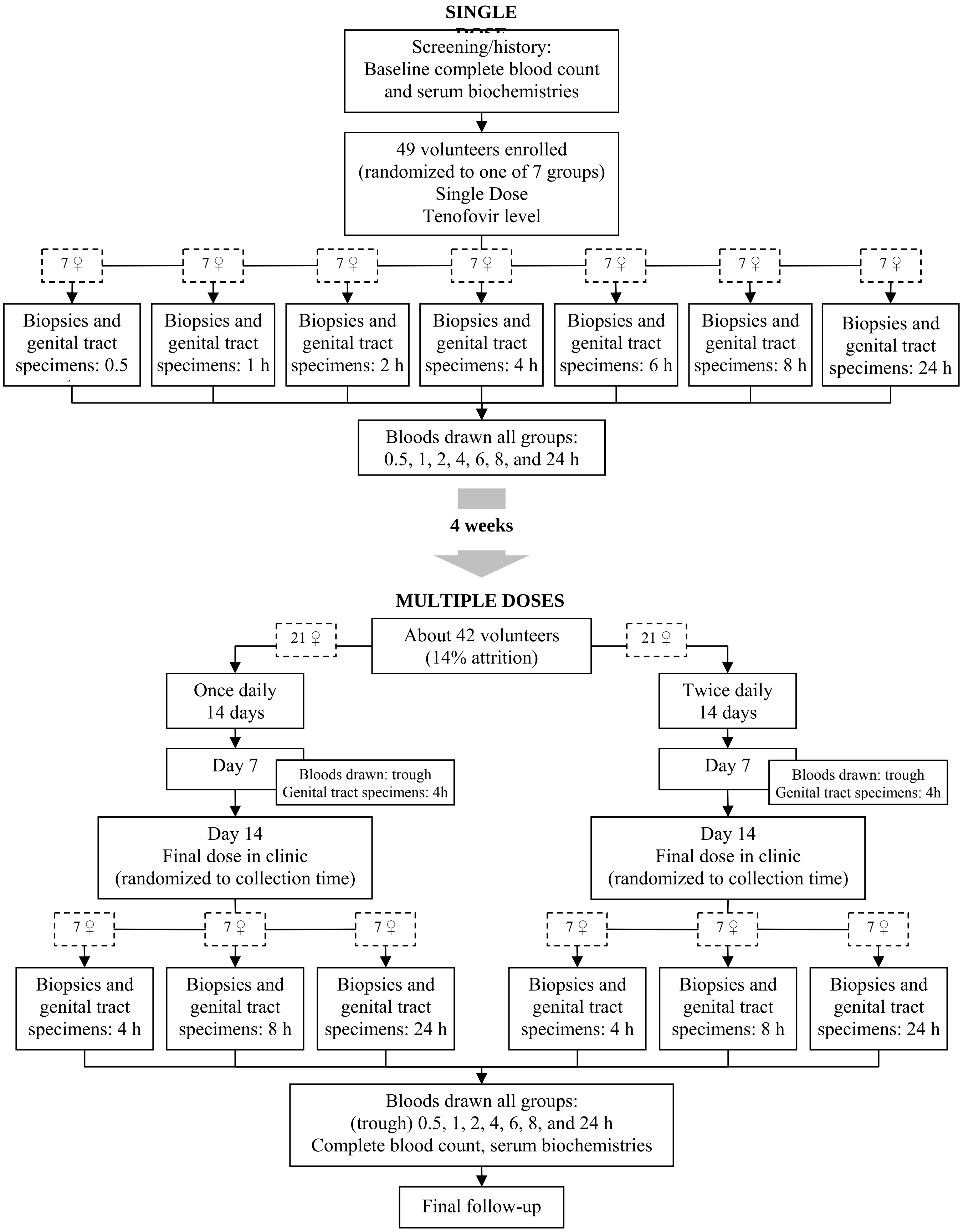
SINGLE
Screening/history: Baseline complete blood count and serum biochemistries
49 volunteers enrolled (randomized to one of 7 groups) Single Dose Tenofovir level
7 ♀
Biopsies and genital tract specimens: 0.5
Biopsies and genital tract specimens: 1 h
Biopsies and genital tract specimens: 2 h
Biopsies and genital tract specimens: 4 h
Biopsies and genital tract specimens: 6 h
Biopsies and genital tract specimens: 8 h
Biopsies and genital tract specimens: 24 h
Bloods drawn all groups: 0.5, 1, 2, 4, 6, 8, and 24 h
4 weeks
MULTIPLE DOSES
21 ♀
About 42 volunteers (14% attrition)
21 ♀
Once daily 14 days
Twice daily 14 days
Day 7
Bloods drawn: trough Genital tract specimens: 4h
Day 7
Bloods drawn: trough Genital tract specimens: 4h
Day 14 Final dose in clinic (randomized to collection time)
Day 14 Final dose in clinic (randomized to collection time)
7 ♀
Biopsies and genital tract specimens: 4 h
Biopsies and genital tract specimens: 8 h
Biopsies and genital tract specimens: 24 h
Biopsies and genital tract specimens: 4 h
Biopsies and genital tract specimens: 8 h
Biopsies and genital tract specimens: 24 h
Bloods drawn all groups: (trough) 0.5, 1, 2, 4, 6, 8, and 24 h Complete blood count, serum biochemistries
Final follow-up

VI. SELECTION OF PARTICIPANTS

The volunteers for this study will be recruited through existing databases, as protected health information permits, and, if necessary, through advertisements in local newspapers and radio which have been approved by the site's IRB.

Only women will be recruited since intravaginal gel can only be used by women. Although the NIH has mandated that children (defined as anyone under the age of 21) be included in research trials when appropriate, this study will enroll "children," aged 18 and older who are able to give informed consent. This study fits one of the "Justifications for Exclusion" as set forth by the NIH for excluding younger children. Specifically, "insufficient data are available in adults to judge potential risk in children" and "children should not be the initial group to be involved in research studies." Efforts will be made by the sites to recruit subjects so that the racial and ethnic characteristics of the subject population will reflect the demographics of the study sites. No exclusion criteria shall be based on race or ethnicity.

A. Inclusion Criteria

- Must be between 18-45 years old, inclusive;
- Must be in general good health by volunteer history without any clinically significant systemic disease (including liver disease/hepatitis, gastrointestinal disease, kidney disease, thyroid disease, osteoporosis or bone disease);
- Must have regular menstrual cycles (minimum of 26 and maximum of 38 days);
- Must be at least 2 months since last pregnancy outcome and have had at least two spontaneous menses;
- Must abstain from sexual activity and use of intravaginal products for 72 hours prior to the start of the single-dose phase and multi-dose phase, for at least one week following vaginal biopsies and for the duration of the study phases;
- Willingness to give voluntary consent, sign an informed consent form and comply with study procedures as required by the protocol;

B. Exclusion Criteria

- Currently pregnant or at risk for pregnancy (may be using Paragard® IUD, effective barrier method, female sterilization or abstinence, or be sexually active with a vasectomized partner);
- Currently breastfeeding or planning to breastfeed during the course of the study;
- Use of any hormonal contraceptives within 30 days of enrollment;
- Use of Depo-Provera within 120 days of enrollment;

- History of abnormal Pap smear (by volunteer history) that has not been evaluated and treated, if indicated, according to standard guidelines;

- Current vaginal or urinary tract infection;

- Positive test for *Neisseria gonorrhea* or *Chlamydia trachomatis* at the time of screening;

- Positive wet mount for *Trichomonas vaginalis* at the time of screening or enrollment;

- Deep epithelial genital findings such as abrasions, ulcerations, and lacerations, or vesicles suspicious for a sexually transmitted infection;

- Current or past use of any anti-retroviral therapies including but not limited to systemic tenofovir (Viread®);

- Current or recent drug or alcohol abuse;

- Current or anticipated use of drugs on a daily basis that may reduce renal function (e.g. acyclovir or ibuprofen) or liver function (e.g. tylenol);

- HIV positive at the time of screening;

- Hepatitis B surface antigen (HBsAg) positive at the time of screening;

- Grade 1 or higher serum chemistry or complete blood count abnormality in accordance with DAIDS toxicity table values (See Appendix VI). **Note:** In Appendix IV, the table of Lab Normal Values indicates any modifications by CONRAD to DAIDS' accepted normal values;

- Abnormal finding on laboratory or physical examination or a medical condition which, in the opinion of the investigator, would make participation in the study unsafe or would complicate interpretation of data;

- Participation in any other research study in the 30 days prior to screening and/or plans to participate in any other research study during entire study duration.

C. Post-Admission Withdrawal Criteria

- Failure to follow protocol requirements that are judged severe enough by the investigator to significantly affect study outcomes;
- Pregnancy;
- Medical reasons; or
- Personal reasons (volunteer request).

VII. STUDY PROCEDURES

A. Visit 1: Screening Visit

Volunteers will be given information about and requirements of the study. Volunteers will be asked if they are willing to participate in both phases of the study. All volunteer questions regarding the study will be answered. If the volunteer is eligible and wishes to participate in the study, she will be asked to sign an informed consent and the following will be performed:

- an interview to obtain medical history and demographic information;
- pre-test HIV counseling;
- a urine sample will be obtained for a urine pregnancy test to confirm that the volunteer is not pregnant and to perform a dipstick urinalysis (urine microscopy and culture will be performed as clinically indicated);
- baseline blood sample will be collected for complete blood count (CBC), serum chemistries, Hepatitis B surface antigen (HBsAg) test and HIV testing; and
- height and weight will be measured and recorded;
- screening gynecological examination:
    - a vaginal sample will be collected for wet mount/pH,
    - cervical swabs will be taken to test for *Neisseria gonorrhea* (NG) and *Chlamydia trachomatis* (CT),
    - a bimanual pelvic examination will be performed.

Women who test HIV positive at screening are not eligible for enrollment. They will be counseled and given referrals for medical and social services. If symptomatic yeast, BV, or a UTI is clinically diagnosed at this visit, the volunteer will be treated, preferably with oral medication and, if she is otherwise eligible, the enrollment visit will be scheduled after completion of treatment and resolution of symptoms. If *Neisseria gonorrhoea, Chlamydia trachomatis, or Trichomonas vaginalis* is diagnosed the volunteer will be treated or referred for treatment and will not be eligible for enrollment. If deep epithelial disruptions (such as abrasions, ulcerations, or lacerations) or vesicles suspicious for a sexually transmitted infection are observed on the female genitalia the participant will not be eligible for enrollment and testing should be performed as clinically indicated or she should be referred for treatment, as appropriate. If deep epithelial disruption is identified that is either iatrogenic or unlikely to have been caused by infection and likely to resolve quickly, the appointment can be rescheduled, and the participant may be enrolled in the study once the finding has resolved. Eligible volunteers will be scheduled for an enrollment visit when they are not menstruating and an attempt will be made to schedule the visit in the second half of the menstrual cycle, ideally menstrual cycle day 19-24. The volunteer will abstain from sexual activity and use of intravaginal products for 72 hours prior to the enrollment visit (start of the single-dose phase) and for the duration of the study phase and will be instructed to abstain from the following activities during this time:

- Masturbation;
- Oral contact with the female genitalia;
- Vaginal and anal intercourse;
- Douching;
- Penetration of the vagina by fingers, tampons, sex toys, or any other objects; and

- Use of any vaginal product other than the study gel including spermicides, lubricants, vaginal hygiene products, diaphragms, and cervical caps.

B. Visit 2: Enrollment Visit

The enrollment visit should take place within 8 weeks of the screening visit. The results from the screening visit will be reviewed. Volunteers with abnormal blood screening test results will be advised to seek advice and/or care from their primary care provider or referrals to appropriate resources will be given. A urine sample will be obtained for a urine pregnancy test to confirm that the volunteer is not pregnant. A pelvic examination will be performed in which the vulva, vagina and cervix will be visually inspected for non-intact epithelium or other findings. If deep epithelial disruption is identified, the participant will not be enrolled until the finding has resolved.

If eligible, the volunteer will be enrolled and randomized to one of seven time-points [0.5, 1, 2, 4, 6, 8, and 24 hour(s)] after the single dose for collection of cervicovaginal samples and biopsies. A baseline blood sample for systemic tenofovir level will be drawn. The participant will be instructed to void prior to the first dose. The participant will apply the single-dose in the research unit and will ambulate for approximately 15 minutes to distribute the gel, after which she may ambulate as desired. The participant will be instructed to avoid emptying her bladder or bowel within the first hour after application, if possible.

Blood samples will be collected at 0.5, 1, 2, 4, 6, 8, and 24 hour (s) after the single-dose for plasma measurement of tenofovir. See protocol section XI.B for protocol adherence definitions. The participant will leave the research unit after the 8 hour blood draw and will return the following day for the 24 hour blood draw (as well as cervicovaginal samples and biopsies for those assigned to the 24 hour time-point); therefore, this visit will occur over a 24 hour time period. The participant will be asked about vaginal activity and adverse events that occur between the 8 hour and the 24 hour visit. Each participant will undergo the following pelvic examination procedures at her randomly assigned time-point:

- The volunteer should be supine for 15 minutes before the pelvic examination to allow for pooling to occur.
- Direct aspiration of the cervicovaginal fluid pooling (avoiding tenofovir gel when possible) in the posterior fornix with a vaginal aspirator syringe will be performed to measure intraluminal concentration of tenofovir.
- Endocervical specimen will be collected with two 360º turns of a cytobrush to measure local intracellular tenofovir in mononuclear cells.
- The areas of the planned biopsies will be gently wiped with a small cotton swab moistened with warm saline followed by a small cotton swab with betadine.
- Topical 20% benzocaine gel will be applied to the areas of the planned biopsies for pain control. Women can opt to have the biopsy without anesthesia by preference or if they are allergic to benzocaine or can be given non-steroidal anti-inflammatory drugs prior to the procedure for pain relief as per local site standards.
- Full-thickness biopsies measuring approximately 5mm by 3mm will be obtained from an area located on the upper one-third of the vagina (at around 7-8 o'clock), and from the lower

one-third of the vagina (at around 7-8 o'clock) with cervical biopsy forceps to measure tissue concentration of tenofovir.
- If adequate hemostasis is not obtained with gentle pressure, Monsel's solution or silver nitrate can be applied, as needed, to the biopsy site.

The volunteer will be given a Participant Guidebook (Appendix II) that will give detailed instructions on how to insert the tenofovir, as well as what she can expect during each visit. The guidebook will include post-biopsy instructions that include avoiding sexual activity, douching or tampon use for at least a week following the biopsy to allow time for the biopsy site to heal.

The volunteer will also abstain from sexual activity and use of intravaginal products for 72 hours prior to Visit 3 (start of the two-week phase) and for the duration of the study phase and should be instructed to abstain from the following activities during this time:

- Masturbation;
- Oral contact with the female genitalia;
- Vaginal and anal intercourse;
- Douching;
- Penetration of the vagina by fingers, tampons, sex toys, or any other objects
- And use of any vaginal product other than the study gel including spermicides, lubricants, vaginal feminine hygiene products, diaphragms, and cervical caps.

There will be at least 30 days between this visit and the beginning of the two week phase to ensure total elimination of the drug from the participant and healing of the biopsy sites.

C. Visit 3: (Two-week Phase of once or twice-daily use)

This visit will be scheduled for when the volunteer is not menstruating and should be scheduled early in the menstrual cycle so that the last dose at the time of the two week follow-up will occur in the second half of the menstrual cycle, ideally menstrual cycle day 19-24.

A urine pregnancy test will be performed on each volunteer to confirm that she is not pregnant. Blood will be drawn to obtain systemic levels of tenofovir. A pelvic examination will be performed in which the vulva, vagina and cervix will be visually inspected for non-intact epithelium or other findings, and to verify healing from the single-dose phase. If deep epithelial disruption is identified, the participant will not continue in the study until the finding has resolved.

Participants will be randomly assigned to either once-daily or twice-daily application and will receive study supplies. Participants assigned to once-daily use will be instructed to insert the gel in the mornings at approximately 24-hour intervals. Participants assigned to twice-daily use will be instructed to insert the gel in the mornings and evenings at approximately 12-hour intervals. All participants will be instructed to begin dosing on the day after Visit 3.

Participants will be instructed to void prior to applying each dose and to ambulate for approximately 15 minutes after each application, after which they may ambulate as desired, and, to avoid emptying their bladders or bowels within the first hour after application, if possible.

If a participant misses a dose, she should insert it when she remembers, if possible, and then resume her regular schedule. If, at the time that she becomes aware that she missed a dose, it is within 6 hours of the next scheduled dose she should skip the missed dose and resume her regular schedule. If the participant misses two or more doses she should contact the study site for further instructions. The participant will be given instructions for gel use and a diary card to complete the date and time of all product applications and symptoms that occur during the study.

The participant will be instructed to return for a one week follow-up and will be asked to wait to insert her morning dose on the day of the scheduled visit as it will be applied at the clinic.

D. Visit 4: (One week follow-up)

A urine pregnancy test will be performed to confirm that the participant is not pregnant. The diary card will be reviewed and participants will be asked about any adverse events and will be evaluated, if necessary, as described in Section VII. H. A blood sample will be drawn prior to the morning dose to obtain a trough plasma measurement of tenofovir. The morning dose will be applied in the research unit.

Each participant will undergo the following pelvic examination procedures at 4 hours after the morning dose:

- Participants should be supine for 15 minutes before the pelvic examination to allow for pooling to occur.
- Direct aspiration of the cervicovaginal fluid pooling in the posterior fornix with a vaginal aspirator syringe will be performed to measure intraluminal concentration of tenofovir.
- Endocervical cytobrush specimen will be collected with two 360° turns of a cytobrush to measure local intracellular tenofovir in mononuclear cells.

The participant will be instructed to resume her assigned dosing schedule and to return for a two week follow-up and will be asked to wait to insert her final morning dose as it will be applied at the clinic.

E. Visit 5: (Two week follow-up)

A urine pregnancy test will be performed to confirm that the participant is not pregnant. The diary card will be reviewed and collected and participants will be asked about any adverse events and will be evaluated, if necessary, as described in Section VII. H. A copy of the back of their diary card will be given to the participant to record any symptoms and medications between Visit 5 and Visit 6. Each participant will be randomized to one of three time-points (4, 8 or 24 hours after the final dose) to provide cervicovaginal samples and biopsies. A blood sample will be drawn prior to the morning dose to obtain a trough plasma measurement of tenofovir. The final morning dose (14th or 27th) will be applied in the research unit. Blood samples will be collected at 0.5, 1, 2, 4, 6, 8, and 24 hour (s) after the dose for plasma measurement of tenofovir. Complete blood count and serum chemistries will also be drawn with the final collection. Each participant will undergo the following pelvic examination procedures at her randomly assigned time-point:

- The volunteer should be supine for 15 minutes before the pelvic examination to allow for pooling to occur.

- Direct aspiration of the cervicovaginal fluid pooling (avoiding tenofovir gel when possible) in the posterior fornix with a vaginal aspirator syringe will be performed to measure intraluminal concentration of tenofovir.
- Endocervical specimen will be collected with two 360º turns of a cytobrush to measure local intracellular tenofovir in mononuclear cells.
- The areas of the planned biopsies will be gently wiped with a small cotton swab moistened with warm saline followed by a small cotton swab with betadine.
- Topical 20% benzocaine gel will be applied to the areas of the planned biopsies for pain control. Women can opt to have the biopsy without anesthesia by preference or if they are allergic to benzocaine or can be given non-steroidal anti-inflammatory drugs prior to the procedure for pain relief as per local site standards.
- Full-thickness biopsies measuring approximately 5mm by 3mm will be obtained from an area located on the upper one-third of the vagina (at around 4-5 o'clock) and from the lower one-third of the vagina (at around 4-5 o'clock) with cervical biopsy forceps to measure tissue concentration of tenofovir.
- If adequate hemostasis is not obtained with gentle pressure, Monsel's solution or silver nitrate can be applied, as needed, to the biopsy site.

If the participant will not be taking part in the substudy, Visit 6 will be scheduled for 1-2 weeks from this visit to allow time for the biopsy sites to heal.

<u>Substudy Participants only:</u> If the participant will be taking part in the substudy, she will not have a Visit 6 in the main study, but instead will be scheduled for Substudy Visit 1. This visit will be scheduled for when the participant will not be menstruating and an attempt will be made to schedule the visit in the second half of the menstrual cycle, ideally menstrual cycle day 19-24. There will be at least 30 days between this visit and the beginning of the substudy to ensure total elimination of the drug from the participant and healing of the biopsy sites.

The volunteer will also abstain from sexual activity and use of intravaginal products for 72 hours prior to Substudy Visit 1 (start of the substudy) and for the duration of the substudy. These activities include:

- Masturbation;
- Oral contact with the female genitalia;
- Vaginal and anal intercourse;
- Douching;
- Penetration of the vagina by fingers, tampons, sex toys, or any other objects
- And use of any vaginal product including spermicides, lubricants, vaginal feminine hygiene products, diaphragms, and cervical caps.

F. Visit 6: Final follow-up

The participant will return 1-2 weeks after Visit 5. A urine pregnancy test will be performed. The participant will be asked about any adverse events since her last visit and the copy of the symptoms and medications portion of her Diary Card will be collected. A gynecological examination will be

performed in which the vulva, vagina and cervix will be visually inspected for non-intact epithelium or other findings, and to verify healing from the biopsies. If the examination is normal the participant will be exited from the study. Otherwise the participant will be followed until the exam is normal or she is referred for follow-up care, as clinically indicated.

G. Substudy: 12 hour time-point

Substudy (Visit 1)

Up to 10 participants at the University of Pittsburgh site who have completed the first two phases of the study will be asked to participate in a third phase. This substudy is necessary because it would be logistically difficult to collect samples at 12 hour time-points in the main study (they would have to be collected during the night).

A baseline blood sample for systemic tenofovir level will be drawn. A urine sample will be obtained for a urine pregnancy test to confirm that the volunteer is not pregnant. A pelvic examination will be performed in which the vulva, vagina and cervix will be visually inspected for non-intact epithelium or other findings. If deep epithelial disruption is identified, the participant may continue in the study when the finding has resolved.

The participant will be given a single-dose to use at home in the evening at a specified time, along with a Substudy Diary Card and instructions. The participant will be instructed to void prior to inserting the dose. The participant will apply the single-dose at home (at a time that will fall approximately 12 hours before her next visit) and will ambulate for approximately 15 minutes to distribute the gel, after which she may ambulate as desired. She will be scheduled to return to the research clinic the following morning.

Substudy (Visit 2)

Blood samples will be collected 12 hours after the single dose for plasma measurement of tenofovir. The diary card will be reviewed and collected and participants will be asked about any adverse events and will be evaluated, if necessary, as described in Section VII. H. A copy of the Diary Card will be given to the participant to record any symptoms and medications between Substudy Visit 2 and Substudy Visit 3. Each participant will also undergo the following pelvic examination procedures at the 12 hour time-point:

- The volunteer should be supine for 15 minutes before the pelvic examination to allow for pooling to occur.
- Direct aspiration of the cervicovaginal fluid pooling (avoiding tenofovir gel when possible) in the posterior fornix with a vaginal aspirator syringe will be performed to measure intraluminal concentration of tenofovir.
- Endocervical specimen will be collected with two 360º turns of a cytobrush to measure local intracellular tenofovir in mononuclear cells.
- The areas of the planned biopsies will be gently wiped with a small cotton swab moistened with warm saline followed by a small cotton swab with betadine.
- Topical 20% benzocaine will be applied to the areas of the planned biopsies for pain control. Women can opt to have the biopsy without anesthesia by preference or if they are allergic to

benzocaine or can be given non-steroidal anti-inflammatory drugs prior to the procedure for pain relief as per local site standards.

- Full-thickness biopsies measuring approximately 5mm by 3mm will be obtained from an area located on the upper one-third of the vagina (at around 7-8 o'clock), and on the lower one-third of the vagina (at around 7-8 o'clock) with cervical biopsy forceps to measure tissue concentration of tenofovir.
- If adequate hemostasis is not obtained with gentle pressure, Monsel's solution or silver nitrate can be applied, as needed, to the biopsy site.

Post-biopsy instructions will be given to the participant that include avoiding sexual activity, douching or tampon use for at least a week following the biopsy to allow time for the biopsy site to heal.

Substudy (Visit 3)

The participant will return 1-2 weeks after substudy Visit 3. A urine pregnancy test will be performed on all participants. The participant will be asked about any adverse events since her last visit and the copy of the Substudy Diary Card will be collected. A gynecological examination will be performed in which the vulva, vagina and cervix will be visually inspected for non-intact epithelium or other findings, and to verify healing from the biopsies. If the examination is normal the participant will be exited from the study. Otherwise the participant will be followed until the exam is normal or she is referred for follow-up care, as clinically indicated.

H. Adverse Event Follow-up/Unscheduled Visit

If the participant experiences moderate to severe symptoms (Appendix VI) including vaginal burning, itching, pain, spotting or bleeding at any time during product usage, she will be instructed to contact the clinic and may be evaluated with laboratory testing, physical examination and visual and colposcopic inspection as clinically indicated. If product is visible, an attempt should be made to rinse it off using normal saline. If a wet mount and/or STI testing are performed and positive for infection, the subject will be treated and discontinued from the study. The subject may continue in the protocol if the wet prep is negative, there is no STI diagnosed, there are no findings of deep epithelial disruption on the genitalia severe enough to make further application of the product inadvisable, the investigator feels that the participant's health is not significantly jeopardized, and the participant wishes to continue.

If a participant experiences severe irritation and cannot get to the clinic the same day, she will be instructed to stop product use, soak in a sitz bath, and douche with plain warm water. The volunteer will be encouraged to come to the clinic as soon as possible for evaluation.

In addition, she will be given post-biopsy instructions to contact the clinic if she experiences vaginal bleeding (if more than spotting), foul-smelling vaginal discharge, fever and/or chills, or severe lower abdominal pain and will be evaluated as clinically indicated.

If the participant reports symptoms of a urinary tract infection (UTI) at an unscheduled visit, a clean-catch urine dipstick and other urine testing as indicated will be performed. If a UTI is diagnosed the participant will be treated and discontinued from the study.

I. Early Discontinuation

If a participant is discontinued or withdrawn from the study early, final discontinuation procedures including urine pregnancy test, blood sample for systemic levels of tenofovir**,** complete blood count, and serum biochemistries should be performed**.** In addition, cervicovaginal specimens including vaginal biopsies should be obtained, if possible, and if relevant to study endpoints.

J. Laboratory Procedures

Vaginal wet mount/pH	At the screening visit, a sample of fluid taken with a cotton swab from the lateral fornix will be evaluated for signs of BV, trichomonas and/or candida. Vaginal pH will also be measured. If vaginal symptoms develop during the study, additional wet mounts will be done as indicated.
Neisseria gonorrhea (NG) and *Chlamydia trachomatis* (CT)	At the screening visit, an endocervical specimen will be testing for *N. gonorrhoeae* and *C. trachomatis* using Nucleic acid hybridization test or PCR
Urine pregnancy test	A urine specimen will be collected and tested according to the specifications of the test kit manufacturer at each scheduled visit and as indicated.
Dipstick urinalysis	At the screening visit, a clean catch urine sample will be tested via Multistix 10 SG Reagent Strips (Bayer) and as indicated at other visits.
CBC & Serum biochemistries, Hepatitis B, and HIV	Venous blood sample will be drawn and the serum will be tested for HBsAg and HIV antibodies at the screening visit. Samples will be processed according to the specifications of the laboratory (See Appendix IV). CBC and serum biochemistries will be measured at screening and at the 2-week follow-up visit.
RPR or VDRL and HSV	If a genital ulcer is present, screening for syphilis using the venereal disease research laboratory (VDRL) or rapid plasma regain (RPR) test may be performed and testing for *Herpes* simplex virus may be performed by obtaining a specimen for culture from the lesion. A positive screening test for syphilis will be confirmed using the FTA or TPPA test.

VIII. CRITERIA FOR DISCONTINUATION OF STUDY

The study may be stopped if, in the opinion of CONRAD:

1. Review of AEs shows an unexpected, significant or unacceptable risk to the participants enrolled in the study;

2. The site has failed to enroll subjects at an acceptable rate;

3. The protocol requirements have not been adhered to; and/or

4. Administrative reasons.

IX. STUDY MATERIALS

The sites will manage the distribution of the study product according to the protocol. Each site will be provided with a quantity of demonstration samples and study product kits equivalent to the number of patients they are to enroll. Supplies will be distributed by Bilcare Inc. (Phoenixville, PA) under CONRAD's supervision.

See section "IV. Test Articles" for a description of 1% tenofovir gel.

Each study tube will be labeled with the study number and lot number and the following information: "1% Tenofovir Gel, For Vaginal Use Only, Keep out of Reach of Children--Caution: New Drug: Limited by Federal Law to Investigational Use". See Appendix II for a copy of the participant instructions.

Single Use Kits: The single-dose kit will contain a labeled tube of 1% tenofovir gel (7 gram tube), an empty applicator and patient instruction sheet. See Appendix II for a copy of the participant instructions. The outside of the single-dose kit box will have a two-part, tear-off label, with the information listed below.

Single Dose and 14-Day Once or Twice-daily Pharmacokinetic Study of the Vaginal Microbicide Agent 1% Tenofovir Gel<br>Protocol # A04-095<br><br>**Single Use Kit**<br><br>Study sponsor: CONRAD, 1611 North Kent Street, Suite 806, Arlington, VA 22209<br><br>This product contains 1% tenofovir as active ingredient and methylparaben & propylparaben as gel preservatives.<br><br>Clinical supplies manufactured and packaged by Gilead Sciences, Inc.<br><br>Quantity: 1 tube of tenofovir gel; 1 disposable applicator; 1 participant instruction sheet<br><br>Participant Number: __________<br><br>Date 1 kit dispensed: _________________ (MM/DD/YY)<br><br>**CAUTION:** New Drug - Limited by Federal (U.S.) Law to Investigational Use.<br><br>For vaginal use only. Keep out of reach of children.<br>Store at room temperature (15°– 30°C) in a secure cabinet.

Multiple Dose Kits: There are two different multiple dose kits for once and twice-daily dosing for 14-days.

Once-a-day for 14-day kits will each contain 14 labeled tubes of 1% tenofovir gel (7 gram tube), 14 empty applicators and 1 patient instruction sheet. See Appendix II for a copy of the participant instructions. The outside of the multiple dose kit box will have a two-part, tear-off label, with the information listed below.

Single Dose and 14-Day Once or Twice-daily Pharmacokinetic Study of the Vaginal Microbicide Agent 1% Tenofovir Gel
Protocol # A04-095

Multiple Dose Kit: Once-a-day dosing

Study sponsor: CONRAD, 1611 North Kent Street, Suite 806, Arlington, VA 22209

This product contains 1% tenofovir as active ingredient and methylparaben & propylparaben as gel preservatives.

Clinical supplies manufactured and packaged by Gilead Sciences, Inc.

Quantity: 14 tubes of tenofovir gel; 14 disposable applicators; 1 participant instruction sheet

Participant Number: __________

Date kit dispensed: ________________ (MM/DD/YY)

Unused tubes returned: _______ Date returned: _____________________(MM/DD/YY)

CAUTION: New Drug - Limited by Federal (U.S.) Law to Investigational Use.

For vaginal use only. Keep out of reach of children.
Store at room temperature (15°– 30°C) in a secure cabinet.

Twice-a-day for 14-day kits will each contain 27 labeled tubes of 1% tenofovir gel (7 gram tube), 27 empty applicators and 1 patient instruction sheet. See Appendix II for a copy of the participant instructions. The outside of the multiple dose kit box will have a two-part, tear-off label, with the information listed below.

Single Dose and 14-Day Once or Twice-daily Pharmacokinetic Study of the Vaginal Microbicide Agent 1% Tenofovir Gel<br>Protocol # A04-095
**Multiple Dose Kit: Twice-a-day dosing**
Study sponsor: CONRAD, 1611 North Kent Street, Suite 806, Arlington, VA 22209
This product contains 1% tenofovir as active ingredient and methylparaben & propylparaben as gel preservatives.
Clinical supplies manufactured and packaged by Gilead Sciences, Inc.
Quantity: 27 tubes of tenofovir gel; 27 disposable applicators; 1 participant instruction sheet
Participant Number: __________
Date kit dispensed: ___________________(MM/DD/YY)
Unused tubes returned: _______ Date returned: _________________________(MM/DD/YY)
**CAUTION:** New Drug - Limited by Federal (U.S.) Law to Investigational Use.
For vaginal use only. Keep out of reach of children.<br>Store at room temperature (15°– 30°C) in a secure cabinet.

All used and unused study product at the end of the study will be recorded and disposed of by the clinical site.

Study product should be stored at room temperature (15°-30° C) in a locked cabinet or secure area in the clinic.

X. STATISTICAL ANALYSIS AND SAMPLE SIZE CALCULATION

A. Sample Size Justification

Statistical considerations were not the primary basis for choosing to enroll 49 women in this Phase I study; the study size was based primarily on large part on feasibility considerations (e.g., cost and time). Nonetheless, the chosen size should be sufficient to characterize relevant pharmacokinetic profiles. For example, in the previous HPTN vaginal dosing study that evaluated 25 women, maximum likelihood estimates of the standard deviation of logged plasma concentrations ranged from 0.41 to 0.82 at various time-points between hours 1 and 12 following initial dosing (these estimates account for censoring below the analytic limit of detection LOD through the use of log-normal likelihood methods). Based on these data, the expected half-width of 95% confidence intervals for mean (logged) plasma concentration in the proposed study, with forty nine women at each blood draw time-point, is 0.1 to 0.2. This compares favorably with expected mean plasma levels of about 0.5 logs. The use of nonlinear pharmacokinetic (PK) models to account for repeated observations on each participant should provide improved precision of predicted concentration curve time-points in the current study.

Although discussing the expected precision of vaginal tissue concentrations is rather speculative prior to performing this original study, we can conclude that by obtaining measurements from seven women at each time-point, and assuming similar standard deviations as observed in the plasma data, the half-

width of confidence intervals should be about 0.5 logs (compared to mean tissue levels that should be substantially larger than 0.5 for at least some of the sampled time-points).

B. Analysis Plan

Randomization, Allocation Concealment, and Blinding

A random permuted blocks method will be used to generate three random allocation sequences. The first sequence will be used to randomize women to one of seven biopsy time-points following initial dosing of the gel, stratified by site. The second sequence will be used to randomize women to either once- or twice-daily dosing in the 14 day follow-up period, stratified by site. The third sequence will be used to randomize women to one of the three concluding biopsy time-points (i.e., 4, 8 or 24 hours following the final, in-clinic, dosing), stratified by site and dosing arm within site. The random sequences will be created using a SAS (SAS Institute, Cary, NC) program validated by FHI.

It is not possible to blind clinic staff or study participants to the biopsy time-point assignments or dosing schedules once allocation has taken place. In order to conceal the allocation procedure, however, random assignments will be contained within sequentially numbered, sealed opaque envelopes. Participants will be randomized to biopsy time-points and a dose schedule by giving the next available randomization number to the next eligible participant. The randomization envelopes will be maintained in a secure location at the clinic. Each envelope will be used only once. For documentation purposes, a sealed copy of the allocation list will be sent to CONRAD and a second copy will be maintained at FHI. CONRAD and FHI staff working on the trial will not have access to the sealed copies nor any electronic data identifying assignments prior to the scheduled un-blinding of data analysts and other study staff not working at the clinic.

Statistical Considerations

A detailed analysis plan will be developed and approved prior to the end of data collection. The following is a summary of the planned analyses. Any changes made from this summary with respect to primary study outcomes will be documented in the detailed analysis plan.

Analysis Populations

A number of different analysis populations will be employed in this study. Although all study data will be summarized, the primary analysis set will exclude data collected from women who failed to comply with their assigned dosing schedule. Data points that fall outside of the allowed collection window (as specified in the protocol, Section XI.B) will also be excluded from analyses that depend on specific sampling times, unless the observed sampling time falls in an allowable (alternative) window. Further details regarding the analysis sets used for each primary and secondary analysis will be provided in the expanded analysis plan.

Censored Data

Based on the HPTN vaginal dosing study, it is anticipated that a large percentage of plasma tenovofir measurements will fall below the analytic limit of detection. Likewise, some censoring of data from the genital tract compartments is expected. Consequently, the plasma and genital tract concentration data will be described in two different ways: using medians, inter-quartile ranges, and ranges (in

which case censored data contribute as percentiles of the distributions); and by employing appropriate empirical PK models (e.g., one-compartmental models) that incorporate data censored below the analytic limit of detection (LOD) using appropriate likelihood techniques.

Analysis of Primary Endpoints

Two vaginal biopsy samples will be collected from each woman at each of their assigned time points (i.e. within 24 hours of their initial, single-dose and then, following a washout period, within 24 hours after their 14-day dosing regimen). The geometric mean concentration of tenofovir following the initial (day zero) dosing will be plotted for the maximum of 7 subjects per time period. Cmax and Tmax values will be estimated based on the median response values across time. The lack of repeat sampling times from each subject (i.e., biopsy samples are only taken at one time point for each woman in each phase of the study) invalidates the formal use of PK modeling for these data. However, nonlinear models (e.g., one- compartmental models) will be employed in an empirical exercise to obtain complimentary estimates of Cmax, Tmax, and their standard errors. The models will be validated to the extent possible given the available data.

Intraluminal tenofovir concentrations and cytobrush specimen results will be summarized in a similar fashion to biopsy samples. The concentration of tenofovir in blood following the initial (single) dosing will also be plotted over time for each subject. Cmax, Tmax, and T½ values will be obtained for each subject and summarized across subjects using medians (Cmax and T½ values will be imputed for those subjects whose measurements all fall below the LOD.). Cmax, Tmax, and T½ values will also be estimated using a one-compartment PK model that accounts for repeated observations from each subject. Censored data will be handled by incorporating appropriate likelihood methods in the estimation procedure.

Similar exercises will be performed to estimate Cmax, Tmax, and T½ values following 14 days of daily dosing. Qualitative differences in PK curves between the once- and twice-daily dosing schedule, and between the single dose and daily dosing results, will be evaluated both graphically and through modeling exercises. Where reasonable, data collected from the same woman (i.e., following the initial dose and following 14 days of daily dosing) will be incorporated in these supporting analyses.

It is possible that the act of taking biopsy samples could influence systemic blood tenofovir concentrations at later time points. Although it is unlikely that the study size is sufficient to identify such effects, these potential processes will be explored, as follows: The median tenofovir blood concentration at time point t+1 will be estimated for each group of 7 women having a biopsy at the previous time point (t). This median value will be compared to the median blood concentration for all remaining women who have their biopsy after time t. For example, the median blood concentration at 2 hours will be computed for the 7 women who had their biopsy at the 1 hour time point. This will be compared to the median 2-hour blood concentration for the remaining 35 women who have their biopsy at 2 or more hours after initial dosing. These differences will be summarized for each of the 6 possible time point comparisons.

Covariates

The effects of certain baseline factors (e.g., age, ethnicity, BMI) on Cmax, Tmax, and T½ values will be evaluated using analysis of variance techniques. These analyses will be exploratory and descriptive in nature.

Substudy analysis

Exercises of the single-dose phase will be repeated for up to 10 women at the Pittsburgh site who continue to the third phase of the study. The exercise will explore within-subject effects of this sub-sample after the single-dose application at 8 time points including the additional time point of 12-hour following the single dose.

Interim Analysis.

An interim analysis of approximately first 21 participants (ideally 7 at each site) completing the single-dose phase of the trial measuring tenofovir in plasma, vaginal biopsies, and cervicovaginal fluid following the initial dose will be performed. Genital compartment and blood concentration values will be summarized and plotted across sampling time points. Likewise, adverse event summaries will be provided to the project team. However no formal statistical tests will be conducted, and therefore no stopping rules will be applied. But it is possible that the results of this interim analysis could lead to an adjustment in the sampling times for the second phase of the trial.

XI. MANAGEMENT OF INTERCURRENT EVENTS

A. Loss to Follow-up

If a participant fails to appear for a scheduled visit, at least three attempts to contact her should be made over the subsequent 30 days. These attempts should be documented in the participant's study file. The final attempt must be a certified letter with return-receipt requested to the participant, or an outreach attempt. A copy of this letter should be in her file. After these three attempts, no further efforts need be made to find her, but her file should remain open until study closeout.

If the participant does not return to the clinic before the study is closed, the Final Disposition (FINAL) Form will be completed at the time of study close-out. The form should indicate that the participant was lost to follow-up. The "lost to follow-up" designation cannot be made for any participant until the closing date of the study.

B. Protocol Adherence

Participants in the two-week phase will be considered compliant with the study regimen if they have used at least 80% of scheduled doses (at least 11 out of 14 of the scheduled doses in the once-daily group and at least 21 out of 27 in the twice-daily group) and as long as they have used at least two doses prior to PK sampling. The study site should carefully record the date and time of each collection of PK blood and cervicovaginal sampling. The time of sampling will be considered a protocol deviation if it falls ± 15 minutes outside of the 30 minute and 1 hour time-points, ± 30 minutes outside of the 2, 4, 6 or 8 hour time-points or ± 2 hours outside of the 12 or 24 hour time-points.

C. Adverse Events

An adverse event or adverse experience (AE) is any untoward medical occurrence in a clinical investigation subject administered a pharmaceutical product and which does not necessarily have a causal relationship with this treatment. An AE can therefore be any unfavorable and unintended sign (including an abnormal laboratory finding), symptom, or disease temporally associated with the use of an investigational product, whether or not considered related to the investigational product. Pre-existing events that increase in frequency or severity in nature during or as a consequence of use of a drug in human clinical trials will also be considered as adverse events. Any medical condition or clinically significant laboratory abnormality with an onset date before the first date of study drug administration is considered to be pre-existing, and should be documented in the source document for the participant.

According to 21 CFR 312.32 "IND Safety Reports," the following definitions of terms apply to adverse events occurring in clinical studies involving drugs.

"Associated with the use of the drug" means: "There is a reasonable possibility that the experience may have been caused by the drug."

"Serious adverse drug experience" (SAE) means: "Any adverse drug experience occurring at any dose that results in any of the following outcomes: Death, a life-threatening adverse drug experience, inpatient hospitalization or prolongation of existing hospitalization, a persistent or significant disability/incapacity, or a congenital anomaly/birth defect. Important medical events that may not result in death, be life-threatening, or require hospitalization may be considered a serious adverse drug experience when, based upon appropriate medical judgment, they may jeopardize the patient or subject and may require medical or surgical intervention to prevent one of the outcomes listed in this definition."

"Unexpected adverse drug experience" means: "Any adverse drug experience, the specificity or severity of which is not consistent with the current investigator brochure; or, if an investigator brochure is not required or available, the specificity or severity of which is not consistent with the risk information described in the general investigational plan or elsewhere in the current application, as amended."

Any adverse events that are serious (whether expected or unexpected) and possibly associated with use of the drug must be immediately reported to CONRAD by telephone (21 CFR 312.64). If there is any question whether the adverse event meets any of these criteria, it should be reported by telephone anyway. In addition, a completed Adverse Event (AE) Form must be faxed to CONRAD as soon as possible.

CONRAD will notify FHI (the IND holder) who will notify the FDA by telephone or fax of any unexpected serious AEs associated with the use of the drug as soon as possible, but no later than 7 calendar days after initial receipt of the information from the investigator. In addition, FHI will notify all participating investigators and the FDA in writing. The written notification will be made as soon as possible and in no event later than 15 calendar days after initial receipt of the information.

Any serious adverse event that is associated with treatment and is unexpected must be reported promptly to the Institutional Review Board.

CONRAD will follow all serious adverse events with the cooperation of the investigator.

Adverse events that do not fall into the categories of associated with drug, serious, and unexpected may include illnesses, discomfort, pain, or any medical problem such as cold symptoms or other complaints. All adverse events, whether serious or non-serious and whether or not related to the study drug, should be recorded on the Adverse Event (AE) Form.

Each adverse event should be graded for severity using the DAIDS Table for Grading the Severity of Adverse Events (Appendix VI). **Note:** In Appendix IV, the table of Lab Normal Values indicates any modifications by CONRAD to DAIDS' normal accepted values;

For each adverse event, an assessment of the relatedness to the test agent should be made using the following scale:

- Unrelated: Onset of the AE had no reasonable temporal relationship to administration of the study product or a causal relationship to administration of the study product is biologically implausible or the event is attributed to an alternative etiology.
- Possibly Related: Onset of the AE has a reasonable temporal relationship to study product administration and a causal relationship is not biologically implausible.
- Probably Related: Onset of the AE has a strong temporal relationship to administration of the study product that cannot be explained by the subject's clinical state or other factors and a causal relationship is not biologically implausible.
- Definitely Related: Onset of the AE shows a distinct temporal relationship to administration of the study product that cannot be explained by the subject's clinical state or other factors or the AE occurs on rechallenge or the AE is a known reaction to the product or chemical group or can be predicted by the product's pharmacology.

The study agents that must be used in determining relationships of AEs are tenofovir gel and the study gel delivery applicator.

D. Management/Replacement of Participants Who Discontinue

Participants who discontinue the study may not re-enroll and will not be replaced.

E. Pregnancy

If a pregnancy is diagnosed, the site should notify CONRAD as soon as possible by telephone. The course of the pregnancy should be followed until it has an outcome. If the subject seeks care outside the site, every effort should be made to obtain her consent for the site to receive a copy of her medical records related to the pregnancy, its outcome and the health of the neonate, if applicable. The participant will be discontinued from the study and exit procedures followed.

F. Protocol Violations

If a protocol violation is required to protect the life or physical well being of a participant in an emergency, it may be carried out without prior approval from CONRAD or the IRB. The investigator must, however, report the violation to CONRAD and the IRB as soon as possible, no later than five working days after the emergency occurred. CONRAD will notify the FDA if indicated. The investigator will also keep a record of all such violations, with documents showing the dates of and reasons for each violation.

Other violations from the protocol may not be carried out without prior approval from the IRB if the change involves the rights, safety, or welfare of participants and without prior approval of the FDA if the change involves the study's scientific soundness or the rights, safety, or welfare of participants.

If an inadvertent protocol violation has occurred, CONRAD should be notified immediately to determine what steps should be taken.

Protocol violations may be documented on Protocol Violation Forms (PVF), a generic form developed by FHI for any study in which it manages data.

G. Modification of Protocol

No modification of this protocol may be made without the approval of CONRAD.

XII. REPORTING REQUIREMENTS

A. Study Initiation

At study initiation, the following forms and records will be required:

- Signed Statement of Investigator (Form FDA-1572)
- Curriculum vitae for each investigator listed on the Statement of Investigator
- Financial Disclosure Statement (completed by each investigator listed on the Statement of Investigator)
- Signed and dated protocol
- IRB information consisting of:
    - Name, address, and chairperson of the IRB
    - FWA number
    - List of IRB members (names may be withheld in accordance with IRB policy) with titles, genders, occupations and affiliations
    - Copy of IRB approval letter for protocol, consent form, and advertisement

    - Copy of IRB-approved consent form and advertisements used for recruiting
- Laboratory information including:
    - Name of laboratories to be used to process study specimens
    - CV of laboratory director(s)
    - Copy of laboratory licenses with expiration dates
    - Copy of normal values for tests done by each laboratory for this study

B. Study Conduct

During the study, a monthly report of participants screened, enrolled, completing each visit, and discontinued, as well as any adverse events related to product use will be sent to CONRAD.

The following forms and records will be maintained at the study site:

- Subject Screening Log
- Subject Enrollment Log
- Site Signature and Delegation Log
- Visit Log
- Study Supplies Inventory Logs
- Protocol Deviation Log
- Source documents
- Case Record Forms (CRFs)
- IRB documents:
    - Approval letters of protocol and any protocol amendments
    - Approval letters for original and any revised consent forms
    - Annual approval letter
    - Other IRB correspondence
- Updates on CVs and laboratory information
- Current Investigator Brochure
- IND Safety Reports
- General correspondence

Annual progress reports and a final report will be submitted by the site to the IRB. Copies of all documents submitted to the IRB should be sent to CONRAD.

XIII. MONITORING

A member of the CONRAD staff will monitor the site approximately every 6 to 8 weeks and/or on an as-needed basis. All clinical records and case report forms for the participants enrolled in this study will be made available for review by authorized individuals. A Visit Log will be maintained at the site in which all site monitoring visits will be recorded.

XIV. USE OF INFORMATION AND PUBLISHING

All information concerning the drug supplied by CONRAD to the investigator and not previously published is considered confidential.

No data collected in this study will be published without prior written approval from CONRAD.

XV. ANTICIPATED LENGTH OF STUDY

Recruitment for the study is expected to take 6-8 months. Each subject's participation will last approximately 4-6 months, making the clinical portion of the study last about 12 months. Data analysis will take 3 months and the Final Report an additional month.

XVI. CASE REPORT FORMS (CRFs)

All data will be recorded on 3-ply CRFs supplied by CONRAD. Each CRF must be initialed by the study staff member who completed it. The principal investigator, however, is responsible for the accuracy of data entered on the CRF and will sign a statement on the discontinuation form for each participant stating that he or she has reviewed all CRFs for that participant and certifies that all information on them is correct.

The source documents including signed informed consent forms, laboratory reports, patient records, and participant diary cards should be maintained in the participant's medical chart or study file, and should be available for review during monitoring visits.

The original and yellow copies of each CRF will be submitted to FHI after review by the CONRAD Clinical Research Monitor. The pink copy will be retained in the participant's clinic study file. It is imperative that the original and all copies match in every detail. Before a CRF is separated, corrections may be made on the CRF itself, so that they are recorded identically for all three copies. The person making the change must date and initial each change. Once the copies of a CRF are separated, however, changes may not be made on the copies themselves; they must be recorded on paper data discrepancy report forms. Once the correction has been made and the discrepancy form has been signed, a copy of the form should then be attached to the relevant CRF at the site, and a copy should be submitted to FHI. Use of opaque correction fluid is not permitted for <u>any</u> corrections to CRFs at any time.

All data queries sent to the investigator not in conjunction with a monitoring visit should be answered by the investigator in a timely fashion. The data relevant to the query will be updated based on the investigators' written response. Investigators must keep copies of all queries stapled to the CRFs in the participant's study file.

A detailed data management plan will be written before the data is collected for this study.

XVII. RECORDS RETENTION

Original and yellow copies of all CRFs will be kept at FHI. Photocopies of CRFs and pertinent study documentation (such as IRB correspondence, participant master list, etc.) will be kept by CONRAD.

The signed original informed consent documents for each participant, pink copies of CRFs, and originals of all study documentation (e.g. drug inventory forms, participant clinic records, original laboratory reports, diaries, etc.) will be retained by the investigator for a minimum of two years after U.S. Food and Drug Administration approval or withdrawal of a New Drug Application (NDA). If an NDA is not submitted within six years of the last follow-up visit, the center may request permission in

writing from CONRAD to destroy the records. No records may be destroyed without written permission from CONRAD.

The investigator may be subject to a field audit by FDA inspectors to validate the participation of study participants and to verify the data reported on the Case Report Forms. This audit could occur while the study is in progress, several years after the study is completed, or when the data are under review by the FDA as part of the new drug approval (NDA) process. All of the participants' records and other study documentation must be filed and accessible on short notice (3-5 days) during the study and subsequent retention period.

XVIII. PROCEDURE FOR OBTAINING INFORMED CONSENT

A. Institutional Review Board (IRB) Review and Approval

Before initiating the study, the study must be approved in writing by the local IRB or Ethical Review Committee, in accordance with FDA regulations (21 CFR Part 56). The study will be conducted in accordance with all conditions of approval by the IRBs. The site will obtain written re-approval of the research by the IRB annually.

B. Informed Consent

No participant may be admitted into this study until the investigator has obtained her legally effective informed consent. An investigator shall seek such consent only under circumstances that provide the prospective participant with sufficient opportunity to consider whether or not to participate in the study. Informed consent must be obtained without coercion, undue influence, or misrepresentation of the potential benefits or risks that might be associated with participation in the study.

Informed consent encompasses all oral or written information given to the participant about the study and the study materials. This includes the consent form signed by the participant, the instructions for use of study materials that are provided to the participant, recruitment advertising, and any other information provided to the participant. All such information that is given to the participant will be in a language that is understandable to her. The information will not include any language in which the participant is made to waive any of her rights or which releases or appears to release the investigator, the investigator's institution, or CONRAD from liability for negligence.

Informed consent will be documented by the use of a written consent form that is signed by the participant and the investigator (or designee). A copy of the signed consent form will be given to each participant. The original signed consent form for each participant will be kept in the participant's study file. The consent form must include each of the basic and additional elements of informed consent described in 21 CFR Part 50.25 and must describe each of the risks or discomforts to the participant that have been identified by CONRAD as reasonably foreseeable. CONRAD will provide a sample consent form that meets these requirements (Appendix VII). If the investigator revises the sample consent form or develops a new one, the new or revised consent form must be submitted to CONRAD for review at least one week before it is submitted to the local IRB.

C. Subject Confidentiality

The confidentiality of all subjects enrolled into this study will be protected to the fullest extent possible. Subjects' clinic records may be audited by CONRAD staff, U.S. FDA personnel, or other individuals authorized in writing by CONRAD to audit the study. However, study subjects should not be identified by name on any Case Record Form or on any other documentation sent to CONRAD and will not be reported by name in any report or publication resulting from data collected in this study.

XIX. ADDITIONAL INVESTIGATOR RESPONSIBILITIES

In addition to the previously described responsibilities, the principal investigator is responsible for signing and dating the cover page of this study protocol. The signed and dated original must be submitted to CONRAD, and a copy must be maintained by the principal investigator at the site with the study files.

All protocol amendments must be signed and dated by the principal investigator. The signed and dated original must be submitted to CONRAD, and a copy must be maintained by the principal investigator at the site. Amendments must be approved by CONRAD and the IRB before implementation.

The principal investigator will provide CONRAD with a Curriculum Vitae (CV) for herself/himself showing the education, training, and experience that qualifies her or him as an expert in the area of clinical investigation specific to the product under investigation and her/his affiliation with the site at which the study is being conducted. CVs also must be provided for all individuals listed as sub-investigators on the 1572 form showing the education, training, and experience that qualify them for their role in the study, and their affiliation with the study site.

The principal investigator will supply CONRAD with copies of the lab director's CV, current license and/or laboratory certification (such as the Clinical Laboratory Improvement Act [CLIA] certification) of any laboratory used for the study. The principal investigator is responsible for obtaining any updates to these documents and sending them to CONRAD in a timely fashion. This includes documentation of the normal ranges of the laboratory tests used by the laboratory. The principal investigator will ensure that appropriate health care or referral is provided for the study participants throughout the study. Copies of all these documents must be maintained at the site.

The principal investigator will follow guidelines set forth in the Declaration of Helsinki and will follow U.S. Department of Health and Human Services regulations regarding the Health Information Portability and Accountability Act (HIPAA –45, CFR 164). In addition, the principal investigator should follow Good Clinical Practice (GCP) guidelines.

Throughout the course of the study, the principal investigator will prepare and submit to CONRAD whatever reports are required and detailed in the Subcontract Agreement to be signed prior to initiating the study. These reports summarize the accomplishments of the assignment and usually include the following:

- The total number of participants admitted;
- The number of and reasons for discontinuations due to adverse events;

- A description of all serious adverse events;
- The number of participants completing the study;
- All changes in the research activity; and
- All unanticipated problems involving risks to participants or others.

The final technical report will be submitted within 45 days after completion of the study unless this period is extended in writing by the CONRAD Clinical Director.

XX. REFERENCES

1. Gilead Sciences Inc. (2005). Investigator's Brochure: Tenofovir Gel (GS-1278), Second Edition, 31 March 2005.

2. Gupta P, Collins KB, Ratner D et al. Memory CD4+ T Cells Are the Earliest Detectable Human Immunodeficiency Virus Type 1 (HIV-1)-Infected Cells in the Female Genital Mucosal Tissue during HIV-1 Transmission in an Organ Culture System. J of Virology 2002;76 (19): 9868-76.

3. Mayer KH, Maslankowski L, El-Sadr W, Justman J, Kwiecien A, Masse B, Eshleman SH, Hendrix C, Morrow K, Rooney JF, , Soto-Torres L; HPTN 050 Protocol Team. Safety and tolerability of tenofovir vaginal gel in abstinent and sexually active HIV-infected and uninfected women AIDS 2006; 20(4):543-51.

4. Patricia Barditch-Crovo, Steven G. Deeks, Ann Collier, Sharon Safrin, Dion F. Coakley, Michael Miller, Brian P. Kearney, Rebecca L. Coleman, Patrick D. Lamy, James O. Kahn, Ian McGowan, Paul S. Lietman. Phase I/II Trial of the Pharmacokinetics, Safety, and Antiretroviral Activity of Tenofovir Disoproxil Fumarate in Human Immunodeficiency Virus-Infected Adults. Antimicrob Agents Chemother 2001; 45 (10): 2733-2739.

5. Robbins et al. Anti-human immunodeficient virus activity and cellular metabolism of a potential prodrug of the acyclic nucleoside phosphonate 9-R-(2-phosphonomethoxyoxypropyl) adenine (PMPA), bis(isopropyloxymethylcarbonyl) PMPA. Antimic. Agents Chemother. 42: 612-617, 1998]

APPENDIX I: STUDY FLOW CHART

Visit Type	Visit 1: Screening	Visit 2: Enrollment/ Single-dose	Visit 3: 2 week Phase	Visit 4: 1 week f/u	Visit 5: 2 week f/u	Visit 6: Final visit
Informed consent, medical history/demographic information, HIV counseling	✓					
Urine pregnancy test	✓	✓	✓	✓	✓	✓
Dipstick urinalysis (microscopy and culture as indicated)	✓	(✓)	(✓)	(✓)	(✓)	(✓)
Blood collected for CBC, biochemistries	✓				✓	
Height and weight measured	✓					
HIV and HepBsAg testing	✓					
Wet mount/pH/NG/CT	✓	(✓)	(✓)	(✓)	(✓)	(✓)
Screening bimanual pelvic examination	✓	(✓)	(✓)	(✓)	(✓)	(✓)
Testing for HSV or Syphilis	(✓)	(✓)	(✓)	(✓)	(✓)	(✓)
Gynecological examination with visual inspection	✓	✓	✓	✓	✓	✓
Enrolled and randomized to one of seven biopsy time-points		✓				
Baseline plasma tenofovir levels		✓	✓			
Apply initial dose (in-clinic)		✓				
Apply morning dose (in-clinic)				✓	✓	
Plasma tenofovir levels at 0.5, 1, 2, 4, 6, 8, & 24 hour(s) after product application		✓			✓	
Vaginal biopsy		✓			✓	
Pelvic exam procedures (aspirate, endocervical specimen)		✓		✓	✓	
Given Participant Guidebook – Main Study		✓				
Randomized to once or twice-daily dosing			✓			
Given Diary Card instructions and study product			✓			
Randomized to one of three biopsy time-points (4, 8 or 24 hours)					✓	
Plasma tenofovir trough				✓	✓	
Given Participant Guidebook – Substudy**					✓	

*Parenthesis indicates that the procedure will take place if necessary.
** Substudy participants only

STUDY FLOW CHART: SUBSTUDY

Visit Type	Substudy Visit 1	Substudy Visit 2	Substudy Visit 3
Urine pregnancy test	✓	✓	✓
Dipstick urinalysis (microscopy and culture as indicated)	(✓)	(✓)	(✓)
Wet mount/pH/NG/CT	(✓)	(✓)	(✓)
Screening bimanual pelvic examination	(✓)	(✓)	(✓)
Testing for HSV or Syphilis	(✓)	(✓)	(✓)
Gynecological examination with visual inspection	✓	✓	✓
Baseline plasma tenofovir levels	✓		
Apply single dose at home	✓		
Plasma tenofovir levels at 12h after product application		✓	
Vaginal biopsy at 12h after product application		✓	
Pelvic exam procedures (aspirate, endocervical specimen) at 12h after product application		✓	

*Parenthesis indicates that the procedure will take place if necessary.

TABLE OF CONTENTS

	page
Study Contact Information	2
Instructions for Inserting Tenofovir Gel	3
Sample Diary Card (front and back)	4
How to fill out your Diary Card (ONCE-daily dosing)	5
How to fill out your Diary Card (TWICE-daily dosing)	6
Post-Biopsy Instructions	7
After Visit 2 - part 1 (Enrollment Visit)	8
After Visit 2 - part 2 (Enrollment Visit)	9
After Visit 3 (Beginning of Phase 2)	10
After Visit 4 (One-Week Follow Up)	11
After Visit 5 - part 1 (Two-Week Follow Up)	12
After Visit 5 - part 2 (Two-Week Follow Up)	13

STUDY CONTACT INFORMATION

CALL US ANYTIME YOU HAVE QUESTIONS OR CONCERNS

Contact: ____

Phone: ____

IF YOU HAVE A MEDICAL EMERGENCY

- Seek emergency care immediately.
- Ask your emergency provider to contact the study clinician at the phone number listed above.

INSTRUCTIONS FOR INSERTING TENOFOVIR GEL

1. Take one applicator and one tube of tenofovir gel out of participant kit. (NOTE: If the study products appear to have been tampered with, do not use them and call the research staff immediately.)

2. Unscrew the white plastic cap off the gel tube. Turn the cap upside down and use the point in the middle to pierce the foil seal of the gel tube.

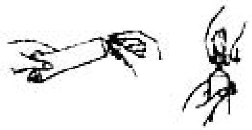

3. Make sure the plunger is pushed all the way in. Attach the applicator to the tube by screwing it onto the threaded end.

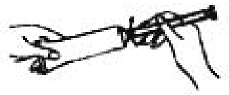

4. Starting from the bottom of the tube, squeeze the tenofovir gel out of the tube until the plunger is fully extended (it will not come all the way out).

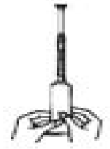

5. Unscrew the applicator from the tube and set the used tube aside.

6. Stand, sit, or lie down in whatever position you find most comfortable for inserting tampons or other vaginal products.

7. Hold the applicator in the middle and insert the open end of the applicator into your vagina until your fingers touch your vagina.

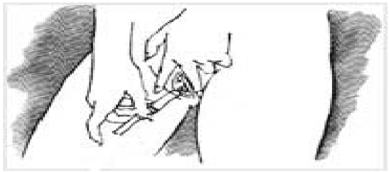

   If the applicator does not go in easily, change the angle of insertion, or lubricate the tip with a small amount of tenofovir gel. Do not use any other lubricant. Do not push the applicator in against resistance.

8. Once the applicator is inserted, push the plunger all the way in to dispense the gel.

9. Remove the applicator and throw it away.

10. Put the white plastic cap back on the tube. Put the tube in the zip-lock bag provided for <u>used</u> tubes.

11. Please remember to bring your entire box of study supplies including all used and unused gel tubes to your next visit.

SAMPLE DIARY CARD (FRONT)

TITLE: Single Dose and 14-Day Once or Twice-daily Pharmacokinetic Study of the Vaginal Microbicide Agent 1% Tenofovir Gel (CONRAD Protocol A04-095)

PARTICIPANT NUMBER: __________

DATE AND TIME OF GEL INSERTION

INSTRUCTIONS:

1. Complete one row for each day you are in the study. For each day, write the time that you insert the study gel.
2. If you have symptoms or problems on any day, record them on the *Symptoms and Medication* form, on the back.

**Date** (MM/DD/YY) (A day is: midnight to 11:59 PM)	**Time that gel was inserted** *Circle AM or PM for each.* Note: Depending on your assigned group, you should only insert the gel once or twice a day. The third column below is in case you insert an evening dose after midnight.			**Comments**
01/14/07	9 : 00 AM	9 : 05 PM	___ : ___ AM PM	
01/15/07	8 : 55 AM	9 : 02 PM	___ : ___ AM PM	
01/16/07	9 : 00 AM	___ : ___ AM PM	___ : ___ AM PM	
01/17/07	12 : 01 AM	9 : 04 AM	9 : 00 PM	
01/18/07	9 : 01 AM	9 : 07 PM	___ : ___ AM PM	itchy (labia minora)
01/19/07	9 : 00 AM	9 : 04 PM	___ : ___ AM PM	
01/20/07	9 : 05 AM	9 : 00 PM	___ : ___ AM PM	
/ /	AM	AM	AM	

SAMPLE DIARY CARD (BACK)

SYMPTOMS AND MEDICATION

INSTRUCTIONS:

1. If you have any symptoms that occur during the study, please record them below.
2. If you take any medication to treat your symptoms, please record the type of medication below.

Please call the Study Coordinator if you experience any moderate or severe symptoms (as defined at the bottom of the page) including vaginal burning, itching, pain, spotting or bleeding at any time during product usage.

**Symptom**	**Start date (MM/DD/YY)**	**End date (MM/DD/YY)**	**Duration (if it lasts less than 24 hrs)**	**Severity***	**Type of medication**
itchy (labia minora)	01/18/07	01/18/07	Hr: 1 Min: 30	1	none
	___/___/___	___/___/___	Hr: _____ Min: _____		
	___/___/___	___/___/___	Hr: _____ Min: _____		

HOW TO FILL OUT YOUR DIARY CARD (<u>ONCE</u>-DAILY DOSING)

- Each reporting day begins at 12:00am (midnight) and ends at 11:59pm. Please insert the tenofovir gel in the morning, at approximately the same time each morning.

- Fill out one row – in blue or black ink – each day (every day) that you use the gel. You will fill out only the first AM/PM box as you will only insert the tenofovir gel once a day. Always write the date and time you <u>actually</u> insert the gel. If you forget to insert the gel at your normal time, even if you forget until the next day, insert it as soon as your remember. HOWEVER, if you remember when there is only 6 hours until you are supposed to insert the next dose, skip the forgotten dose and continue with your regular schedule. If you miss two or more doses, contact the clinic immediately to let them know.

- Please use the Symptoms and Medication section (on the back of your Diary Card) to record any medications you use while you are in the study. Also, you will record in this section your experience with tenofovir gel use. This includes any itching, burning, irritation, pain, discharge, etc. you experience when using the gel.

- When you fill out the **Symptom** column, please BRIEFLY describe the symptom and your experience. When reporting on possible symptoms in your genital area, please refer to Figure A below to explain <u>where</u> you experience the symptom. **All of your comments are very important to us.**

- If you experience any vaginal spotting or bleeding or have any moderate symptoms (where you cannot perform some of your normal activities) or severe symptoms (where you cannot perform most or any of your normal activities), please call the study clinic <u>immediately</u> to schedule a visit. If it is an emergency, seek emergency care immediately, and as your emergency care provider to contact the clinic.

- We will review your Diary Card with you at each visit. If you have any questions at any other time, please feel free to call the study clinic.

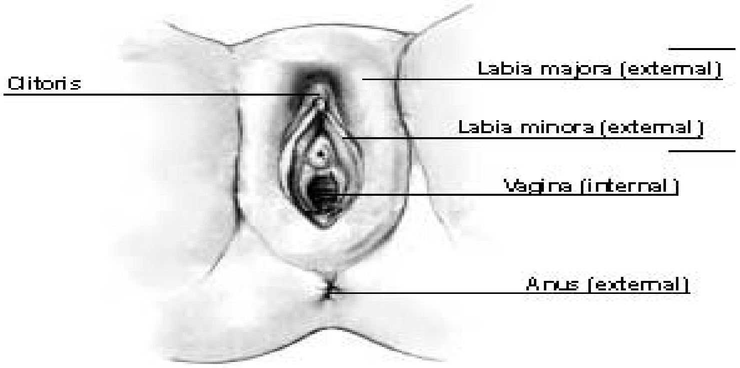

Figure A

HOW TO FILL OUT YOUR DIARY CARD (TWICE-DAILY DOSING)

- ❖ Each reporting day begins at 12:00am (midnight) and ends at 11:59pm. Please insert the tenofovir gel once in the morning and once in the evening, at approximately the same times each day.

- ❖ Fill out one row – in blue or black ink – each day (every day) that you use the gel. You will fill out two AM/PM columns each day, one for the morning dose and one for the evening dose. The third column is in case you insert an evening dose after midnight. (Example: Let's say you usually insert the gel at 9am and 9pm every day. On 7/15/06, you insert the morning dose at 9am, so you fill in the first column. Then you forget to insert your evening dose until 12:01am (after midnight). Now you'd go to the next line for 7/16/06 and fill in 12:01am in the first column, 9am in the second column and 9pm in the third column.) REMEMBER: always write the date and time you actually inserted the gel. If you forget to insert the gel at your normal time, even if you forget until the next day, insert it as soon as your remember. HOWEVER, if you remember when there is only 6 hours until you are supposed to insert it the next time, skip the forgotten dose and continue with your regular schedule. If you miss two or more doses, contact the clinic immediately to let them know.

- ❖ Please use the Symptoms and Medication section (on the back of your Diary Card) to record any medications you use while you are in the study. Also, you will record in this section your experience with tenofovir gel use. This includes any itching, burning, irritation, pain, discharge, etc you experience when using the gel.

- ❖ When you fill out the **Symptom** column, please BRIEFLY describe the symptom and your experience. When reporting on possible symptoms in your genital area, please refer to Figure A below to explain where you experience the symptom. **All of your comments are very important to us.**

- ❖ If you experience any moderate symptoms (where you cannot perform some of your normal activities) or severe symptoms (where you cannot perform most or any of your normal activities) including vaginal burning, itching, pain, spotting or bleeding, please call the study clinic immediately to schedule a visit. If it is an emergency, seek emergency care immediately, and as your emergency care provider to contact the clinic.

- ❖ We will review your Diary Card with you at each visit. If you have any questions at any other time, please feel free to call the study clinic.

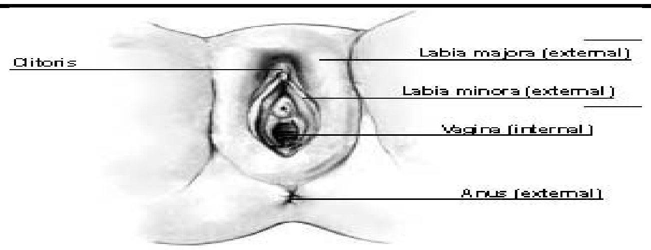

Figure A

POST-BIOPSY INSTRUCTIONS

- For ONE WEEK following the biopsy, do not:
    - Douche
    - Masturbate
    - Use tampons
    - Have vaginal or anal sex
    - Put anything in your vagina, including fingers, sex toys or any other objects

- After your vaginal biopsy, feel free to rest for a few minutes before going home.

- It is normal to have some mild pain or spotting for several days after your procedure. Dark/black-colored discharge may also be normal if you had medication applied to your vagina to control bleeding. You may wear a sanitary pad for any bleeding.

- You may take a pain reliever for mild pain as recommended by your study clinician. Be sure to take only recommended medications.

- You may immediately resume your normal diet.

- Notify your study clinician immediately if you have any of the following:
    - Bleeding (if more than spotting)
    - Foul-smelling discharge from your vagina
    - Fever and/or chills
    - Severe lower abdominal pain

- Your study clinician may give you additional or alternate instructions after the procedure, depending on your particular situation.

AFTER VISIT 2 (ENROLLMENT VISIT - part 1)

- Today, you completed the first part of your **Enrollment Visit**.
- The second and last part of the Enrollment visit, will be tomorrow**.**

Date: ____ Time:__________________

- Between today and tomorrow:
  - For at least ONE WEEK after this visit, follow **Post-biopsy Instructions.**
  - During this time, please do **not**:
    - Have vaginal or anal sex
    - Masturbate
    - Receive oral sex
    - Douche
    - Put anything in your vagina, including fingers, tampons, sex toys or other objects.
    - Use any vaginal hygiene products, lubricants, spermicides, diaphragms or cervical caps
- Tomorrow, you will:
  - Be asked if you had any health problems since your last visit
  - Provide your 24 hour blood draw
  - Provide your 24 hour fluids and biopsy samples, if you were assigned to that group
- **Bring this guidebook with you every time you come to the clinic.**

AFTER VISIT 2 (ENROLLMENT VISIT - part 2)

- Today, you completed the last part of Visit 2.
- Your next visit, **Visit 3,** will begin the two-week phase and should be scheduled at least one month from today when you are not expected to menstruate for at least the next 15 days:

Date: ______________________________ Time:______________

- Between today and your next visit:
  - You will have about a one month "wash-out" period
  - For at least ONE WEEK after this visit, follow **Post-biopsy Instructions.**
  - You may continue normal sexual activity from the period that lasts from one week after this visit, until 72 hours before your next visit, unless you are experiencing severe abdominal pain, bleeding (if more than spotting), foul-smelling discharge, or fever and/or chills.
  - Starting 72 hours before your next visit, please do **not**:
    - Have vaginal or anal sex
    - Masturbate
    - Receive oral sex
    - Douche
    - Put anything in your vagina, including fingers, tampons, sex toys or other objects.
    - Use any vaginal hygiene products, lubricants, spermicides, diaphragms or cervical caps
- At your next visit, you will:
  - Give a urine specimen
  - Be asked if you had any health problems since your last visit
  - Have a pelvic exam
  - Be assigned to use the tenofovir gel either once or twice daily for two (2) weeks
  - Receive your kits that include applicators and tenofovir gel
  - Receive a Diary Card and instructions.
- **Bring this guidebook with you each time you come to the clinic.**

AFTER VISIT 3 (BEGINNING OF PHASE 2)

- Today, you completed **Visit 3,** and started the second study phase.

- Your next visit will be your **One-week Follow Up** (in one week):

  Date: ____ Time:__________________

- Between today and your next visit:

  - Depending on which group you were assigned, follow the appropriate instructions to insert tenofovir gel ___________ a day, every day at about the same time(s).
  - Some instructions to keep in mind:
    - Empty your bladder and bowels before you insert each dose and try not to empty them again for at least one hour after you insert each dose.
    - Walk around for at least 15 minutes after each dose
    - If you miss a dose, insert it as soon as you remember and then resume your regular schedule. However, if you remember when it is 6 hours or less before your next scheduled dose, then skip the forgotten dose and resume your regular schedule.
    - If you miss two or more doses, call the study site for further instructions.
  - After you insert the gel each time, fill out your Diary Card with the information required. Also record any symptoms and medications on the back of the Diary Card.

  - Until the end of this study phase, please do **not**:
    - Have vaginal or anal sex
    - Masturbate
    - Receive oral sex
    - Douche
    - Put anything in your vagina, including fingers, tampons, sex toys or other objects.
    - Use any vaginal products other than the tenofovir gel including vaginal hygiene products, lubricants, spermicides, diaphragms or cervical caps

  - **On the morning of your next appointment, do NOT insert your morning dose until you are at the clinic and are instructed to insert it. It is important to remember to bring your entire box of study supplies to the clinic with you, including used gel tubes.**

- At your next visit, you will:
  - Give a urine specimen
  - Review your Diary Card with the clinic staff and be asked if you had any health problems since your last visit
  - Return your used gel tubes from the past week.
  - Have a pelvic exam
  - Have a blood sample taken before you insert the morning dose of gel
  - Provide vaginal samples 4 hours after you insert the gel

Bring this guidebook <u>and your entire box of study supplies</u> with you to your next visit.

AFTER VISIT 4 (ONE-WEEK FOLLOW UP)

- Today, you completed Visit 4, your **One-week Follow Up**.
- Your next visit will be your **Two-week Follow Up** (in one week):

  Date: ____ Time:__________________

- Between today and your next visit:
  - Depending on which group you were assigned, continue using the tenofovir gel as often as you did last week, ___________ a day, every day.
  - After you insert the gel each time, fill out your Diary Card with the information required. Also record any symptoms and medications on the back of the Diary Card.
  - Until the end of the study, please do **NOT**:
    - Have vaginal or anal sex
    - Masturbate
    - Receive oral sex
    - Douche
    - Put anything in your vagina, including fingers, tampons, sex toys or other objects.
    - Use any vaginal products other than the tenofovir gel including vaginal hygiene products, lubricants, spermicides, diaphragms or cervical caps
  - **The morning of your next appointment, do NOT insert your morning dose until you are at the clinic and are instructed to insert it. It is important to remember to bring your entire box of study supplies to the clinic with you, including all used gel tubes.**
- At your next visit, you will:
  - Give a urine specimen
  - Review your Diary Card with the clinic staff and be asked if you had any health problems since your last visit. Your Diary Card will be collected but you will be given a copy of the back of it, to record any symptoms and medications until the end of the study.
  - Return your used gel tubes from the past week.
  - Have a pelvic exam
  - Have a blood sample taken before you insert the final dose of gel
  - Stay at the clinic for 8 hours for repeated blood draws at different times
  - Provide vaginal fluids and biopsy samples if you were selected for the 4-hour or 8-hour groups
- **Bring this guidebook and your entire box of study supplies with you to your next visit.**

AFTER VISIT 5 (TWO-WEEK FOLLOW UP – part 1)

- Today, you completed most of Visit 5, your **Two-week Follow Up**.
- The second and last part of Visit 5, will be tomorrow:

  Date: ____ Time:___________________

- Between today and tomorrow:
  - For at least ONE WEEK after this visit, follow **Post-biopsy Instructions** if you provided biopsy samples for the 4-hour or 8-hour groups.
  - During this time, please do **NOT**:
    - Have vaginal or anal sex
    - Masturbate
    - Receive oral sex
    - Douche
    - Put anything in your vagina, including fingers, tampons, sex toys or other objects.
    - Use any vaginal hygiene products, lubricants, spermicides, diaphragms or cervical caps
- Tomorrow, you will:
  - Review the copy of your Symptoms and Medications form with the clinic staff and be asked if you had any health problems since your last visit.Provide your 24-hour blood drawProvide vaginal fluids and biopsy samples if you were selected for the 24-hour group
- **Bring this guidebook with you to your next visit.**

AFTER VISIT 5 (TWO-WEEK FOLLOW UP - part 2)

<u>If you are NOT participating in the substudy:</u>

- ❖ Today, you completed the last part of Visit 5**.**
- ❖ Your next visit will be Visit 6, your **Final Visit** (in one or two weeks)

Date: ____ Time:__________________

- ❖ Between today and your last visit:
  - For at least ONE WEEK after this visit, follow **Post-biopsy Instructions**.
  - During this time, please do **NOT**:
    - o Have vaginal or anal sex
    - o Masturbate
    - o Receive oral sex
    - o Douche
    - o Put anything in your vagina, including fingers, tampons, sex toys or other objects.
    - o Use any vaginal hygiene products, lubricants, spermicides, diaphragms or cervical caps
- ❖ At your last visit, you will:
  - Give a urine specimen
  - Review the copy of your Symptoms and Medications form with the clinic staff and be asked if you had any health problems since your last visit. The copy of your Symptoms and Medications portion of your diary card will be collected at this visit.
  - Have a pelvic exam Be exited from the study – THANK YOU!
- ❖ **Bring this guidebook with you to your last visit.**

<u>If you ARE participating in the substudy:</u>

You will be given a Substudy Participant Guidebook today (Visit 5)

APPENDIX III: PARTICIPANT GUIDEBOOK: SUBSTUDY

TABLE OF CONTENTS

page

Study Contact Information	2
Instructions for Inserting Tenofovir Gel	3
Sample Diary Card (Substudy)	4
How to fill out your Diary Card	5
Post-Biopsy Instructions	6
After Phase 2 Final Visit	7
After Substudy Visit 1	8
After Substudy Visit 2	9

STUDY CONTACT INFORMATION

CALL US ANYTIME YOU HAVE QUESTIONS OR CONCERNS

Contact: ______Center for Family Planning Research_________________________

Phone: ____

IF YOU HAVE A MEDICAL EMERGENCY

- Seek emergency care immediately.
- Ask your emergency provider to contact the study clinician at the phone number listed above.

INSTRUCTIONS FOR INSERTING TENOFOVIR GEL

1. Take the applicator and tube of tenofovir gel out of your participant kit. (NOTE: If the study products appear to have been tampered with, do not use them and call the research staff immediately.)

2. Unscrew the white plastic cap off the gel tube. Turn the cap upside down and use the point in the middle to pierce the foil seal of the gel tube.

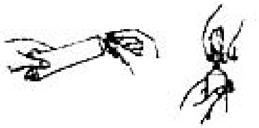

3. Make sure the plunger is pushed all the way in. Attach the applicator to the tube by screwing it onto the threaded end.

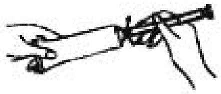

4. Starting from the bottom of the tube, squeeze the tenofovir gel out of the tube until the plunger is fully extended (it will not come all the way out).

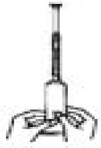

5. Unscrew the applicator from the tube and set the used tube aside.

6. Stand, sit, or lie down in whatever position you find most comfortable for inserting tampons or other vaginal products.

7. Hold the applicator in the middle and insert the open end of the applicator into your vagina until your fingers touch your vagina.

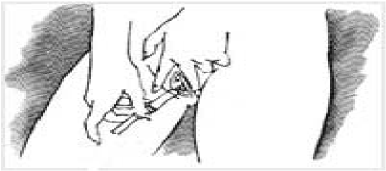

   If the applicator does not go in easily, change the angle of insertion, or lubricate the tip with a small amount of tenofovir gel. Do not use any other lubricant. Do not push the applicator in against resistance.

8. Once the applicator is inserted, push the plunger all the way in to dispense the gel.

9. Remove the applicator and throw it away.

10. Put the white plastic cap back on the tube. Put the tube in the zip-lock bag provided for used tubes.

11. Please remember to bring all study supplies, including your used gel tube, to your next appointment.

SAMPLE DIARY CARD (SUBSTUDY)

TITLE: Single Dose and 14-Day Once or Twice-daily Pharmacokinetic Study of the Vaginal Microbicide Agent 1% Tenofovir Gel (CONRAD Protocol A04-095)

PARTICIPANT NUMBER: __________

DATE AND TIME OF GEL INSERTION

INSTRUCTIONS:

1. The clinic will tell you what time to insert the gel in the evening on the same day of your Substudy Visit 1. This will be about 12 hours before your Substudy Visit 2. Record the date and time you inserted the gel.
2. If you have symptoms or problems, record them in the *Symptoms and Medication* section below.

Date (MM/DD/YY)	Time that gel was inserted	Comments
______/______/______	_______ : _______ PM	

SYMPTOMS AND MEDICATION

INSTRUCTIONS:

1. If you have any symptoms that occur during the study, please record them below.
2. If you take any medication to treat your symptoms, please record the type of medication below.

Please call the Study Coordinator if you experience any moderate or severe symptoms (as defined at the bottom of the page) including vaginal burning, itching, pain, spotting or bleeding at any time during product usage.

Symptom	Start date (MM/DD/YY)	End date (MM/DD/YY)	Duration (if it lasts less than 24 hrs)	Severity*	Type of medication
	____/____/____	____/____/____	Hr: ____ Min: ____		
	____/____/____	____/____/____	Hr: ____ Min: ____		
	____/____/____	____/____/____	Hr: ____ Min: ____		

***SEVERITY OF SYMPTOMS:**
1 = Mild (able to do all activities)
2 = Moderate (must discontinue some normal activities)
3 = Severe (unable to perform most normal activities)

SAMPLE

HOW TO FILL OUT YOUR DIARY CARD

- The reporting day begins at 12:00am (midnight) and ends at 11:59pm.
- Using blue or black ink, fill out the date and time that you actually inserted the gel in the evening. (This should be the same day you received the gel, and approximately 12 hours before your next scheduled appointment.) If you forget to insert the gel until after 2 hours after the assigned time, call the clinic immediately for further instructions.
- Please use the Symptoms and Medication section of your Substudy Diary Card to record any medications you use while you are in the study. Also, you will record in this section your experience with tenofovir gel use. This includes any itching, burning, irritation, pain, discharge, etc. you experience when using the gel.
- When you fill out the **Symptom** column, please BRIEFLY describe the symptom and your experience. When reporting on possible symptoms in your genital area, please refer to Figure A below to explain where you are experiencing the symptom. **All of your comments are very important to us.**
- If you experience moderate symptoms (where you cannot perform some of your normal activities) or severe symptoms (where you cannot perform most or any of your normal activities) including vaginal burning, itching, pain, spotting or bleeding at any time during product usage, please call the study clinic immediately to schedule a visit. If it is an emergency, seek emergency care immediately, and as your emergency care provider to contact the clinic.
- We will review your Diary Card with you at the next visit. If you have any questions at any other time, please feel free to call the study clinic.

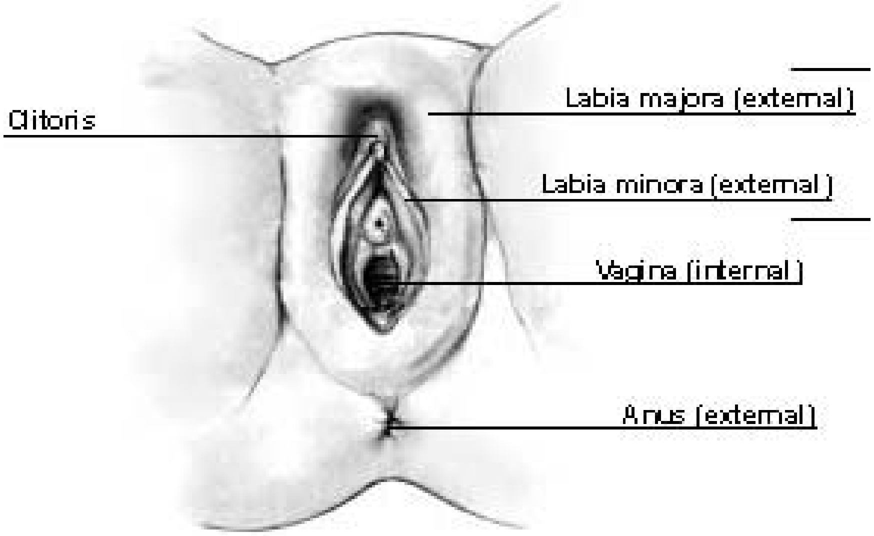

Figure A

POST-BIOPSY INSTRUCTIONS

- ❖ For ONE WEEK following the biopsy, do not:
  - Douche
  - Masturbate
  - Use tampons
  - Have vaginal or anal sex
  - Put anything in your vagina, including fingers, sex toys or any other objects

- ❖ After your vaginal biopsy, feel free to rest for a few minutes before going home.

- ❖ It is normal to have some mild pain or spotting for several days after your procedure. Dark/black-colored discharge may also be normal if you had medication applied to your vagina to control bleeding. You may wear a sanitary pad for any bleeding.

- ❖ You may take a pain reliever for mild pain as recommended by your study clinician. Be sure to take only recommended medications.

- ❖ You may immediately resume your normal diet

- ❖ Notify your study clinician immediately if you have any of the following:
  - Bleeding (if more than spotting)
  - Foul-smelling discharge from your vagina
  - Fever and/or chills
  - Severe lower abdominal pain

- ❖ Your study clinician may give you additional or alternate instructions after the procedure, depending on your particular situation.

AFTER VISIT 5 OF THE MAIN STUDY

- Today, you completed the main study. You have volunteered to participate in the Substudy.

- Your next visit, **Substudy Visit 1,** will begin the third and last phase and should be scheduled at least one month from today when you are not menstruating, ideally between days 19-24 of your cycle:

  Date: ____ Time:___________________

- Between today and your next visit:

  - You will have about a one month "wash-out" period

  - For at least ONE WEEK after this visit, follow **Post-biopsy Instructions.**

  - You may continue normal sexual activity from the period that lasts from one week after this visit, until 72 hours before your next visit, unless you are experiencing severe abdominal pain, bleeding (if more than spotting), foul-smelling discharge, or fever and/or chills.

  - Starting 72 hours before your next visit, please do **not**:

    - Have vaginal or anal sex
    - Masturbate
    - Receive oral sex
    - Douche
    - Put anything in your vagina, including fingers, tampons, sex toys or other objects.
    - Use any vaginal hygiene products, lubricants, spermicides, diaphragms or cervical caps

- At your next visit, you will:

  - Give a urine specimen
  - Be asked if you had any health problems since your last visit
  - Have a pelvic exam
  - Give a blood sample
  - Receive a single dose of tenofovir gel and one applicator
  - Receive a Substudy Diary Card and instructions.

- **Bring this guidebook with you to your next visit.**

AFTER SUBSTUDY VISIT 1

- ❖ Today, you completed Substudy Visit 1.
- ❖ Your next visit will be Substudy Visit 2 (tomorrow)

Date: ____ Time:__________________

- ❖ Between today and your next visit (tomorrow):
  - You will insert the single dose of tenofovir gel this evening at _________ PM (approximately 12 hours before your scheduled visit time tomorrow).
  - Some instructions to keep in mind:
    - ❖ Empty your bladder and bowels before you insert each dose and try not to empty them for at least one hour after you insert each dose.
    - ❖ Walk around for at least 15 minutes after each dose
  - After you insert the gel, fill out your Substudy Diary Card with the information required. Also record any symptoms and medications on the bottom portion of the Diary Card.
  - Until the end of the study, please do **not**:
    - o Have vaginal or anal sex
    - o Masturbate
    - o Receive oral sex
    - o Douche
    - o Put anything in your vagina, including fingers, tampons, sex toys or other objects.
    - o Use any vaginal products other than the tenofovir gel including vaginal hygiene products, lubricants, spermicides, diaphragms or cervical caps
- ❖ At your next visit, you will:
  - Give a urine specimen
  - Review your Diary Card with the clinic staff and be asked if you had any health problems since your last visit. Your diary card will be collected, but you will receive a copy of it to record any symptoms and medications until the end of the study.
  - Have a pelvic exam
  - Provide blood, vaginal, and biopsy samples 12 hours after your evening dose
- ❖ **Bring this guidebook <u>and your used gel tube</u> with you to your next visit.**

AFTER SUBSTUDY VISIT 2

- Today, you completed Substudy Visit 2.
- The last visit, Substudy Visit 3, will be in one or two weeks:

  Date: ____ Time:__________________

- Between today and your last visit:
  - For at least ONE WEEK after this visit, follow **Post-biopsy Instructions**.
- At your final visit, you will:
  - Provide a urine sample
  - Have a pelvic exam
  - Review any health problems you had since your last visit
  - Be exited from the study – THANK YOU!
- **Bring this guidebook with you to your last visit.**

APPENDIX IV: BLOOD PARAMETERS* AND NORMAL VALUE RANGES

I. Complete Blood Count (CBC) With Differential:
- White Blood Cell Count
- Hemoglobin
- Platelets
- Absolute Lymphocyte Count
- Absolute Neutrophil Count

II. Serum Chemistries
- Random Glucose
- Creatinine
- Sodium
- Potassium
- Calcium
- Alkaline Phosphatase
- Bilirubin (total)
- SGOT (AST)
- SGPT (ALT)
-

III Tenofovir levels (see appendix V)
- Blood Plasma assay

IV Antibody/Antigen testing
- HIV
- Hepatitis (HBsAg)

* A full CBC with differential or metabolic panel with additional blood parameter may be drawn depending on the laboratory panels available at the sites. However, only blood parameters indicated on this form will be used for entry criteria and for grading of adverse events.

Lab Normal Values for Serum Chemistries and Hematology

Note: The following units are equivalent: mm^3 = μL = cu mm = mcl

TEST	NORMAL VALUE	NOTE
WBC (thou/mcl)	2.6 – 16.0 †	2.6 thou/mcl = 2.6 x10^3/μL
Hemoglobin (g/dL)	11.0 – 18.0 †	Any decrease of at least 2.5 is considered an AE (see p.78 of the protocol for details).
Platelet (thou/mcl)	125 – 600 †	125 thou/mcl = 125,000/mm^3
Neutrophils (absolute/mcl)	1301 – 12000 †	1301/mcl = 1.3 x 10^3/ μL = 1301/cu mm
Lymphocytes (absolute/mcl)	651 – 6000 †	651/mcl = 0.651 x 10^3/ μL = 651/cu mm
Random non-fasting Glucose (mg/dL)	65-125	
Creatinine (mg/dL)	< 1.3 §	
Sodium (mmol/L)	135-150 ¤	mmol/L = meq/L
Potassium (mmol/L)	3.1 – 6.0 ¤	mmol/L = meq/L
Calcium (mg/dL)	8.1 – 11.5 ¤	
Bilirubin, Total (mg/dL)	< 1.4 §	
ALP (units/L)	< 145 §	Please round to whole number.*
SGOT (AST) (units/L)	< 49 §	Please round to whole number.*
SGPT (ALT) (units/L)	< 50 §	Please round to whole number.*

† As DAIDS does not define upper limits for these values, upper limits have been defined by CONRAD.

¤ To allow for mild variances in site ULNs, upper limits were increased to match the ULN of the “mild” category of DAIDS.

§ ULN values have been defined by CONRAD for greater consistency among the sites.

* 0.1 – 0.4 → round down; 0.5 – 0.9 → round up

APPENDIX V: LABORATORY PROCEDURES FOR PK ANALYSIS

***Blood plasma*:**

Blood for PK analysis will be collected at the appointed times. A Vacutainer Access Device which locks into a heparin lock may be used for the first seven blood collections baseline and (0.5, 1, 2, 4, 6, and 8 hours) after the single dose and after two weeks of dosing in order to facilitate sample collection. Approximately 0.5 mL of blood should be removed prior to drawing a sample into the vacutainer. This Vacutainer Access Device will be removed after the 8 hour draw and the 7th blood collection will be obtained at 24 hours. For each blood sample the following procedure applies:

- Collect 3 ml of whole blood in a purple top tube with K-EDTA.
- Within 30 minutes of collection (or up to 1 hour on ice) spin whole blood at 4º C @ 3000 RPM x 10 minutes
- Pipette 1mL of plasma into a labeled cryogenic vial[1]
- Cap vial and freeze at -80º C.
- The specimens will be stored at the site pending shipment in batches on dry ice to the central laboratory (UNC).

Cervicovaginal fluid:

Direct aspirates of cervicovaginal fluid pooling will be collected at the enrollment visit, one week follow-up visit and two week follow-up visit. For each cervicovaginal fluid sample the following procedure applies:

- Use vaginal aspirator syringes (2.5 mL) supplied by laboratory to collect direct aspirates.
- For maximum volume collection, the study subject should be supine for 15 minutes prior to collection.
- Place fluid into a labeled cryogenic vial.
- Cap vial and freeze at -80º C.
- Store at the site pending shipment in batches on dry ice to the central laboratory (UNC).

Cytobrush specimens:

Cytobrush specimens will be collected at the enrollment visit, the one week follow-up visit and the two week follow-up visit. These samples will be stored, frozen, and analyzed by Gilead if vaginal mononuclear cells are of a sufficient quantity to measure intracellular tenofovir diphosphate. For each cytobrush sample the following procedure applies:

- After sample collection, place cytobrush in 5 mL of phosphate-buffered saline (PBS) solution in a 15 mL falcon tube.
- Vigorously mix the tube and brush to remove all of the cells from the cytobrush.
- Remove the cytobrush from the tube and spin the falcon tube at 4º C @ 400 RCF x 15 minutes.

[1]The cryogenic vial used in this study will be: Corning Item Number 430658 (1.2 mL, self standing, silicone washer, external thread, polypropylene cryogenic vial)

- Carefully pour off or pipette and discard the supernatant, being careful not to disturb the cell pellet.
- Resuspend the pellet in 1 mL PBS with gentle agitation.
- Mix 10 uL of cell suspension sample with 10 uL of 0.4% Trypan Blue Stain in a small conical tube to count mononuclear cells. See box below for instructions.
- Spin the remainder of the 1 mL sample at 4º C @ 400 RCF x 15 minutes.
- Carefully pour or pipette off the supernatant and discard, being careful not to disturb the cell pellet.
- Add 1.0 mL of well-mixed, ice cold 70% methanol solution (prepared by separately measuring with graduated cylinders 70 mL of MeOH with 30 mL of H_2O) to the 15 mL falcon tube containing the pellet. Vortex lightly to lyse the cells.
- The specimens will be stored at -80 º C at the site pending shipment in batches on dry ice to the central laboratory (Gilead).

- Use a Cell Counting Chamber or hemocytometer to count the number of viable mononuclear cells per ml of suspension. Viable cells exclude Trypan Blue and remain clear under microscopy, while dead cells stain blue due to Trypan blue uptake. Mononuclear cells appear small and round under the microscope. Do not count red blood cells or epithelial cells which will appear larger with distinctive margins.

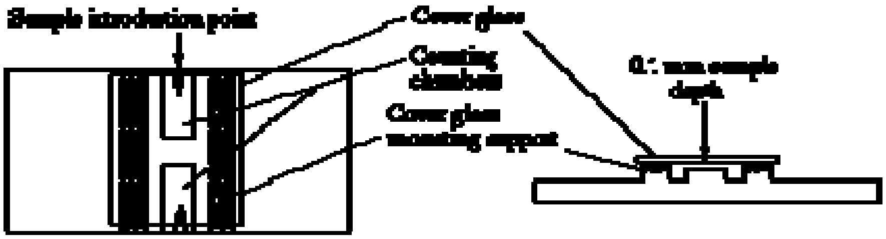

- To prepare the counting chamber, carefully clean the mirror-like polished surface and coverslip with lens paper. Place the coverslip over the counting surface prior to putting on the cell suspension.

- Introduce about 10 uL of the Trypan blue-cell mixture into one of the V-shaped wells with a pasteur pipette. The area under the coverslip will fill by capillary action. Introduce enough liquid so that the mirrored surface is just covered. Place the charged counting chamber on the microscope stage and focus the counting grid at low power.

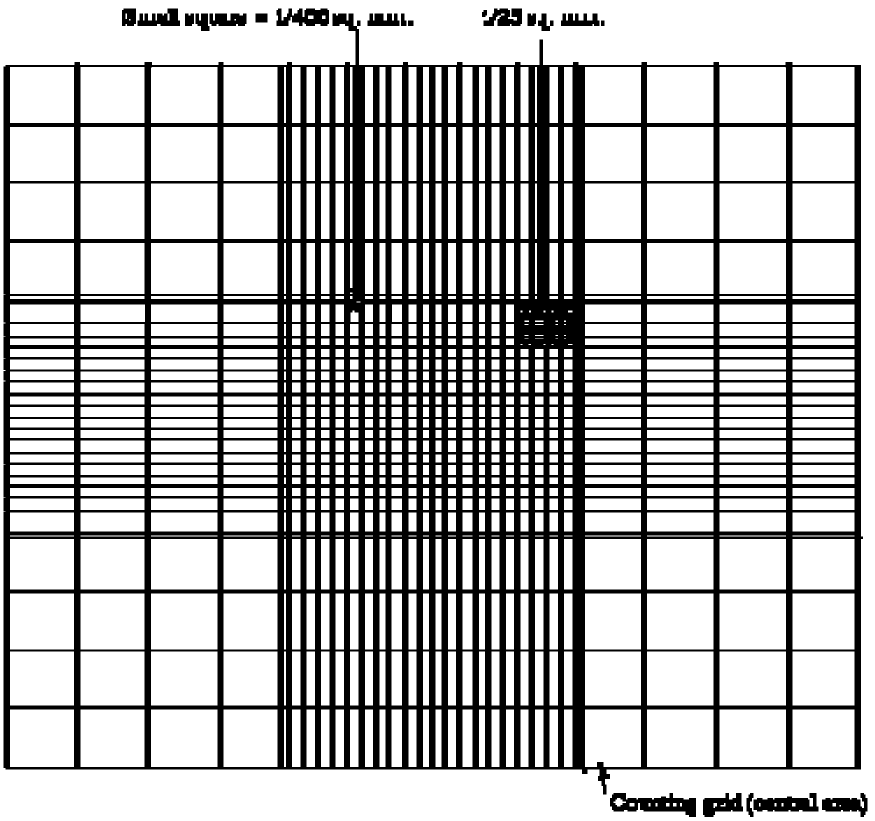

- One entire grid on standard hemocytometers with Neubauer rulings can be seen at 40x (4x objective). The main divisions separate the grid into 9 large squares (like a tic-tac-toe grid). Each square has a surface area of one square mm, and the depth of the chamber is 0.1 mm. The entire counting grid lies under a volume of 0.9 mm-cubed.

- To estimate the total number of viable mononuclear cells/ml (T and B lymphocytes) of the suspension, systematically count the four large corner squares. For cells that overlap a ruling, count a cell as "in" if it overlaps the top or right ruling, and "out" if it overlaps the bottom or left ruling.

- Sum the cell counts of these four large squares together (eg. 25 + 32 + 20 + 23).

- To get the final count in cells/ml, multiply this total count by 10,000. Then divide this result by 4 (the total surface area counted). For example suppose you count 100 cells (total) in the four large corner squares. This is the number of cells/mL (eg. 100 cells x 10,000 ÷ 4 = 250,000 cells/mL).

- Finally, multiply the number above by 2 to get the total number of cells (since you diluted the cells 1:1 with Trypan blue before counting). In this example, 250,000 cells/mL x 2 = 500,000 cells/mL (or 500,000 cells total, since you are working with 1mL of cell suspension). A summary of this equation is below:

$$\#\text{ viable mononuclear cells/ml} = \frac{\text{total cells counted in 4 large squares} \times 10^4 \times 2}{\text{4 large squares counted}}$$

Vaginal Biopsies:

Biopsies will be collected at the enrollment visit and two-week follow-up visit and will be stored under specified conditions pending shipment in batches on dry ice to the central laboratory. For each biopsy sample the following procedure applies:

- Weigh labeled cryogenic vial prior to specimen placement.
- Place biopsy specimen in vial and weigh biopsy and vial.
- Snap freeze either in a liquid nitrogen bath or in an ethanol or acetone bath containing dry ice.
- Store at -80º C.
- The specimens will be stored at the site pending shipment in batches on dry ice to the central laboratory (UNC).

APPENDIX VI: DAIDS TABLE FOR GRADING THE SEVERITY OF ADVERSE EVENTS

Publish Date: December, 2004

Quick Reference

The Division of AIDS Table for Grading the Severity of Adult and Pediatric Adverse Events ("DAIDS AE grading table") is a descriptive terminology which can be utilized for Adverse Event (AE) reporting. A grading (severity) scale is provided for each AE term.

General Instructions

Estimating Severity Grade

If the need arises to grade a clinical AE that is not identified in the DAIDS AE grading table, use the category "Estimating Severity Grade" located at the top of Page 3. For AEs that are not listed in the table but will be collected systematically for a study/trial, protocol teams are highly encouraged to define study-specific severity scales within the protocol or an appendix to the protocol. (Please see "Template Wording for the Expedited Adverse Event Reporting Section of DAIDS-sponsored Protocols".) This is particularly important for laboratory values because the "Estimating Severity Grade" category only applies to clinical symptoms.

Grading Adult and Pediatric AEs

The DAIDS AE grading table includes parameters for grading both Adult and Pediatric AEs. When a single set of parameters is not appropriate for grading specific types of AEs for both Adult and Pediatric populations, separate sets of parameters for Adult and/or Pediatric populations (with specified respective age ranges) are given in the table. If there is no distinction in the table between Adult and Pediatric values for a type of AE, then the single set of parameters listed is to be used for grading the severity of both Adult and Pediatric events of that type.

Determining Severity Grade

If the severity of an AE could fall under either one of two grades (e.g., the severity of an AE could be either Grade 2 or Grade 3), select the higher of the two grades for the AE.

Definitions

Basic Self-care Functions	Adult<br>Activities such as bathing, dressing, toileting, transfer/movement, continence, and feeding.<br>Young Children<br>Activities that are age and culturally appropriate (e.g., feeding self with culturally appropriate eating implement).
LLN	Lower limit of normal
Medical Intervention	Use of pharmacologic or biologic agent(s) for treatment of an AE.
NA	Not Applicable
Operative Intervention	Surgical OR other invasive mechanical procedures.
ULN	Upper limit of normal
Usual Social & Functional Activities	Adult<br>Adaptive tasks and desirable activities, such as going to work, shopping, cooking, use of transportation, pursuing a hobby, etc.<br>Young Children<br>Activities that are age and culturally appropriate (e.g., social interactions, play activities, learning tasks, etc.).

Contents

Clinical — Page

Estimating Severity Grade 3
Systemic...3
Infection...4
Injection Site Reactions 4
Skin – Dermatological 5
Cardiovascular 5
Gastrointestinal 7
Neurologic 9
Respiratory...12
Musculoskeletal..12
Genitourinary..13
Ocular/Visual 14
Endocrine/Metabolic.......................................14

Laboratory — Page

Hematology 16
Chemistries 17
Urinalysis..20

CLINICAL				
**PARAMETER**	**GRADE 1 MILD**	**GRADE 2 MODERATE**	**GRADE 3 SEVERE**	**GRADE 4 POTENTIALLY LIFE-THREATENING**
**ESTIMATING SEVERITY GRADE**				
Clinical adverse event NOT identified elsewhere in this DAIDS AE grading table	Symptoms causing no or minimal interference with usual social & functional activities	Symptoms causing greater than minimal interference with usual social & functional activities	Symptoms causing inability to perform usual social & functional activities	Symptoms causing inability to perform basic self-care functions OR Medical or operative intervention indicated to prevent permanent impairment, persistent disability, or death
**SYSTEMIC**				
Acute systemic allergic reaction	Localized urticaria (wheals) with no medical intervention indicated	Localized urticaria with medical intervention indicated OR Mild angioedema with no medical intervention indicated	Generalized urticaria OR Angioedema with medical intervention indicated OR Symptomatic mild bronchospasm	Acute anaphylaxis OR Life-threatening bronchospasm OR laryngeal edema
Chills	Symptoms causing no or minimal interference with usual social & functional activities	Symptoms causing greater than minimal interference with usual social & functional activities	Symptoms causing inability to perform usual social & functional activities	NA
Fatigue<br>Malaise	Symptoms causing no or minimal interference with usual social & functional activities	Symptoms causing greater than minimal interference with usual social & functional activities	Symptoms causing inability to perform usual social & functional activities	Incapacitating fatigue/ malaise symptoms causing inability to perform basic self-care functions
Fever (nonaxillary)	37.7 – 38.6°C	38.7 – 39.3°C	39.4 – 40.5°C	> 40.5°C
Pain (indicate body site)<br>DO NOT use for pain due to injection (See Injection Site Reactions: Injection site pain)<br>See also Headache, Arthralgia, and Myalgia	Pain causing no or minimal interference with usual social & functional activities	Pain causing greater than minimal interference with usual social & functional activities	Pain causing inability to perform usual social & functional activities	Disabling pain causing inability to perform basic self-care functions OR Hospitalization (other than emergency room visit) indicated

Basic Self-care Functions – Adult: Activities such as bathing, dressing, toileting, transfer/movement, continence, and feeding.

Basic Self-care Functions – Young Children: Activities that are age and culturally appropriate (e.g., feeding self with culturally appropriate eating implement).

Usual Social & Functional Activities – Adult: Adaptive tasks and desirable activities, such as going to work, shopping, cooking, use of transportation, pursuing a hobby, etc.

Usual Social & Functional Activities – Young Children: Activities that are age and culturally appropriate (e.g., social interactions, play activities, learning tasks, etc.).

CLINICAL				
**PARAMETER**	**GRADE 1 MILD**	**GRADE 2 MODERATE**	**GRADE 3 SEVERE**	**GRADE 4 POTENTIALLY LIFE-THREATENING**
Unintentional weight loss	NA	5 – 9% loss in body weight from baseline	10 – 19% loss in body weight from baseline	≥ 20% loss in body weight from baseline OR Aggressive intervention indicated [e.g., tube feeding or total parenteral nutrition (TPN)]
**INFECTION**				
Infection (any other than HIV infection)	Localized, no systemic antimicrobial treatment indicated AND Symptoms causing no or minimal interference with usual social & functional activities	Systemic antimicrobial treatment indicated OR Symptoms causing greater than minimal interference with usual social & functional activities	Systemic antimicrobial treatment indicated AND Symptoms causing inability to perform usual social & functional activities OR Operative intervention (other than simple incision and drainage) indicated	Life-threatening consequences (e.g., septic shock)
**INJECTION SITE REACTIONS**				
Injection site pain (pain without touching) Or Tenderness (pain when area is touched)	Pain/tenderness causing no or minimal limitation of use of limb	Pain/tenderness limiting use of limb OR Pain/tenderness causing greater than minimal interference with usual social & functional activities	Pain/tenderness causing inability to perform usual social & functional activities	Pain/tenderness causing inability to perform basic self-care function OR Hospitalization (other than emergency room visit) indicated for management of pain/tenderness
Injection site reaction (localized)				
**Adult > 15 years**	Erythema OR Induration of 5x5 cm – 9x9 cm (or 25 cm^2 – 81cm^2)	Erythema OR Induration OR Edema > 9 cm any diameter (or > 81 cm^2)	Ulceration OR Secondary infection OR Phlebitis OR Sterile abscess OR Drainage	Necrosis (involving dermis and deeper tissue)

Basic Self-care Functions – Adult: Activities such as bathing, dressing, toileting, transfer/movement, continence, and feeding.

Basic Self-care Functions – Young Children: Activities that are age and culturally appropriate (e.g., feeding self with culturally appropriate eating implement).

Usual Social & Functional Activities – Adult: Adaptive tasks and desirable activities, such as going to work, shopping, cooking, use of transportation, pursuing a hobby, etc.

Usual Social & Functional Activities – Young Children: Activities that are age and culturally appropriate (e.g., social interactions, play activities, learning tasks, etc.).

CLINICAL				
**PARAMETER**	**GRADE 1<br>MILD**	**GRADE 2<br>MODERATE**	**GRADE 3<br>SEVERE**	**GRADE 4<br>POTENTIALLY LIFE-THREATENING**
**Pediatric ≤ 15 years**	Erythema OR Induration OR Edema present but ≤ 2.5 cm diameter	Erythema OR Induration OR Edema > 2.5 cm diameter but < 50% surface area of the extremity segment (e.g., upper arm/thigh)	Erythema OR Induration OR Edema involving ≥ 50% surface area of the extremity segment (e.g., upper arm/thigh) OR Ulceration OR Secondary infection OR Phlebitis OR Sterile abscess OR Drainage	Necrosis (involving dermis and deeper tissue)
Pruritis associated with injection<br>See also Skin: Pruritis (itching - no skin lesions)	Itching localized to injection site AND Relieved spontaneously or with < 48 hours treatment	Itching beyond the injection site but not generalized OR Itching localized to injection site requiring ≥ 48 hours treatment	Generalized itching causing inability to perform usual social & functional activities	NA
**SKIN – DERMATOLOGICAL**				
Alopecia	Thinning detectable by study participant (or by caregiver for young children and disabled adults)	Thinning or patchy hair loss detectable by health care provider	Complete hair loss	NA
Cutaneous reaction – rash	Localized macular rash	Diffuse macular, maculopapular, or morbilliform rash OR Target lesions	Diffuse macular, maculopapular, or morbilliform rash with vesicles or limited number of bullae OR Superficial ulcerations of mucous membrane limited to one site	Extensive or generalized bullous lesions OR Stevens-Johnson syndrome OR Ulceration of mucous membrane involving two or more distinct mucosal sites OR Toxic epidermal necrolysis (TEN)
Hyperpigmentation	Slight or localized	Marked or generalized	NA	NA
Hypopigmentation	Slight or localized	Marked or generalized	NA	NA
Pruritis (itching – no skin lesions)<br>(See also Injection Site Reactions: Pruritis associated with injection)	Itching causing no or minimal interference with usual social & functional activities	Itching causing greater than minimal interference with usual social & functional activities	Itching causing inability to perform usual social & functional activities	NA

Basic Self-care Functions – Adult: Activities such as bathing, dressing, toileting, transfer/movement, continence, and feeding.

Basic Self-care Functions – Young Children: Activities that are age and culturally appropriate (e.g., feeding self with culturally appropriate eating implement).

Usual Social & Functional Activities – Adult: Adaptive tasks and desirable activities, such as going to work, shopping, cooking, use of transportation, pursuing a hobby, etc.

Usual Social & Functional Activities – Young Children: Activities that are age and culturally appropriate (e.g., social interactions, play activities, learning tasks, etc.).

CLINICAL				
**PARAMETER**	**GRADE 1 MILD**	**GRADE 2 MODERATE**	**GRADE 3 SEVERE**	**GRADE 4 POTENTIALLY LIFE-THREATENING**
**CARDIOVASCULAR**				
Cardiac arrhythmia (general)<br>(By ECG or physical exam)	Asymptomatic AND No intervention indicated	Asymptomatic AND Non-urgent medical intervention indicated	Symptomatic, non-life-threatening AND Non-urgent medical intervention indicated	Life-threatening arrhythmia OR Urgent intervention indicated
Cardiac-ischemia/infarction	NA	NA	Symptomatic ischemia (stable angina) OR Testing consistent with ischemia	Unstable angina OR Acute myocardial infarction
Hemorrhage (significant acute blood loss)	NA	Symptomatic AND No transfusion indicated	Symptomatic AND Transfusion of ≤ 2 units packed RBCs (for children ≤ 10 cc/kg) indicated	Life-threatening hypotension OR Transfusion of > 2 units packed RBCs (for children > 10 cc/kg) indicated
Hypertension				
**Adult > 17 years**<br>(with repeat testing at same visit)	> 140 – 159 mmHg systolic<br>OR<br>> 90 – 99 mmHg diastolic	> 160 – 179 mmHg systolic<br>OR<br>> 100 – 109 mmHg diastolic	> 180 mmHg systolic<br>OR<br>> 110 mmHg diastolic	Life-threatening consequences (e.g., malignant hypertension) OR Hospitalization indicated (other than emergency room visit)
**Pediatric ≤ 17 years**<br>(with repeat testing at same visit)	NA	91st – 94th percentile adjusted for age, height, and gender (systolic and/or diastolic)	≥ 95th percentile adjusted for age, height, and gender (systolic and/or diastolic)	Life-threatening consequences (e.g., malignant hypertension) OR Hospitalization indicated (other than emergency room visit)
Hypotension	NA	Symptomatic, corrected with oral fluid replacement	Symptomatic, IV fluids indicated	Shock requiring use of vasopressors or mechanical assistance to maintain blood pressure

Basic Self-care Functions – Adult: Activities such as bathing, dressing, toileting, transfer/movement, continence, and feeding.

Basic Self-care Functions – Young Children: Activities that are age and culturally appropriate (e.g., feeding self with culturally appropriate eating implement).

Usual Social & Functional Activities – Adult: Adaptive tasks and desirable activities, such as going to work, shopping, cooking, use of transportation, pursuing a hobby, etc.

Usual Social & Functional Activities – Young Children: Activities that are age and culturally appropriate (e.g., social interactions, play activities, learning tasks, etc.).

CLINICAL				
**PARAMETER**	**GRADE 1 MILD**	**GRADE 2 MODERATE**	**GRADE 3 SEVERE**	**GRADE 4 POTENTIALLY LIFE-THREATENING**
Pericardial effusion	Asymptomatic, small effusion requiring no intervention	Asymptomatic, moderate or larger effusion requiring no intervention	Effusion with non-life threatening physiologic consequences OR Effusion with non-urgent intervention indicated	Life-threatening consequences (e.g., tamponade) OR Urgent intervention indicated
Prolonged PR interval				
**Adult > 16 years**	PR interval 0.21 – 0.25 sec	PR interval > 0.25 sec	Type II 2nd degree AV block OR Ventricular pause > 3.0 sec	Complete AV block
**Pediatric ≤ 16 years**	1st degree AV block (PR > normal for age and rate)	Type I 2nd degree AV block	Type II 2nd degree AV block	Complete AV block
Prolonged QTc				
**Adult > 16 years**	Asymptomatic, QTc interval 0.45 – 0.47 sec OR Increase interval < 0.03 sec above baseline	Asymptomatic, QTc interval 0.48 – 0.49 sec OR Increase in interval 0.03 – 0.05 sec above baseline	Asymptomatic, QTc interval ≥ 0.50 sec OR Increase in interval ≥ 0.06 sec above baseline	Life-threatening consequences, e.g. Torsade de pointes or other associated serious ventricular dysrhythmia
**Pediatric ≤ 16 years**	Asymptomatic, QTc interval 0.450 – 0.464 sec	Asymptomatic, QTc interval 0.465 – 0.479 sec	Asymptomatic, QTc interval ≥ 0.480 sec	Life-threatening consequences, e.g. Torsade de pointes or other associated serious ventricular dysrhythmia
Thrombosis/embolism	NA	Deep vein thrombosis AND No intervention indicated (e.g., anticoagulation, lysis filter, invasive procedure)	Deep vein thrombosis AND Intervention indicated (e.g., anticoagulation, lysis filter, invasive procedure)	Embolic event (e.g., pulmonary embolism, life-threatening thrombus)
Vasovagal episode (associated with a procedure of any kind)	Present without loss of consciousness	Present with transient loss of consciousness	NA	NA
Ventricular dysfunction (congestive heart failure)	NA	Asymptomatic diagnostic finding AND intervention indicated	New onset with symptoms OR Worsening symptomatic congestive heart failure	Life-threatening congestive heart failure

Basic Self-care Functions – Adult: Activities such as bathing, dressing, toileting, transfer/movement, continence, and feeding.

Basic Self-care Functions – Young Children: Activities that are age and culturally appropriate (e.g., feeding self with culturally appropriate eating implement).

Usual Social & Functional Activities – Adult: Adaptive tasks and desirable activities, such as going to work, shopping, cooking, use of transportation, pursuing a hobby, etc.

Usual Social & Functional Activities – Young Children: Activities that are age and culturally appropriate (e.g., social interactions, play activities, learning tasks, etc.).

CLINICAL				
**PARAMETER**	**GRADE 1 MILD**	**GRADE 2 MODERATE**	**GRADE 3 SEVERE**	**GRADE 4 POTENTIALLY LIFE-THREATENING**
**GASTROINTESTINAL**				
Anorexia	Loss of appetite without decreased oral intake	Loss of appetite associated with decreased oral intake without significant weight loss	Loss of appetite associated with significant weight loss	Life-threatening consequences OR Aggressive intervention indicated [e.g., tube feeding or total parenteral nutrition (TPN)]
Ascites	Asymptomatic	Symptomatic AND Intervention indicated (e.g., diuretics or therapeutic paracentesis)	Symptomatic despite intervention	Life-threatening consequences
Cholecystitis	NA	Symptomatic AND Medical intervention indicated	Radiologic, endoscopic, or operative intervention indicated	Life-threatening consequences (e.g., sepsis or perforation)
Constipation	NA	Persistent constipation requiring regular use of dietary modifications, laxatives, or enemas	Obstipation with manual evacuation indicated	Life-threatening consequences (e.g., obstruction)
Diarrhea				
**Adult and Pediatric ≥ 1 year**	Transient or intermittent episodes of unformed stools OR Increase of ≤ 3 stools over baseline per 24-hour period	Persistent episodes of unformed to watery stools OR Increase of 4 – 6 stools over baseline per 24-hour period	Bloody diarrhea OR Increase of ≥ 7 stools per 24-hour period OR IV fluid replacement indicated	Life-threatening consequences (e.g., hypotensive shock)
**Pediatric < 1 year**	Liquid stools (more unformed than usual) but usual number of stools	Liquid stools with increased number of stools OR Mild dehydration	Liquid stools with moderate dehydration	Liquid stools resulting in severe dehydration with aggressive rehydration indicated OR Hypotensive shock
Dysphagia-Odynophagia	Symptomatic but able to eat usual diet	Symptoms causing altered dietary intake without medical intervention indicated	Symptoms causing severely altered dietary intake with medical intervention indicated	Life-threatening reduction in oral intake

Basic Self-care Functions – Adult: Activities such as bathing, dressing, toileting, transfer/movement, continence, and feeding.

Basic Self-care Functions – Young Children: Activities that are age and culturally appropriate (e.g., feeding self with culturally appropriate eating implement).

Usual Social & Functional Activities – Adult: Adaptive tasks and desirable activities, such as going to work, shopping, cooking, use of transportation, pursuing a hobby, etc.

Usual Social & Functional Activities – Young Children: Activities that are age and culturally appropriate (e.g., social interactions, play activities, learning tasks, etc.).

CLINICAL				
**PARAMETER**	**GRADE 1 MILD**	**GRADE 2 MODERATE**	**GRADE 3 SEVERE**	**GRADE 4 POTENTIALLY LIFE-THREATENING**
Mucositis/stomatitis (clinical exam)<br>Indicate site (e.g., larynx, oral)<br>See Genitourinary for Vulvovaginitis<br>See also Dysphagia-Odynophagia and Proctitis	Erythema of the mucosa	Patchy pseudomembranes or ulcerations	Confluent pseudomembranes or ulcerations OR Mucosal bleeding with minor trauma	Tissue necrosis OR Diffuse spontaneous mucosal bleeding OR Life-threatening consequences (e.g., aspiration, choking)
Nausea	Transient (< 24 hours) or intermittent nausea with no or minimal interference with oral intake	Persistent nausea resulting in decreased oral intake for 24 – 48 hours	Persistent nausea resulting in minimal oral intake for > 48 hours OR Aggressive rehydration indicated (e.g., IV fluids)	Life-threatening consequences (e.g., hypotensive shock)
Pancreatitis	NA	Symptomatic AND Hospitalization not indicated (other than emergency room visit)	Symptomatic AND Hospitalization indicated (other than emergency room visit)	Life-threatening consequences (e.g., circulatory failure, hemorrhage, sepsis)
Proctitis (functional-symptomatic)<br>Also see Mucositis/stomatitis for clinical exam	Rectal discomfort AND No intervention indicated	Symptoms causing greater than minimal interference with usual social & functional activities OR Medical intervention indicated	Symptoms causing inability to perform usual social & functional activities OR Operative intervention indicated	Life-threatening consequences (e.g., perforation)
Vomiting	Transient or intermittent vomiting with no or minimal interference with oral intake	Frequent episodes of vomiting with no or mild dehydration	Persistent vomiting resulting in orthostatic hypotension OR Aggressive rehydration indicated (e.g., IV fluids)	Life-threatening consequences (e.g., hypotensive shock)

Basic Self-care Functions – Adult: Activities such as bathing, dressing, toileting, transfer/movement, continence, and feeding.

Basic Self-care Functions – Young Children: Activities that are age and culturally appropriate (e.g., feeding self with culturally appropriate eating implement).

Usual Social & Functional Activities – Adult: Adaptive tasks and desirable activities, such as going to work, shopping, cooking, use of transportation, pursuing a hobby, etc.

Usual Social & Functional Activities – Young Children: Activities that are age and culturally appropriate (e.g., social interactions, play activities, learning tasks, etc.).

CLINICAL				
**PARAMETER**	**GRADE 1 MILD**	**GRADE 2 MODERATE**	**GRADE 3 SEVERE**	**GRADE 4 POTENTIALLY LIFE-THREATENING**
**NEUROLOGIC**				
Alteration in personality-behavior or in mood (e.g., agitation, anxiety, depression, mania, psychosis)	Alteration causing no or minimal interference with usual social & functional activities	Alteration causing greater than minimal interference with usual social & functional activities	Alteration causing inability to perform usual social & functional activities	Behavior potentially harmful to self or others (e.g., suicidal and homicidal ideation or attempt, acute psychosis) OR Causing inability to perform basic self-care functions
Altered Mental Status<br>For Dementia, see Cognitive and behavioral/attentional disturbance (including dementia and attention deficit disorder)	Changes causing no or minimal interference with usual social & functional activities	Mild lethargy or somnolence causing greater than minimal interference with usual social & functional activities	Confusion, memory impairment, lethargy, or somnolence causing inability to perform usual social & functional activities	Delirium OR obtundation, OR coma
Ataxia	Asymptomatic ataxia detectable on exam OR Minimal ataxia causing no or minimal interference with usual social & functional activities	Symptomatic ataxia causing greater than minimal interference with usual social & functional activities	Symptomatic ataxia causing inability to perform usual social & functional activities	Disabling ataxia causing inability to perform basic self-care functions
Cognitive and behavioral/attentional disturbance (including dementia and attention deficit disorder)	Disability causing no or minimal interference with usual social & functional activities OR Specialized resources not indicated	Disability causing greater than minimal interference with usual social & functional activities OR Specialized resources on part-time basis indicated	Disability causing inability to perform usual social & functional activities OR Specialized resources on a full-time basis indicated	Disability causing inability to perform basic self-care functions OR Institutionalization indicated
CNS ischemia (acute)	NA	NA	Transient ischemic attack	Cerebral vascular accident (CVA, stroke) with neurological deficit

Basic Self-care Functions – Adult: Activities such as bathing, dressing, toileting, transfer/movement, continence, and feeding.

Basic Self-care Functions – Young Children: Activities that are age and culturally appropriate (e.g., feeding self with culturally appropriate eating implement).

Usual Social & Functional Activities – Adult: Adaptive tasks and desirable activities, such as going to work, shopping, cooking, use of transportation, pursuing a hobby, etc.

Usual Social & Functional Activities – Young Children: Activities that are age and culturally appropriate (e.g., social interactions, play activities, learning tasks, etc.).

CLINICAL				
**PARAMETER**	**GRADE 1 MILD**	**GRADE 2 MODERATE**	**GRADE 3 SEVERE**	**GRADE 4 POTENTIALLY LIFE-THREATENING**
Developmental delay – **Pediatric ≤ 16 years**	Mild developmental delay, either motor or cognitive, as determined by comparison with a developmental screening tool appropriate for the setting	Moderate developmental delay, either motor or cognitive, as determined by comparison with a developmental screening tool appropriate for the setting	Severe developmental delay, either motor or cognitive, as determined by comparison with a developmental screening tool appropriate for the setting	Developmental regression, either motor or cognitive, as determined by comparison with a developmental screening tool appropriate for the setting
Headache	Symptoms causing no or minimal interference with usual social & functional activities	Symptoms causing greater than minimal interference with usual social & functional activities	Symptoms causing inability to perform usual social & functional activities	Symptoms causing inability to perform basic self-care functions OR Hospitalization indicated (other than emergency room visit) OR Headache with significant impairment of alertness or other neurologic function
Insomnia	NA	Difficulty sleeping causing greater than minimal interference with usual social & functional activities	Difficulty sleeping causing inability to perform usual social & functional activities	Disabling insomnia causing inability to perform basic self-care functions
Neuromuscular weakness (including myopathy & neuropathy)	Asymptomatic with decreased strength on exam OR Minimal muscle weakness causing no or minimal interference with usual social & functional activities	Muscle weakness causing greater than minimal interference with usual social & functional activities	Muscle weakness causing inability to perform usual social & functional activities	Disabling muscle weakness causing inability to perform basic self-care functions OR Respiratory muscle weakness impairing ventilation
Neurosensory alteration (including paresthesia and painful neuropathy)	Asymptomatic with sensory alteration on exam or minimal paresthesia causing no or minimal interference with usual social & functional activities	Sensory alteration or paresthesia causing greater than minimal interference with usual social & functional activities	Sensory alteration or paresthesia causing inability to perform usual social & functional activities	Disabling sensory alteration or paresthesia causing inability to perform basic self-care functions

Basic Self-care Functions – Adult: Activities such as bathing, dressing, toileting, transfer/movement, continence, and feeding.

Basic Self-care Functions – Young Children: Activities that are age and culturally appropriate (e.g., feeding self with culturally appropriate eating implement).

Usual Social & Functional Activities – Adult: Adaptive tasks and desirable activities, such as going to work, shopping, cooking, use of transportation, pursuing a hobby, etc.

Usual Social & Functional Activities – Young Children: Activities that are age and culturally appropriate (e.g., social interactions, play activities, learning tasks, etc.).

CLINICAL				
**PARAMETER**	**GRADE 1<br>MILD**	**GRADE 2<br>MODERATE**	**GRADE 3<br>SEVERE**	**GRADE 4<br>POTENTIALLY LIFE-THREATENING**
Seizure: (new onset) **– Adult ≥ 18 years**<br>See also Seizure: (known pre-existing seizure disorder)	NA	1 seizure	2 – 4 seizures	Seizures of any kind which are prolonged, repetitive (e.g., status epilepticus), or difficult to control (e.g., refractory epilepsy)
Seizure: (known pre-existing seizure disorder) **– Adult ≥ 18 years**<br>For worsening of existing epilepsy the grades should be based on an increase from previous level of control to any of these levels.	NA	Increased frequency of pre-existing seizures (non-repetitive) without change in seizure character OR Infrequent break-through seizures while on stable medication in a previously controlled seizure disorder	Change in seizure character from baseline either in duration or quality (e.g., severity or focality)	Seizures of any kind which are prolonged, repetitive (e.g., status epilepticus), or difficult to control (e.g., refractory epilepsy)
Seizure **– Pediatric < 18 years**	Seizure, generalized onset with or without secondary generalization, lasting < 5 minutes with < 24 hours post ictal state	Seizure, generalized onset with or without secondary generalization, lasting 5 – 20 minutes with < 24 hours post ictal state	Seizure, generalized onset with or without secondary generalization, lasting > 20 minutes	Seizure, generalized onset with or without secondary generalization, requiring intubation and sedation
Syncope (not associated with a procedure)	NA	Present	NA	NA
Vertigo	Vertigo causing no or minimal interference with usual social & functional activities	Vertigo causing greater than minimal interference with usual social & functional activities	Vertigo causing inability to perform usual social & functional activities	Disabling vertigo causing inability to perform basic self-care functions
**RESPIRATORY**				
Bronchospasm (acute)	FEV1 or peak flow reduced to 70 – 80%	FEV1 or peak flow 50 – 69%	FEV1 or peak flow 25 – 49%	Cyanosis OR FEV1 or peak flow < 25% OR Intubation
Dyspnea or respiratory distress				

Basic Self-care Functions – Adult: Activities such as bathing, dressing, toileting, transfer/movement, continence, and feeding.

Basic Self-care Functions – Young Children: Activities that are age and culturally appropriate (e.g., feeding self with culturally appropriate eating implement).

Usual Social & Functional Activities – Adult: Adaptive tasks and desirable activities, such as going to work, shopping, cooking, use of transportation, pursuing a hobby, etc.

Usual Social & Functional Activities – Young Children: Activities that are age and culturally appropriate (e.g., social interactions, play activities, learning tasks, etc.).

CLINICAL				
**PARAMETER**	**GRADE 1 MILD**	**GRADE 2 MODERATE**	**GRADE 3 SEVERE**	**GRADE 4 POTENTIALLY LIFE-THREATENING**
**Adult ≥ 14 years**	Dyspnea on exertion with no or minimal interference with usual social & functional activities	Dyspnea on exertion causing greater than minimal interference with usual social & functional activities	Dyspnea at rest causing inability to perform usual social & functional activities	Respiratory failure with ventilatory support indicated
**Pediatric < 14 years**	Wheezing OR minimal increase in respiratory rate for age	Nasal flaring OR Intercostal retractions OR Pulse oximetry 90 – 95%	Dyspnea at rest causing inability to perform usual social & functional activities OR Pulse oximetry < 90%	Respiratory failure with ventilatory support indicated
**MUSCULOSKELETAL**				
Arthralgia<br>See also Arthritis	Joint pain causing no or minimal interference with usual social & functional activities	Joint pain causing greater than minimal interference with usual social & functional activities	Joint pain causing inability to perform usual social & functional activities	Disabling joint pain causing inability to perform basic self-care functions
Arthritis<br>See also Arthralgia	Stiffness or joint swelling causing no or minimal interference with usual social & functional activities	Stiffness or joint swelling causing greater than minimal interference with usual social & functional activities	Stiffness or joint swelling causing inability to perform usual social & functional activities	Disabling joint stiffness or swelling causing inability to perform basic self-care functions
Bone Mineral Loss				
**Adult ≥ 21 years**	BMD t-score -2.5 to -1.0	BMD t-score < -2.5	Pathological fracture (including loss of vertebral height)	Pathologic fracture causing life-threatening consequences
**Pediatric < 21 years**	BMD z-score -2.5 to -1.0	BMD z-score < -2.5	Pathological fracture (including loss of vertebral height)	Pathologic fracture causing life-threatening consequences
Myalgia (non-injection site)	Muscle pain causing no or minimal interference with usual social & functional activities	Muscle pain causing greater than minimal interference with usual social & functional activities	Muscle pain causing inability to perform usual social & functional activities	Disabling muscle pain causing inability to perform basic self-care functions
Osteonecrosis	NA	Asymptomatic with radiographic findings AND No operative intervention indicated	Symptomatic bone pain with radiographic findings OR Operative intervention indicated	Disabling bone pain with radiographic findings causing inability to perform basic self-care functions

Basic Self-care Functions – Adult: Activities such as bathing, dressing, toileting, transfer/movement, continence, and feeding.

Basic Self-care Functions – Young Children: Activities that are age and culturally appropriate (e.g., feeding self with culturally appropriate eating implement).

Usual Social & Functional Activities – Adult: Adaptive tasks and desirable activities, such as going to work, shopping, cooking, use of transportation, pursuing a hobby, etc.

Usual Social & Functional Activities – Young Children: Activities that are age and culturally appropriate (e.g., social interactions, play activities, learning tasks, etc.).

CLINICAL				
**PARAMETER**	**GRADE 1 MILD**	**GRADE 2 MODERATE**	**GRADE 3 SEVERE**	**GRADE 4 POTENTIALLY LIFE-THREATENING**
**GENITOURINARY**				
Cervicitis (symptoms) (For use in studies evaluating topical study agents) For other cervicitis see Infection: Infection (any other than HIV infection)	Symptoms causing no or minimal interference with usual social & functional activities	Symptoms causing greater than minimal interference with usual social & functional activities	Symptoms causing inability to perform usual social & functional activities	Symptoms causing inability to perform basic self-care functions
Cervicitis (clinical exam) (For use in studies evaluating topical study agents) For other cervicitis see Infection: Infection (any other than HIV infection)	Minimal cervical abnormalities on examination (erythema, mucopurulent discharge, or friability) OR Epithelial disruption < 25% of total surface	Moderate cervical abnormalities on examination (erythema, mucopurulent discharge, or friability) OR Epithelial disruption of 25 – 49% total surface	Severe cervical abnormalities on examination (erythema, mucopurulent discharge, or friability) OR Epithelial disruption 50 – 75% total surface	Epithelial disruption > 75% total surface
Inter-menstrual bleeding (IMB)	Spotting observed by participant OR Minimal blood observed during clinical or colposcopic examination	Inter-menstrual bleeding not greater in duration or amount than usual menstrual cycle	Inter-menstrual bleeding greater in duration or amount than usual menstrual cycle	Hemorrhage with life-threatening hypotension OR Operative intervention indicated
Urinary tract obstruction (e.g., stone)	NA	Signs or symptoms of urinary tract obstruction without hydronephrosis or renal dysfunction	Signs or symptoms of urinary tract obstruction with hydronephrosis or renal dysfunction	Obstruction causing life-threatening consequences

Basic Self-care Functions – Adult: Activities such as bathing, dressing, toileting, transfer/movement, continence, and feeding.

Basic Self-care Functions – Young Children: Activities that are age and culturally appropriate (e.g., feeding self with culturally appropriate eating implement).

Usual Social & Functional Activities – Adult: Adaptive tasks and desirable activities, such as going to work, shopping, cooking, use of transportation, pursuing a hobby, etc.

Usual Social & Functional Activities – Young Children: Activities that are age and culturally appropriate (e.g., social interactions, play activities, learning tasks, etc.).

CLINICAL				
**PARAMETER**	**GRADE 1 MILD**	**GRADE 2 MODERATE**	**GRADE 3 SEVERE**	**GRADE 4 POTENTIALLY LIFE-THREATENING**
Vulvovaginitis (symptoms)<br>(Use in studies evaluating topical study agents)<br>For other vulvovaginitis see Infection: Infection (any other than HIV infection)	Symptoms causing no or minimal interference with usual social & functional activities	Symptoms causing greater than minimal interference with usual social & functional activities	Symptoms causing inability to perform usual social & functional activities	Symptoms causing inability to perform basic self-care functions
Vulvovaginitis (clinical exam)<br>(Use in studies evaluating topical study agents)<br>For other vulvovaginitis see Infection: Infection (any other than HIV infection)	Minimal vaginal abnormalities on examination OR Epithelial disruption < 25% of total surface	Moderate vaginal abnormalities on examination OR Epithelial disruption of 25 - 49% total surface	Severe vaginal abnormalities on examination OR Epithelial disruption 50 - 75% total surface	Vaginal perforation OR Epithelial disruption > 75% total surface
**OCULAR/VISUAL**				
Uveitis	Asymptomatic but detectable on exam	Symptomatic anterior uveitis OR Medical intervention indicated	Posterior or pan-uveitis OR Operative intervention indicated	Disabling visual loss in affected eye(s)
Visual changes (from baseline)	Visual changes causing no or minimal interference with usual social & functional activities	Visual changes causing greater than minimal interference with usual social & functional activities	Visual changes causing inability to perform usual social & functional activities	Disabling visual loss in affected eye(s)
**ENDOCRINE/METABOLIC**				
Abnormal fat accumulation (e.g., back of neck, breasts, abdomen)	Detectable by study participant (or by caregiver for young children and disabled adults)	Detectable on physical exam by health care provider	Disfiguring OR Obvious changes on casual visual inspection	NA

Basic Self-care Functions – Adult: Activities such as bathing, dressing, toileting, transfer/movement, continence, and feeding.

Basic Self-care Functions – Young Children: Activities that are age and culturally appropriate (e.g., feeding self with culturally appropriate eating implement).

Usual Social & Functional Activities – Adult: Adaptive tasks and desirable activities, such as going to work, shopping, cooking, use of transportation, pursuing a hobby, etc.

Usual Social & Functional Activities – Young Children: Activities that are age and culturally appropriate (e.g., social interactions, play activities, learning tasks, etc.).

CLINICAL				
**PARAMETER**	**GRADE 1 MILD**	**GRADE 2 MODERATE**	**GRADE 3 SEVERE**	**GRADE 4 POTENTIALLY LIFE-THREATENING**
Diabetes mellitus	NA	New onset without need to initiate medication OR Modification of current medications to regain glucose control	New onset with initiation of medication indicated OR Diabetes uncontrolled despite treatment modification	Life-threatening consequences (e.g., ketoacidosis, hyperosmolar non-ketotic coma)
Gynecomastia	Detectable by study participant or caregiver (for young children and disabled adults)	Detectable on physical exam by health care provider	Disfiguring OR Obvious on casual visual inspection	NA
Hyperthyroidism	Asymptomatic	Symptomatic causing greater than minimal interference with usual social & functional activities OR Thyroid suppression therapy indicated	Symptoms causing inability to perform usual social & functional activities OR Uncontrolled despite treatment modification	Life-threatening consequences (e.g., thyroid storm)
Hypothyroidism	Asymptomatic	Symptomatic causing greater than minimal interference with usual social & functional activities OR Thyroid replacement therapy indicated	Symptoms causing inability to perform usual social & functional activities OR Uncontrolled despite treatment modification	Life-threatening consequences (e.g., myxedema coma)
Lipoatrophy (e.g., fat loss from the face, extremities, buttocks)	Detectable by study participant (or by caregiver for young children and disabled adults)	Detectable on physical exam by health care provider	Disfiguring OR Obvious on casual visual inspection	NA

Basic Self-care Functions – Adult: Activities such as bathing, dressing, toileting, transfer/movement, continence, and feeding.

Basic Self-care Functions – Young Children: Activities that are age and culturally appropriate (e.g., feeding self with culturally appropriate eating implement).

Usual Social & Functional Activities – Adult: Adaptive tasks and desirable activities, such as going to work, shopping, cooking, use of transportation, pursuing a hobby, etc.

Usual Social & Functional Activities – Young Children: Activities that are age and culturally appropriate (e.g., social interactions, play activities, learning tasks, etc.).

LABORATORY				
**PARAMETER**	**GRADE 1 MILD**	**GRADE 2 MODERATE**	**GRADE 3 SEVERE**	**GRADE 4 POTENTIALLY LIFE-THREATENING**
**HEMATOLOGY**	***Standard International Units are listed in italics***			
Absolute CD4+ count **– Adult and Pediatric > 13 years** (HIV NEGATIVE ONLY)	300 – 400/mm^3 *300 – 400/µL*	200 – 299/mm^3 *200 – 299/µL*	100 – 199/mm^3 *100 – 199/µL*	< 100/mm^3 *< 100/µL*
Absolute lymphocyte count **– Adult and Pediatric > 13 years** (HIV NEGATIVE ONLY)	600 – 650/mm^3 *0.600 x 10^9 – 0.650 x 10^9/L*	500 – 599/mm^3 *0.500 x 10^9 – 0.599 x 10^9/L*	350 – 499/mm^3 *0.350 x 10^9 – 0.499 x 10^9/L*	< 350/mm^3 *< 0.350 x 10^9/L*
Absolute neutrophil count (ANC)				
**Adult and Pediatric, > 7 days**	1,000 – 1,300/mm^3 *1.000 x 10^9 – 1.300 x 10^9/L*	750 – 999/mm^3 *0.750 x 10^9 – 0.999 x 10^9/L*	500 – 749/mm^3 *0.500 x 10^9 – 0.749 x 10^9/L*	< 500/mm^3 *< 0.500 x 10^9/L*
**Infant*†, 2 – ≤ 7 days**	1,250 – 1,500/mm^3 *1.250 x 10^9 – 1.500 x 10^9/L*	1,000 – 1,249/mm^3 *1.000 x 10^9 – 1.249 x 10^9/L*	750 – 999/mm^3 *0.750 x 10^9 – 0.999 x 10^9/L*	< 750/mm^3 *< 0.750 x 10^9/L*
**Infant*†, 1 day**	4,000 – 5,000/mm^3 *4.000 x 10^9 – 5.000 x 10^9/L*	3,000 – 3,999/mm^3 *3.000 x 10^9 – 3.999 x10^9/L*	1,500 – 2,999/mm^3 *1.500 x 10^9 – 2.999 x 10^9/L*	< 1,500/mm^3 *< 1.500 x 10^9/L*
Fibrinogen, decreased	100 – 200 mg/dL *1.00 – 2.00 g/L* OR 0.75 – 0.99 x LLN	75 – 99 mg/dL *0.75 – 0.99 g/L* OR 0.50 – 0.74 x LLN	50 – 74 mg/dL *0.50 – 0.74 g/L* OR 0.25 – 0.49 x LLN	< 50 mg/dL *< 0.50 g/L* OR < 0.25 x LLN OR Associated with gross bleeding
Hemoglobin (Hgb)				
**Adult and Pediatric ≥ 57 days** (HIV POSITIVE ONLY)	8.5 – 10.0 g/dL *1.32 – 1.55 mmol/L*	7.5 – 8.4 g/dL *1.16 – 1.31 mmol/L*	6.50 – 7.4 g/dL *1.01 – 1.15 mmol/L*	< 6.5 g/dL *< 1.01 mmol/L*
**Adult and Pediatric ≥ 57 days** (HIV NEGATIVE ONLY)	10.0 – 10.9 g/dL *1.55 – 1.69 mmol/L* OR Any decrease 2.5 – 3.4 g/dL *0.39 – 0.53 mmol/L*	9.0 – 9.9 g/dL *1.40 – 1.54 mmol/L* OR Any decrease 3.5 – 4.4 g/dL *0.54 – 0.68 mmol/L*	7.0 – 8.9 g/dL *1.09 – 1.39 mmol/L* OR Any decrease ≥ 4.5 g/dL *≥ 0.69 mmol/L*	< 7.0 g/dL *< 1.09 mmol/L*
**Infant*†, 36 – 56 days** (HIV POSITIVE OR NEGATIVE)	8.5 – 9.4 g/dL *1.32 – 1.46 mmol/L*	7.0 – 8.4 g/dL *1.09 – 1.31 mmol/L*	6.0 – 6.9 g/dL *0.93 – 1.08 mmol/L*	< 6.00 g/dL *< 0.93 mmol/L*

[*] Values are for term infants.

[†] Use age and sex appropriate values (e.g., bilirubin), including preterm infants.

LABORATORY				
**PARAMETER**	**GRADE 1 MILD**	**GRADE 2 MODERATE**	**GRADE 3 SEVERE**	**GRADE 4 POTENTIALLY LIFE-THREATENING**
**Infant*†, 22 – 35 days** (HIV POSITIVE OR NEGATIVE)	9.5 – 10.5 g/dL *1.47 – 1.63 mmol/L*	8.0 – 9.4 g/dL *1.24 – 1.46 mmol/L*	7.0 – 7.9 g/dL *1.09 – 1.23 mmol/L*	< 7.00 g/dL *< 1.09 mmol/L*
**Infant*†, 1 – 21 days** (HIV POSITIVE OR NEGATIVE)	12.0 – 13.0 g/dL *1.86 – 2.02 mmol/L*	10.0 – 11.9 g/dL *1.55 – 1.85 mmol/L*	9.0 – 9.9 g/dL *1.40 – 1.54 mmol/L*	< 9.0 g/dL *< 1.40 mmol/L*
International Normalized Ratio of prothrombin time (INR)	1.1 – 1.5 x ULN	1.6 – 2.0 x ULN	2.1 – 3.0 x ULN	> 3.0 x ULN
Methemoglobin	5.0 – 10.0%	10.1 – 15.0%	15.1 – 20.0%	> 20.0%
Prothrombin Time (PT)	1.1 – 1.25 x ULN	1.26 – 1.50 x ULN	1.51 – 3.00 x ULN	> 3.00 x ULN
Partial Thromboplastin Time (PTT)	1.1 – 1.66 x ULN	1.67 – 2.33 x ULN	2.34 – 3.00 x ULN	> 3.00 x ULN
Platelets, decreased	100,000 – 124,999/mm^3 *100.000 x 10^9 – 124.999 x 10^9/L*	50,000 – 99,999/mm^3 *50.000 x 10^9 – 99.999 x 10^9/L*	25,000 – 49,999/mm^3 *25.000 x 10^9 – 49.999 x 10^9/L*	< 25,000/mm^3 *< 25.000 x 10^9/L*
WBC, decreased	2,000 – 2,500/mm^3 *2.000 x 10^9 – 2.500 x 10^9/L*	1,500 – 1,999/mm^3 *1.500 x 10^9 – 1.999 x 10^9/L*	1,000 – 1,499/mm^3 *1.000 x 10^9 – 1.499 x 10^9/L*	< 1,000/mm^3 *< 1.000 x 10^9/L*
**CHEMISTRIES**	***Standard International Units are listed in italics***			
Acidosis	NA	pH < normal, but ≥ 7.3	pH < 7.3 without life-threatening consequences	pH < 7.3 with life-threatening consequences
Albumin, serum, low	3.0 g/dL – < LLN *30 g/L – < LLN*	2.0 – 2.9 g/dL *20 – 29 g/L*	< 2.0 g/dL *< 20 g/L*	NA
Alkaline Phosphatase	1.25 – 2.5 x ULN†	2.6 – 5.0 x ULN†	5.1 – 10.0 x ULN†	> 10.0 x ULN†
Alkalosis	NA	pH > normal, but ≤ 7.5	pH > 7.5 without life-threatening consequences	pH > 7.5 with life-threatening consequences
ALT (SGPT)	1.25 – 2.5 x ULN	2.6 – 5.0 x ULN	5.1 – 10.0 x ULN	> 10.0 x ULN
AST (SGOT)	1.25 – 2.5 x ULN	2.6 – 5.0 x ULN	5.1 – 10.0 x ULN	> 10.0 x ULN
Bicarbonate, serum, low	16.0 mEq/L – < LLN *16.0 mmol/L – < LLN*	11.0 – 15.9 mEq/L *11.0 – 15.9 mmol/L*	8.0 – 10.9 mEq/L *8.0 – 10.9 mmol/L*	< 8.0 mEq/L *< 8.0 mmol/L*
Bilirubin (Total)				
**Adult and Pediatric > 14 days**	1.1 – 1.5 x ULN	1.6 – 2.5 x ULN	2.6 – 5.0 x ULN	> 5.0 x ULN

* Values are for term infants.

† Use age and sex appropriate values (e.g., bilirubin), including preterm infants.

LABORATORY				
**PARAMETER**	**GRADE 1 MILD**	**GRADE 2 MODERATE**	**GRADE 3 SEVERE**	**GRADE 4 POTENTIALLY LIFE-THREATENING**
**Infant*†, ≤ 14 days** (non-hemolytic)	NA	20.0 – 25.0 mg/dL<br>*342 – 428 µmol/L*	25.1 – 30.0 mg/dL<br>*429 – 513 µmol/L*	> 30.0 mg/dL<br>*> 513.0 µmol/L*
**Infant*†, ≤ 14 days** (hemolytic)	NA	NA	20.0 – 25.0 mg/dL<br>*342 – 428 µmol/L*	> 25.0 mg/dL<br>*> 428 µmol/L*
Calcium, serum, high (corrected for albumin)				
**Adult and Pediatric ≥ 7 days**	10.6 – 11.5 mg/dL<br>*2.65 – 2.88 mmol/L*	11.6 – 12.5 mg/dL<br>*2.89 – 3.13 mmol/L*	12.6 – 13.5 mg/dL<br>*3.14 – 3.38 mmol/L*	> 13.5 mg/dL<br>*> 3.38 mmol/L*
**Infant*†, < 7 days**	11.5 – 12.4 mg/dL<br>*2.88 – 3.10 mmol/L*	12.5 – 12.9 mg/dL<br>*3.11 – 3.23 mmol/L*	13.0 – 13.5 mg/dL<br>*3.245 – 3.38 mmol/L*	> 13.5 mg/dL<br>*> 3.38 mmol/L*
Calcium, serum, low (corrected for albumin)				
**Adult and Pediatric ≥ 7 days**	7.8 – 8.4 mg/dL<br>*1.95 – 2.10 mmol/L*	7.0 – 7.7 mg/dL<br>*1.75 – 1.94 mmol/L*	6.1 – 6.9 mg/dL<br>*1.53 – 1.74 mmol/L*	< 6.1 mg/dL<br>*< 1.53 mmol/L*
**Infant*†, < 7 days**	6.5 – 7.5 mg/dL<br>*1.63 – 1.88 mmol/L*	6.0 – 6.4 mg/dL<br>*1.50 – 1.62 mmol/L*	5.50 – 5.90 mg/dL<br>*1.38 – 1.51 mmol/L*	< 5.50 mg/dL<br>*< 1.38 mmol/L*
Cardiac troponin I (cTnI)	NA	NA	NA	Levels consistent with myocardial infarction or unstable angina as defined by the manufacturer
Cardiac troponin T (cTnT)	NA	NA	NA	≥ 0.20 ng/mL<br>OR<br>Levels consistent with myocardial infarction or unstable angina as defined by the manufacturer
Cholesterol (fasting)				
**Adult ≥ 18 years**	200 – 239 mg/dL<br>*5.18 – 6.19 mmol/L*	240 – 300 mg/dL<br>*6.20 – 7.77 mmol/L*	> 300 mg/dL<br>*> 7.77 mmol/L*	NA
**Pediatric < 18 years**	170 – 199 mg/dL<br>*4.40 – 5.15 mmol/L*	200 – 300 mg/dL<br>*5.16 – 7.77 mmol/L*	> 300 mg/dL<br>*> 7.77 mmol/L*	NA
Creatine Kinase	3.0 – 5.9 x ULN†	6.0 – 9.9 x ULN†	10.0 – 19.9 x ULN†	≥ 20.0 x ULN†
Creatinine	1.1 – 1.3 x ULN†	1.4 – 1.8 x ULN†	1.9 – 3.4 x ULN†	≥ 3.5 x ULN†
Glucose, serum, high				
Nonfasting	116 – 160 mg/dL<br>*6.44 – 8.88 mmol/L*	161 – 250 mg/dL<br>*8.89 – 13.88 mmol/L*	251 – 500 mg/dL<br>*13.89 – 27.75 mmol/L*	> 500 mg/dL<br>*> 27.75 mmol/L*
Fasting	110 – 125 mg/dL<br>*6.11 – 6.94 mmol/L*	126 – 250 mg/dL<br>*6.95 – 13.88 mmol/L*	251 – 500 mg/dL<br>*13.89 – 27.75 mmol/L*	> 500 mg/dL<br>*> 27.75 mmol/L*

* Values are for term infants.

† Use age and sex appropriate values (e.g., bilirubin), including preterm infants.

LABORATORY				
**PARAMETER**	**GRADE 1 MILD**	**GRADE 2 MODERATE**	**GRADE 3 SEVERE**	**GRADE 4 POTENTIALLY LIFE-THREATENING**
Glucose, serum, low				
**Adult and Pediatric ≥ 1 month**	55 – 64 mg/dL<br>*3.05 – 3.55 mmol/L*	40 – 54 mg/dL<br>*2.22 – 3.06 mmol/L*	30 – 39 mg/dL<br>*1.67 – 2.23 mmol/L*	< 30 mg/dL<br>*< 1.67 mmol/L*
**Infant*†, < 1 month**	50 – 54 mg/dL<br>*2.78 – 3.00 mmol/L*	40 – 49 mg/dL<br>*2.22 – 2.77 mmol/L*	30 – 39 mg/dL<br>*1.67 – 2.21 mmol/L*	< 30 mg/dL<br>*< 1.67 mmol/L*
Lactate	< 2.0 x ULN without acidosis	≥ 2.0 x ULN without acidosis	Increased lactate with pH < 7.3 without life-threatening consequences	Increased lactate with pH < 7.3 with life-threatening consequences
LDL cholesterol (fasting)				
**Adult ≥ 18 years**	130 – 159 mg/dL<br>*3.37 – 4.12 mmol/L*	160 – 190 mg/dL<br>*4.13 – 4.90 mmol/L*	≥ 190 mg/dL<br>*≥ 4.91 mmol/L*	NA
**Pediatric > 2 - < 18 years**	110 – 129 mg/dL<br>*2.85 – 3.34 mmol/L*	130 – 189 mg/dL<br>*3.35 – 4.90 mmol/L*	≥ 190 mg/dL<br>*≥ 4.91 mmol/L*	NA
Lipase	1.1 – 1.5 x ULN	1.6 – 3.0 x ULN	3.1 – 5.0 x ULN	> 5.0 x ULN
Magnesium, serum, low	1.2 – 1.4 mEq/L<br>*0.60 – 0.70 mmol/L*	0.9 – 1.1 mEq/L<br>*0.45 – 0.59 mmol/L*	0.6 – 0.8 mEq/L<br>*0.30 – 0.44 mmol/L*	< 0.60 mEq/L<br>*< 0.30 mmol/L*
Pancreatic amylase	1.1 – 1.5 x ULN	1.6 – 2.0 x ULN	2.1 – 5.0 x ULN	> 5.0 x ULN
Phosphate, serum, low				
**Adult and Pediatric > 14 years**	2.5 mg/dL – < LLN<br>*0.81 mmol/L – < LLN*	2.0 – 2.4 mg/dL<br>*0.65 – 0.80 mmol/L*	1.0 – 1.9 mg/dL<br>*0.32 – 0.64 mmol/L*	< 1.00 mg/dL<br>*< 0.32 mmol/L*
**Pediatric 1 year – 14 years**	3.0 – 3.5 mg/dL<br>*0.97 – 1.13 mmol/L*	2.5 – 2.9 mg/dL<br>*0.81 – 0.96 mmol/L*	1.5 – 2.4 mg/dL<br>*0.48 – 0.80 mmol/L*	< 1.50 mg/dL<br>*< 0.48 mmol/L*
**Pediatric < 1 year**	3.5 – 4.5 mg/dL<br>*1.13 – 1.45 mmol/L*	2.5 – 3.4 mg/dL<br>*0.81 – 1.12 mmol/L*	1.5 – 2.4 mg/dL<br>*0.48 – 0.80 mmol/L*	< 1.50 mg/dL<br>*< 0.48 mmol/L*
Potassium, serum, high	5.6 – 6.0 mEq/L<br>*5.6 – 6.0 mmol/L*	6.1 – 6.5 mEq/L<br>*6.1 – 6.5 mmol/L*	6.6 – 7.0 mEq/L<br>*6.6 – 7.0 mmol/L*	> 7.0 mEq/L<br>*> 7.0 mmol/L*
Potassium, serum, low	3.0 – 3.4 mEq/L<br>*3.0 – 3.4 mmol/L*	2.5 – 2.9 mEq/L<br>*2.5 – 2.9 mmol/L*	2.0 – 2.4 mEq/L<br>*2.0 – 2.4 mmol/L*	< 2.0 mEq/L<br>*< 2.0 mmol/L*
Sodium, serum, high	146 – 150 mEq/L<br>*146 – 150 mmol/L*	151 – 154 mEq/L<br>*151 – 154 mmol/L*	155 – 159 mEq/L<br>*155 – 159 mmol/L*	≥ 160 mEq/L<br>*≥ 160 mmol/L*
Sodium, serum, low	130 – 135 mEq/L<br>*130 – 135 mmol/L*	125 – 129 mEq/L<br>*125 – 129 mmol/L*	121 – 124 mEq/L<br>*121 – 124 mmol/L*	≤ 120 mEq/L<br>*≤ 120 mmol/L*
Triglycerides (fasting)	NA	500 – 750 mg/dL<br>*5.65 – 8.48 mmol/L*	751 – 1,200 mg/dL<br>*8.49 – 13.56 mmol/L*	> 1,200 mg/dL<br>*> 13.56 mmol/L*

* Values are for term infants.

† Use age and sex appropriate values (e.g., bilirubin), including preterm infants.

LABORATORY				
**PARAMETER**	**GRADE 1 MILD**	**GRADE 2 MODERATE**	**GRADE 3 SEVERE**	**GRADE 4 POTENTIALLY LIFE-THREATENING**
Uric acid	7.5 – 10.0 mg/dL<br>*0.45 – 0.59 mmol/L*	10.1 – 12.0 mg/dL<br>*0.60 – 0.71 mmol/L*	12.1 – 15.0 mg/dL<br>*0.72 – 0.89 mmol/L*	> 15.0 mg/dL<br>*> 0.89 mmol/L*
**URINALYSIS**	***Standard International Units are listed in italics***			
Hematuria (microscopic)	6 – 10 RBC/HPF	> 10 RBC/HPF	Gross, with or without clots OR with RBC casts	Transfusion indicated
Proteinuria, random collection	1 +	2 – 3 +	4 +	NA
Proteinuria, 24 hour collection				
**Adult and Pediatric ≥ 10 years**	200 – 999 mg/24 h<br>*0.200 – 0.999 g/d*	1,000 – 1,999 mg/24 h<br>*1.000 – 1.999 g/d*	2,000 – 3,500 mg/24 h<br>*2.000 – 3.500 g/d*	> 3,500 mg/24 h<br>*> 3.500 g/d*
**Pediatric > 3 mo - < 10 years**	201 – 499 $mg/m^2/24$ h<br>*0.201 – 0.499 g/d*	500 – 799 $mg/m^2/24$ h<br>*0.500 – 0.799 g/d*	800 – 1,000 $mg/m^2/24$ h<br>*0.800 – 1.000 g/d*	> 1,000 mg/ $m^2/24$ h<br>*> 1.000 g/d*

* Values are for term infants.

† Use age and sex appropriate values (e.g., bilirubin), including preterm infants.

APPENDIX VII: MODEL CONSENT FORM

The model consent(s) should be modified as necessary to meet site IRB and state law regulations as well as HIPAA regulations.

TITLE: Single Dose and 14-Day Once or Twice-daily Pharmacokinetic Study of the Vaginal Microbicide Agent 1% Tenofovir Gel

INVESTIGATOR:
To be completed

ADDRESS OF STUDY SITE:
To be completed

TELEPHONE NUMBERS:
Daytime:
After Hours:

SPONSOR: CONRAD is receiving funding for this study from International Partnership for Microbicides.

Introduction

You are being asked to participate in a research study because you are a healthy woman between the ages of 18 and 45, are in general good health, have regular menstrual cycles and are at least 2 months since your last pregnancy. Before you decide if you want to join this study, we want you to know about this study. This consent form gives you information about this study and the study staff will talk with you about this information and answer any questions you may have. If you agree to take part in this study, you will be asked to sign this consent form and will be given a signed copy to keep.

Description

This study is researching a new investigational vaginal gel containing tenofovir. An investigational gel is one that has not been approved by the U.S. Food and Drug Administration (FDA). Although tenofovir pills have been approved by the FDA to treat HIV (human immunodeficiency virus, the virus that causes AIDS), the gel is investigational because it is a different formulation and we do not know all of the effects it may have. These gels are being studied because they may destroy HIV.

The main purpose of this study is to find out how much, if any, tenofovir gel, when used once and then for 2 weeks will be absorbed into your vaginal tissues. The other purpose of the study is to find out how much, if any, tenofovir gel is absorbed from the vagina into the blood. Your selection as a participant will be based upon your medical and physical qualifications.

If you are selected, you will be one of about 49 women in this study. There will be two parts to this study. For the first part, you will receive one dose of tenofovir gel, and will provide blood samples

over the next 24 hours. You will also be randomly assigned (like the flip of a coin) to one of seven different time groups for the collection of samples of vaginal fluids, cells and tissues after using a single dose of tenofovir gel. Then there will be a rest period of about one month. For the second part of the study, you will be randomly assigned (like the flip of a coin) to use the gel either once a day or twice a day for two weeks. After one week of gel use, you will provide vaginal and cervical samples. After two weeks of gel use, you will provide blood samples over the next 24 hours and there will be 3 different time groups that you will be randomly assigned (like the flip of a coin) to for the collection of samples of vaginal fluids, cells and tissues. You cannot choose your groups, and the study staff cannot choose your groups for you. You have an equal chance of being placed in each one of the groups. If enrolled, you would have at least 6 study visits and your participation in this study would last about 4 months.

Up to 10 participants at one of the sites, who have completed the first two phases of the study, will be asked to participate in a third phase to have blood samples, vaginal fluids, cells and tissues collected 12 hours after a single dose of tenofovir gel.

Exclusions

You will not be allowed to participate in the study if you:

- Have irregular menstrual cycles
- Are pregnant, are less than 2 months from your last pregnancy or are breastfeeding
- Are positive for HIV infection or Hepatitis B
- Have gonorrhea, *Chlamydia* or *Trichomonas* infection
- Have a history of abnormal Pap smear that has not been evaluated and treated, if indicated, according to standard guidelines
- Have used tenofovir or other anti-HIV medication
- Are currently or have recently used drugs or alcohol
- Are using or plan to use on a daily basis medications that may affect your kidneys or liver (like acyclovir, ibuprofen or tylenol)
- Have used hormonal contraceptives in the month prior to enrollment or Depo-Provera in the four (4) months prior to enrollment.
- Have any abnormal findings on laboratory tests or during physical examination or a medical condition which, in the opinion of the study investigator, would make the study unsafe for you or would make it hard for the researchers to interpret the results of the study
- Have participated in any other investigational trial within 30 days prior to screening.

While in this study, you must be protected from pregnancy. Acceptable methods of birth control while in the study include: non-hormonal intrauterine device (IUD), female sterilization (tubes tied), vasectomized partner, effective barrier method or abstinence (no intercourse). You will also be required not to have intercourse or any vaginal activity during the study phases. However, you will be allowed to continue your normal sexual activities between the study phases.

Study Procedures

Visit 1 (Screening)

Procedures to determine if you are eligible to take part in a research study are called “screening procedures”. For this research study, the screening procedures include the following:

1. The study procedures and potential risks will be explained to you. You will read the consent form. After all of your questions have been answered, you will decide if you want to participate in the study. If you decide to participate, you will sign the consent form and you will receive a copy of the signed consent form.

2. Once you have signed the consent form, you will be asked questions to collect medical information to make certain you are eligible for the study. You will also be asked questions about your background (i.e., age, education, etc.) as well as questions about your sexual history and use of birth control methods.

3. Your height and weight will be measured and recorded.

4. A urine sample will be collected to test for pregnancy and check for infection.
   - If your pregnancy test is positive, you are not eligible to participate in the study.
   - If you have a bladder infection, you will receive a prescription for treatment and are eligible to participate in the study once the infection has healed.

5. You will be counseled about HIV testing.

6. About six teaspoons of your blood will be taken from your arm for laboratory tests of your serum chemistries, blood sugar, complete blood counts, hepatitis B and HIV testing. These tests are being done to make sure that these blood tests are normal at the start of the study.

7. You will be given a pelvic examination.
   - During the pelvic exam, the study clinician will use a speculum (an instrument placed into your vagina and expanded), to help the clinician see your vagina and cervix (the opening to the uterus or womb).
   - The clinician will use a cotton swab (“Q-tip”) to take fluid from your vagina and cervix. Tests for gonorrhea, *Chlamydia* and vaginal infections (yeast, trichomonas and bacterial vaginosis) will be done.
   - An examination of your internal pelvic organs will be performed by the clinician who will place one or two gloved fingers into your vagina while gently pressing on the abdomen with the other hand.
   - If you have an infection with yeast or bacterial vaginosis, you will get a prescription for treatment. You will be eligible to participate in the study once you have finished the medication.
   - If you test positive for *Trichomonas* you will receive a prescription for treatment, but you are not eligible to participate in the study.

8. If you are eligible to participate in the study, there will be some limits on you that will begin 72 hours before the next visit and continue until the end of the first part of the study. You will also have these same limits 72 hours before Visit 3 that will continue until after Visit 6 (the Final Visit). Please do not:

- o have sex,
- o masturbate,
- o have someone else give you oral sex,
- o have anal intercourse,
- o douche,
- o put anything other than the tenofovir gel in your vagina including fingers, tampons, sex toys or other objects,
- o use any vaginal hygiene products, lubricants, spermicides, diaphragms or cervical caps.

Visit 2 (Enrollment)

1. This visit should take place within 2 months of your screening visit and will be scheduled when you are not menstruating. Your test results from the previous visit will be reviewed. You will not be enrolled in the study if the tests of your blood chemistries, liver and kidney functions, blood sugar, blood counts, or tests for gonorrhea, *Chlamydia* are abnormal or if you are positive for HIV or Hepatitis B. You will be told to see your own doctor for treatment. If you do not have a place to go for treatment, the research staff will help you find a doctor.

2. Although it is unlikely that you will have tenofovir in your blood, a blood sample will be drawn to make sure that you start the study without tenofovir in your blood. Research staff may insert one needle in your arm that will most likely remain for the next six blood collections to reduce the number of times that you will have the needle inserted.

3. A urine sample will be collected to test for pregnancy.
   - o If your pregnancy test is positive, you will not be enrolled in the study.
   - o If you have symptoms of a bladder infection, the urine sample will be tested for infection.

4. If you have vaginal or genital symptoms or an abnormal exam, you may be tested for gonorrhea, Chlamydia, vaginal infections, syphilis, and/or herpes.

5. A pelvic examination will be performed in which the vulva, vagina and cervix will be examined by the clinician for cuts in the skin. If a deep cut is seen, you will not be enrolled until it has healed.

6. If you are eligible for the study, you will be enrolled and randomly assigned to one of seven time-points for collection of specimens from your cervix and vagina. You will be asked to empty your bladder and bowels and then will be given the first dose to insert in the clinic. After inserting the gel, you will be asked to walk around the examination room for 15 minutes to help spread the gel, after which time you may walk around as you like.

7. You will be asked to try not to empty your bladder or bowel within the first hour after application.

8. Research staff will collect your blood 7 times (at 30 minutes, 1 hour, 2 hours, 4 hours, 6 hours, 8 hours and 24 hours after you spread the gel) to measure how much, if any, tenofovir

gel was absorbed into your blood. You will stay in the clinic until after your 8 hour blood sample is collected.

9. You will return the next morning for the 24 hour blood sample collection, as well as your 24 hour vaginal biopsy (sample of your vaginal tissue) if you were assigned to the 24 hour group. At that time you will be asked if you had any health problems since you left the research center.

10. You will undergo pelvic examination procedures and collection of vaginal samples at your assigned time (this may be when you return the next morning if you were assigned to the 24 hour group). The study clinician will take fluid from the back of your vagina and will brush the endocervix (opening of your cervix) to obtain specimens which will be evaluated to see how much, if any, tenofovir gel is detected in the vaginal fluid and in the endocervix. The study clinician will gently wipe a small area of your vagina with a small cotton swab of warm saline, and then again with a cotton swab of antiseptic to clean it. The clinician will also apply a numbing gel to the area. The study clinician will then remove two small biopsies, about the size of a grain of rice. These samples will be stored until they are evaluated to see how much, if any, tenofovir can be detected in the tissue.

11. You will be told to avoid sexual activity, douching or tampon use for at least one week following the biopsy to allow time for the biopsy sites to heal.

12. You will receive a Participant Guidebook that will give detailed instructions on how to insert the tenofovir, as well as what you can expect during each visit

13. Your next visit will be in approximately one month. There will be some limits on you that will begin 72 hours before the next visit and continue until the end of the study. Please do not:
    - have sex,
    - masturbate,
    - have someone else give you oral sex,
    - have anal intercourse,
    - douche,
    - put anything other than the tenofovir gel in your vagina including fingers, tampons, sex toys or other objects,
    - use any vaginal hygiene products, lubricants, spermicides, diaphragms or cervical caps

14. You may continue normal sexual activity from the period that lasts from one week after this visit, until 72 hours before your next visit, unless you are experiencing severe abdominal pain, bleeding (if more than spotting), foul-smelling discharge, or fever and/or chills.

Visit 3 (Two-week Phase)

1. This visit will take place when you have finished menstruating and are not expected to menstruate for at least the next 15 days. You will be asked if you had any health problems since the last visit.

2. A urine sample will be collected to test for pregnancy.
    - If your pregnancy test is positive, you cannot stay in the study.
    - If you have symptoms of a bladder infection, the urine sample will be tested for infection

3. If you have vaginal or genital symptoms or an abnormal exam, you may be tested for gonorrhea, Chlamydia, vaginal infections, syphilis, and/or herpes.

4. A blood sample will be drawn to measure tenofovir in your blood.

5. A pelvic examination will be performed in which the vulva, vagina and cervix will be examined by the clinician for cuts in the skin and to make sure the biopsy areas have healed. If a deep cut is seen, you will not continue in the study until it has healed.

6. You will be randomly assigned to either once a day or twice a day gel use and will receive study supplies. If you are assigned to once-daily use, you will be instructed to insert the gel in the morning at approximately the same time every day, starting the following day. If you are assigned to twice-daily use, you will be instructed to insert the gel in the morning and evening at approximately the same time every day, starting the following day. In addition, you will be asked to walk around for at least 15 minutes after insertion of the gel and to try not to empty your bladder or bowels within the first hour after application.

7. You will be given a diary card and instructions. You will record the time(s) that you apply the gel each day. If you experience any burning, irritation, pain, itching, etc. while using the gel, you will record that information on the diary card. If you experience any moderate symptoms (you have to stop some normal activities) or severe symptoms (you are unable to perform most normal activities) please call the clinic. If you have these symptoms at any time during this study you may undergo evaluation including laboratory testing, physical examination, and/or visual inspection of your genital area with a colposcope (magnifying lens) as the clinician determines. You will also record any medication that you use while you are in the study on the diary card.

8. Your next visit will be scheduled in one week. You will be told to bring all used gel tubes to this visit. You will be asked to wait until your visit to the research center to insert your morning dose for that day.

Visit 4 (One Week follow-up)

1. A urine sample will be collected to test for pregnancy.
    - If your pregnancy test is positive, you cannot stay in the study.
    - If you have symptoms of a bladder infection, the urine sample will be tested for infection

2. If you have vaginal or genital symptoms or an abnormal exam, you may be tested for gonorrhea, Chlamydia, vaginal infections, syphilis, and/or herpes.

3. The study staff will review your diary card and you will be asked if you had any medical problems since your last visit.

4. A blood sample will be taken to measure how much, if any, tenofovir gel was absorbed into your blood after which you will insert the gel.

5. You will undergo pelvic examination procedures and collection of vaginal samples 4 hours after you insert the gel. The study clinician will take fluid from the back of your vagina and will brush the endocervix (opening of your cervix) to obtain samples which will be evaluated to see how much, if any, tenofovir can be detected in the vaginal fluid and the endocervix.

6. You will be instructed to continue using the study gel as assigned and will be scheduled for your two week follow-up visit. You will be told to bring all used and unused gel tubes to this visit. You will be asked to wait until your visit to the research center to insert your final dose.

<u>Visit 5 (Two Week follow-up)</u>

1. A urine sample will be collected to test for pregnancy.
    - If your pregnancy test is positive, you cannot continue in the study.
    - If you have symptoms of a bladder infection, the urine sample will be tested for infection,

2. If you have vaginal or genital symptoms or abnormal exam, you may be tested for gonorrhea, Chlamydia, vaginal infections, syphilis, and/or herpes.

3. You will be randomly assigned to one of three times (either 4, 8 or 24 hours after you insert the gel), and at your assigned time you will undergo pelvic examination procedures and collection of vaginal samples.

4. The study staff will review and collect your diary card and you will be asked if you had any medical problems since your last visit. If you are <u>not</u> participating in the substudy, you will be given a copy of the back of your diary card to record any symptoms and medications between this visit and Visit 6.

5. A blood sample will be taken to measure how much, if any, tenofovir gel was absorbed into your blood after which you will insert the morning dose of gel. In addition, research staff will collect your blood 7 times (at 30 minutes, 1 hour, 2 hours, 4 hours, 6 hours, 8 hours and 24 hours after you insert the gel) to measure how much, if any, tenofovir gel was absorbed into your blood. Research staff will insert one needle in your arm that will most likely remain for the first six blood collections to reduce the number of times that you will have the needle inserted. Your blood will also be tested for serum chemistries, blood sugar and complete blood counts to make sure that they have not changed since the start of the study.

6. When you return the following morning for the 24 hour blood draw (and your 24 hour biopsy, if you were assigned to the 24 hour time) you will be asked if you had any health problems since you left the research center.

7. At your assigned pelvis examination time (either 4, 8 or 24 hours after you insert the gel), the study clinician will take fluid from the back of your vagina and will brush the endocervix (opening of your cervix) to obtain samples which will be stored and evaluated to see how much, if any, tenofovir can be detected in the vaginal fluid and in the endocervix. The study clinician will gently wipe a small area of your vagina with a small cotton swab of warm saline, and then again with a cotton swab of antiseptic to clean it. The clinician will also apply a numbing gel to the area. The study clinician will then remove two small biopsies, or samples of the vaginal tissue, about the size of a grain of rice. These samples will be stored until they are evaluated to see how much, if any, tenofovir can be detected in the tissue.

8. If you will not be taking part in the substudy, you will be told to avoid sexual activity, douching or tampon use for at least one week following the biopsy to allow time for the biopsy site to heal and will schedule an appointment to return within 1-2 weeks for the final visit.

9. Substudy Participants only: You will not have a Visit 6 in the main study, but instead will be scheduled for Substudy Visit 1. This visit will be scheduled at least 30 days from this visit for when you will not be menstruating and an attempt will be made to schedule the visit in the second half of your menstrual cycle, ideally menstrual cycle day 19-24.

10. You will be told to avoid sexual activity, douching or tampon use for at least one week following the biopsy to allow time for the biopsy sites to heal.

11. You will receive a substudy Participant Guidebook that will give detailed instructions on how to insert the tenofovir, as well as what you can expect during each visit

12. You may continue normal sexual activity from the period that lasts from one week after this visit, until 72 hours before Substudy Visit 1, unless you are experiencing severe abdominal pain, bleeding (if more than spotting), foul-smelling discharge, or fever and/or chills.

13. There will be some limits on you that will begin 72 hours before the next visit and continue until the end of the study. Please do not:

    - have sex,
    - masturbate,
    - have someone else give you oral sex,
    - have anal intercourse,
    - douche,
    - put anything other than the tenofovir gel in your vagina including fingers, tampons, sex toys or other objects,

- use any vaginal hygiene products, lubricants, spermicides, diaphragms or cervical caps.

Visit 6 (Final Visit)

1. You will be asked if you had any medical problems since your last visit. The copy of the symptoms and medications portion of your diary card will be collected.

2. A urine sample will be collected to test for pregnancy
   - If your pregnancy test is positive, you will be followed until the pregnancy reaches outcome.
   - If you have symptoms of a bladder infection, the urine sample will be tested for infection

3. If you have vaginal or genital symptoms or abnormal exam, you may be tested for gonorrhea, Chlamydia, vaginal infections, syphilis, and/or herpes.

4. A pelvic examination will be performed in which your genital area including the vulva, vagina and cervix will be examined by the clinician to make sure the biopsy areas have healed. If a deep cut is seen, you will be followed until it has healed.

5. You will be exited from the study. If you had any medical problems related to the use of the tenofovir gel, the problem(s) will be followed until resolved.

Substudy: 12 hour time-point

Up to 10 participants at the University of Pittsburgh site who have completed the first two phases of the study will be asked to participate in a third phase, a substudy. If you decide to participate, there will be three visits in this phase.

Substudy Visit 1

1. You will be asked if you had any medical problems since your last visit.

2. A urine sample will be collected to test for pregnancy
   - If your pregnancy test is positive, you will be followed until the pregnancy reaches outcome.
   - If you have symptoms of a bladder infection, the urine sample will be tested for infection

3. If you have vaginal or genital symptoms or an abnormal examination, you may be tested for gonorrhea, *Chlamydia,* vaginal infections, syphilis, and/or herpes.

4. A pelvic examination will be performed in which your genital area including the vulva, vagina and cervix will be examined by the clinician to make sure the biopsy areas have healed. If a deep cut is seen, you will be rescheduled until the cut has healed.

5. A blood sample will be drawn to measure tenofovir in your blood

6. You will be given a single-dose to use at home that evening at a specified time, along with a Substudy Diary Card and instructions. You will be instructed to empty your bladder and bowels prior to inserting the dose. You will insert the dose at home and will walk around for 15 minutes to spread the gel, and then may walk around as you like.

7. You will be scheduled to return to the research clinic the following morning.

<u>Substudy Visit 2</u>

1. A urine sample will be collected to test for pregnancy.
   - If your pregnancy test is positive, you cannot continue in the study.
   - If you have symptoms of a bladder infection, the urine sample will be tested for infection,

2. If you have vaginal or genital symptoms or abnormal exam, you may be tested for gonorrhea, Chlamydia, vaginal infections, syphilis, and/or herpes.

3. The study staff will review and collect your diary card and you will be asked if you had any medical problems and/or vaginal activity since your last visit. You will be given a copy of the Diary Card to record any symptoms and medications between this visit and your final substudy visit.

4. Blood samples will be collected approximately 12 hours after you inserted the tenofovir gel the night before to see how much, if any, tenofovir gel was absorbed into your blood.

5. Each participant will also undergo the following pelvic examination procedures at the 12 hour time-point:

   a. The study clinician will take fluid from the back of your vagina and will brush the endocervix (opening of your cervix) to obtain samples which will be stored and evaluated to see how much, if any, tenofovir can be detected in the vaginal fluid and in the endocervix. The study clinician will gently wipe a small area of your vagina with a small cotton swab of warm saline, and then again with a cotton swab of antiseptic to clean it. The clinician will also apply a numbing gel to the area. The study clinician will then remove two small biopsies, or samples of the vaginal tissue, about the size of a grain of rice. These samples will be stored until they are evaluated to see how much, if any, tenofovir can be detected in the tissue.

6. You will be told to avoid sexual activity, douching or tampon use for at least one week following the biopsy to allow time for the biopsy sites to heal and will schedule an appointment to return within 1-2 weeks for the final visit.

Substudy Visit 3

1. You will be asked if you had any medical problems since your last visit. The copy of your Diary Card will be collected.

2. A urine sample will be collected to test for pregnancy
   - If your pregnancy test is positive, you will be followed until the pregnancy reaches outcome.
   - If you have symptoms of a bladder infection, the urine sample will be tested for infection

3. If you have vaginal or genital symptoms or abnormal exam, you may be tested for gonorrhea, *Chlamydia*, vaginal infections, syphilis, and/or herpes.

4. A pelvic examination will be performed in which your genital area including the vulva, vagina and cervix will be examined by the clinician to make sure the biopsy areas have healed. If a deep cut is seen, you will be followed until it has healed.

5. You will be exited from the study. If you had any medical problems related to the use of the tenofovir gel, the problem(s) will be followed until resolved.

Medical records for examinations and/or treatments at this clinic that are related to this study will become part of your study record. Research staff may access your medical records at this clinic for study related information. You may be asked to participate in additional study procedures but you will be asked for your consent at that time. We may try to contact you in person at your home if we are unable to reach you by telephone or mail.

Risks

Risks of the study may include:

1. Genital Exams: You may experience some discomfort or pressure during the exam of your genital area and inside your vagina. You may have mild vaginal spotting/bleeding which should stop shortly after the examination.

2. Blood draw: You may experience temporary discomfort from the needle stick when your blood is taken. You may feel dizzy, faint or lightheaded. You may have bruising, swelling, or infection where the needle goes into your arm.

3. Vaginal Biopsy: You may feel a pinch and/or have some mild pain when the sample is taken. You might also have some bleeding. Bleeding can usually be stopped by applying an iron-containing substance (Monsel's solution) or silver-containing compound (silver nitrate) to the biopsy area which might lead to dark-colored discharge. In rare cases, a stitch might need to be placed to stop the bleeding. You may feel slight soreness or discomfort in your vagina for a day or two after the test. You may experience some vaginal spotting/bleeding and a slight discharge for up to a week

after a biopsy. Rarely, a biopsy can cause an infection or prolonged bleeding. If needed, you can use a sanitary pad for the bleeding. You must avoid having sex, douching, or using tampons for at least a week, and avoid rigorous activity for at least a day after your biopsy to allow time for the biopsy site to heal. You will be given an instruction sheet and should notify the study clinician if you experience vaginal bleeding (if more than spotting), foul-smelling vaginal discharge, fever and/or chills, or severe lower abdominal pain.

4. Tenofovir Gel: It is important to use the study gel as instructed by the staff. You will be putting the gel in your vagina. The gel should not be put in the mouth. The gel used in this study may have side effects. We are still learning about the gel and some of the effects are still unknown. Some possible, more common, side effects that you may experience are dryness, burning, itching, pain, etc. of your genital area during the product use. You may also have discharge if the gel comes out of your vagina or you may experience lower abdominal pain. If the symptoms are moderate (you must stop some normal activities) or severe (you are unable to perform most normal activities), you should call the research site and an appointment will be set up, if necessary. If you cannot come to the clinic on the same day, you will be allowed to stop using the product, wash your vagina with plain warm water, apply medication (if needed) and come to the clinic as soon as possible for evaluation. Some women who used the gel had redness and slight bruising of the genital area seen during examination.

   It is possible that tenofovir gel could be absorbed from the vagina into the blood. In an earlier study of tenofovir gel in women, a very small amount of tenofovir gel was absorbed from the vagina into the blood in about half of the women tested. If the gel is absorbed into the blood, it is not known whether it will cause any bad effects.

   The following side effects have been reported with the use of oral tenofovir pills taken by people who were sick. These side effects may have been because of other medicines that patients were taking or because of their health:

   - Upset stomach, vomiting, gas, loose or watery stools.
   - Dizziness.
   - Abdominal pain.
   - Lack of energy.
   - Kidney damage or failure.
   - Inflammation or swelling and possible damage to the pancreas.
   - Shortness of breath.
   - Rash
   - Low phosphate, a chemical in the blood.
   - Allergic reaction, which may include fever, rash, upset stomach, vomiting, loose or watery stools, abdominal pain, achiness, shortness of breath or a general feeling of illness.
   - Decreased bone mineral density.

The study gel used in this study may not protect you against sexually transmitted infections (STIs) including HIV, the virus that causes AIDS. It is not known what effect the gel could have on the HIV virus. If the virus changes, normal treatment for HIV may not work on the virus. If you get infected

with the HIV virus during the study, the study clinician will provide you a referral for counseling and clinical care.

It is not known if tenofovir gel poses a risk to an embryo or fetus. You will be instructed not to have sexual intercourse with women or men, even with a condom, during the study phases. If you do become pregnant while using tenofovir gel, the study clinician will ask for your consent to receive a copy of medical records related to the pregnancy, its outcome and the health of the newborn infant, if applicable.

5. Inconvenience: Participation in the study may cause an inconvenience to your daily schedule because you will need to come to the clinic for at least 6 different visits and 2 additional blood draws.

There may be other risks not yet identified.

Benefits

There are no direct benefits to you for participating in this study. This study will, however, provide information that may lead to the availability of a new vaginal product for prevention of the transmission of HIV.

You will receive pelvic exams and counseling and testing for HIV and STIs. You will also have tests to check the overall health of your liver, kidneys and blood cells. This study will not provide you with medical care, but study staff will refer you to other available sources of care. If any of your tests show anything that is not normal, you will be referred for follow-up care.

Alternative Treatments

You are not participating in this study because you have a condition requiring treatment. Rather, you have volunteered to participate in order for the investigators to learn more about tenofovir gel. You do not have to participate in this study, if you choose not to.

Costs and Payments

There is no cost to you for participating in this study. You will be compensated for travel, parking, time off work, inconvenience, etc as described in the table below.

Visit	Time Needed	Compensation
1		
2		
3		
4		

5		
6		

If you leave the study early, you will be paid for each completed visit. The study center will process the request for your payment as quickly as possible, but there may be a delay of two to three weeks after the end of participation before you receive the check.

The cost of this study, including administrative fees, payments to volunteers, and investigator support for the visits and tests, is being paid for by CONRAD which is receiving financial support from the International Partnership for Microbicides (IPM). CONRAD also receives financial support from the United States Agency for International Development and from private foundations.

New Information

Any new information obtained during the course of this research that may affect your health, welfare and/or willingness to continue participation in the study will be provided to you in a timely manner.

Confidentiality

Study records that identify you will be kept confidential as required by law. Federal Privacy Regulations provide safeguards for privacy, security, and authorized access to your records. Except when required by law, you will not be identified by name, social security number, address, telephone number, or any other direct personal identifier in study records disclosed outside of the research site. For records disclosed outside of the research site, you will be assigned a unique code number. The results of this research may be presented at meetings or in publications. Your identity will not be disclosed in those presentations.

In conducting this research study, members of the research team will report the results of your study-related health information, medical history, physical examinations and laboratory tests, as described in this consent, to CONRAD, the manager of the study. This information may be further disclosed to Family Health International (FHI) and other contracted organizations.

All health information will be maintained in strict confidence as required by law. However, your health information may be disclosed if required by law. Once your health information is disclosed for research, such as to the financial sponsor, federal privacy laws may no longer protect the information.

By signing this consent form to participate in the study, you are authorizing the research team to share your health information, as described in this consent form.

You have the right to withdraw your authorization for the sharing of your health information but you must do so in writing. Authorization for sharing data that have already been sent to CONRAD or Family Health International (the Data Coordinating Center) cannot be withdrawn. Your authorization for the sharing of your health information for this study will have no expiration date. You have the right to review your records, once the study has concluded.

The study results and informed consent will be retained in your research record for at least six years or until after the study is completed, whichever is longer. At that time either the research information not already in your medical record will be destroyed or information identifying you will be removed from such study results. Any research information in our medical record will become a permanent part of that document and will be kept indefinitely.

Your records may be reviewed and/or copied in order to meet state and/or federal regulations. Reviewers may include, for example, representatives from the Food and Drug Administration, representatives of CONRAD and the Institutional Review Board that oversees this research.

In addition, if your research record is contained in your medical record, you authorize the investigators and representatives from CONRAD to have access to the records.

Ending Your Participation

Your participation is voluntary and you may refuse to participate in or withdraw from the research study at any time. If you choose to withdraw from the study, you must contact the study center, but you may still come to this clinic for health care and will receive exactly the same health care as you would normally receive had you stayed in the study. It may be necessary for research staff to withdraw you from the study if you are unable or unwilling to follow the study instructions, if you develop or are found to have any condition which would make further participation possibly hazardous to your health, or which would interfere with interpretation of the results of the study, or if the investigator determines that continuing in the study would not be in your best interest. If you do withdraw, or are withdrawn, you will be asked to undergo all evaluations necessary for your safety and well-being as determined by clinic staff. You will also be asked to return any unused product and the diary card. At that time we will ask your permission to continue using all information about you that has already been collected as part of the study prior to your withdrawal.

Compensation for Illness or Injury

If you suffer a physical injury or illness as a direct result of participating in this research study, short-term medical treatment may be provided at no cost to you. No money has been set aside for long-term medical care, lost wages, disability, or pain and/or inconvenience due to a research related injury or illness. However, you do not waive any of your legal rights by signing this consent form.

If You Have a Problem or Question

IF YOU HAVE A MEDICAL EMERGENCY: In the case of a medical emergency, you are advised to call your primary care provider or immediately go to the nearest emergency room. Please report this event to the study center as soon as possible but NOT before you have received the necessary medical care.

IF YOU HAVE A HEALTH PROBLEM: If you have a health problem that does NOT require emergency medical care and you think it might be related to your being in this research study, please call the study center.

CONTACT FOR QUESTIONS: If you have questions about this research study you may contact (name and number). If you have questions about your rights as a research subject you may contact (name and number).

Voluntary Consent

I certify that I have read all of this consent form and that I understand it. A copy of this signed consent form will be given to me. My signature below means that I freely agree to participate in this investigational study.

____________________ ____

Date Signature of Participant

____

Printed Name of Participant

Investigator's Statement:

I certify that I have explained to the above individual the nature and purpose of the study, potential benefits, and possible risks associated with participation in this study. I have answered any questions that have been raised and have witnessed the above signature. I have explained the above to the volunteer on the date stated on this consent form.

____________________ ____

Date Signature of Investigator or Designee

____

Printed Name of Investigator or Designee

APPENDIX VIII: DIARY CARD

TITLE: Single Dose and 14-Day Once or Twice-daily Pharmacokinetic Study of the Vaginal Microbicide Agent 1% Tenofovir Gel (CONRAD Protocol A04-095)

PARTICIPANT NUMBER: __________

DATE AND TIME OF GEL INSERTION

INSTRUCTIONS:

1. Complete one row for each day you are in the study. For each day, write the time that you insert the study gel.
2. If you have symptoms or problems on any day, record them on the *Symptoms and Medication* form, on the back.

**Date**<br>**(MM/DD/YY)**<br>(A day is: midnight to 11:59 PM)	**Time that gel was inserted**<br>*Circle AM or PM for each.*<br>Note: Depending on your assigned group, you should only insert the gel once or twice a day. The third column below is in case you insert an evening dose after midnight.			**Comments**
____/____/____	____ : ____ AM<br>PM	____ : ____ AM<br>PM	____ : ____ AM<br>PM	
____/____/____	____ : ____ AM<br>PM	____ : ____ AM<br>PM	____ : ____ AM<br>PM	
____/____/____	____ : ____ AM<br>PM	____ : ____ AM<br>PM	____ : ____ AM<br>PM	
____/____/____	____ : ____ AM<br>PM	____ : ____ AM<br>PM	____ : ____ AM<br>PM	
____/____/____	____ : ____ AM<br>PM	____ : ____ AM<br>PM	____ : ____ AM<br>PM	
____/____/____	____ : ____ AM<br>PM	____ : ____ AM<br>PM	____ : ____ AM<br>PM	
____/____/____	____ : ____ AM<br>PM	____ : ____ AM<br>PM	____ : ____ AM<br>PM	
____/____/____	____ : ____ AM<br>PM	____ : ____ AM<br>PM	____ : ____ AM<br>PM	
____/____/____	____ : ____ AM<br>PM	____ : ____ AM<br>PM	____ : ____ AM<br>PM	
____/____/____	____ : ____ AM<br>PM	____ : ____ AM<br>PM	____ : ____ AM<br>PM	
____/____/____	____ : ____ AM<br>PM	____ : ____ AM<br>PM	____ : ____ AM<br>PM	
____/____/____	____ : ____ AM<br>PM	____ : ____ AM<br>PM	____ : ____ AM<br>PM	
____/____/____	____ : ____ AM<br>PM	____ : ____ AM<br>PM	____ : ____ AM<br>PM	
____/____/____	____ : ____ AM<br>PM	____ : ____ AM<br>PM	____ : ____ AM<br>PM	

SYMPTOMS AND MEDICATION

INSTRUCTIONS:

1. If you have any symptoms that occur during the study, please record them below.
2. If you take any medication to treat your symptoms, please record the type of medication below.

Please call the Study Coordinator if you experience any moderate or severe symptoms (as defined at the bottom of the page) including vaginal burning, itching, pain, spotting or bleeding at any time during product usage.

Symptom	Start date (MM/DD/YY)	End date (MM/DD/YY)	Duration (if it lasts less than 24 hrs)	Severity*	Type of medication
	____/____/____	____/____/____	Hr: ____ Min: ____		
	____/____/____	____/____/____	Hr: ____ Min: ____		
	____/____/____	____/____/____	Hr: ____ Min: ____		
	____/____/____	____/____/____	Hr: ____ Min: ____		
	____/____/____	____/____/____	Hr: ____ Min: ____		
	____/____/____	____/____/____	Hr: ____ Min: ____		
	____/____/____	____/____/____	Hr: ____ Min: ____		
	____/____/____	____/____/____	Hr: ____ Min: ____		
	____/____/____	____/____/____	Hr: ____ Min: ____		
	____/____/____	____/____/____	Hr: ____ Min: ____		
	____/____/____	____/____/____	Hr: ____ Min: ____		
	____/____/____	____/____/____	Hr: ____ Min: ____		

***SEVERITY OF SYMPTOMS:**
1 = Mild (able to do all activities)
2 = Moderate (must discontinue some normal activities)
3 = Severe (unable to perform most normal activities)

APPENDIX IX: DIARY CARD (FOR SUBSTUDY)

TITLE: Single Dose and 14-Day Once or Twice-daily Pharmacokinetic Study of the Vaginal Microbicide Agent 1% Tenofovir Gel (CONRAD Protocol A04-095)

PARTICIPANT NUMBER: __________

DATE AND TIME OF GEL INSERTION

INSTRUCTIONS:

1. The clinic will tell you what time to insert the gel in the evening on the same day of your Substudy Visit 1. This will be about 12 hours before your Substudy Visit 2. Record the actual date and time you inserted the gel.
2. If you have symptoms or problems, record them in the *Symptoms and Medication* section below.

Date (MM/DD/YY)	Time that gel was inserted	Comments
______/______/______	_______ : _______ PM	

SYMPTOMS AND MEDICATION

INSTRUCTIONS:

1. If you have any symptoms that occur during the study, please record them below.
2. If you take any medication to treat your symptoms, please record the type of medication below.

Please call the Study Coordinator if you experience any moderate or severe symptoms (as defined at the bottom of the page) including vaginal burning, itching, pain, spotting or bleeding at any time during product usage.

Symptom	Start date (MM/DD/YY)	End date (MM/DD/YY)	Duration (if it lasts less than 24 hrs)	Severity*	Type of medication
	____/____/____	____/____/____	Hr: ____ Min: ____		
	____/____/____	____/____/____	Hr: ____ Min: ____		
	____/____/____	____/____/____	Hr: ____ Min: ____		
	____/____/____	____/____/____	Hr: ____ Min: ____		

***SEVERITY OF SYMPTOMS:**
1 = Mild (able to do all activities)
2 = Moderate (must discontinue some normal activities)
3 = Severe (unable to perform most normal activities)
